# Kinetic networks identify TWIST2 as a key regulatory node in adipogenesis

**DOI:** 10.1101/gr.277559.122

**Published:** 2023-03

**Authors:** Arun B. Dutta, Daniel S. Lank, Roza K. Przanowska, Piotr Przanowski, Lixin Wang, Bao Nguyen, Ninad M. Walavalkar, Fabiana M. Duarte, Michael J. Guertin

**Affiliations:** 1 Department of Biochemistry and Molecular Genetics, University of Virginia, Charlottesville, Virginia 22903, USA;; 2 Department of Pharmacology, University of Virginia, Charlottesville, Virginia 22903, USA;; 3 Department of Biomedical Engineering, University of Virginia, Charlottesville, Virginia 22903, USA;; 4 Department of Stem Cell and Regenerative Biology, Harvard University, Cambridge, Massachusetts 02138, USA;; 5 Center for Cell Analysis and Modeling, University of Connecticut, Farmington, Connecticut 06030, USA;; 6 Department of Genetics and Genome Sciences, University of Connecticut, Farmington, Connecticut 06030, USA

## Abstract

Adipocytes contribute to metabolic disorders such as obesity, diabetes, and atherosclerosis. Prior characterizations of the transcriptional network driving adipogenesis have overlooked transiently acting transcription factors (TFs), genes, and regulatory elements that are essential for proper differentiation. Moreover, traditional gene regulatory networks provide neither mechanistic details about individual regulatory element–gene relationships nor temporal information needed to define a regulatory hierarchy that prioritizes key regulatory factors. To address these shortcomings, we integrate kinetic chromatin accessibility (ATAC-seq) and nascent transcription (PRO-seq) data to generate temporally resolved networks that describe TF binding events and resultant effects on target gene expression. Our data indicate which TF families cooperate with and antagonize each other to regulate adipogenesis. Compartment modeling of RNA polymerase density quantifies how individual TFs mechanistically contribute to distinct steps in transcription. The glucocorticoid receptor activates transcription by inducing RNA polymerase pause release, whereas SP and AP-1 factors affect RNA polymerase initiation. We identify *Twist2* as a previously unappreciated effector of adipocyte differentiation. We find that TWIST2 acts as a negative regulator of 3T3-L1 and primary preadipocyte differentiation. We confirm that *Twist2* knockout mice have compromised lipid storage within subcutaneous and brown adipose tissue. Previous phenotyping of *Twist2* knockout mice and Setleis syndrome *Twist2*^−*/*−^ patients noted deficiencies in subcutaneous adipose tissue. This network inference framework is a powerful and general approach for interpreting complex biological phenomena and can be applied to a wide range of cellular processes.

Mature adipocytes contribute to a multitude of metabolic processes by regulating energy balance, producing hormones, and providing structural and mechanical support (Rosen and Spiegelman 2006). Adipocyte hyperplasia downstream from increased adipogenesis is associated with pathogenesis of obesity, type 2 diabetes, and cardiovascular disease (van Kruijsdijk et al. 2009; Unamuno et al. 2018). Adipogenic factors represent opportunities for intervention and possible mitigation of obesity-related sequelae (Ghaben and Scherer 2019; Ahmad et al. 2020). Adipocyte maturation is a tightly regulated process involving many chromatin and transcriptional changes downstream from transcription factor (TF) binding (Siersbæk et al. 2011; Tsankov et al. 2015; Thompson et al. 2016; Rauch et al. 2019; Madsen et al. 2020). Although prior studies have extensively characterized the TFs and gene expression changes required for adipogenesis (Rosen and Spiegelman 2006; Lefterova and Lazar 2009; Siersbæk et al. 2012), this work relied on measurements taken hours or days apart on cells undergoing adipogenesis. Molecular events, such as TF binding, chromatin remodeling, and redistribution of RNA polymerase, occur on a time scale of seconds to minutes (McNally et al. 2000; Chen et al. 2014; Duarte et al. 2016). Therefore, previous examinations of adipogenic signaling likely omitted multiple waves of signaling and potential regulatory factors that may be critical to the process.

Molecular genomics assays can query transcriptional events with extremely high temporal resolution. Although each assay delivers a tremendous amount of information, each is limited in the biology that it measures. ChIP-seq directly quantifies chromatin occupancy of proteins, but the assay is dependent upon the availability of antibodies and is limited to a single factor at a time. ATAC-seq and DNase-seq assays quantify chromatin accessibility, which is an indirect measure of regulatory element (RE) activity (Boyle et al. 2008; Buenrostro et al. 2015). Combining accessibility data with TF motif analyses can accurately infer TF binding without the need for factor- and species-specific antibodies (Wu et al. 1979; Vierstra et al. 2020). Kinetic experiments can further increase the sensitivity of inferring dynamic TF binding, because changes in TF binding modulate local chromatin structure and accessibility (Guertin and Lis 2010, 2013; Siersbæk et al. 2011, 2014). However, these assays do not directly inform on changes in transcription and RNA polymerase dynamics. Although RNA-seq is a popular approach for measuring transcription, the assay relies on accumulation of mature RNA species over hours, making it inappropriate for rapid measurements. In addition, it is difficult to deconvolve mechanistic insights from RNA-seq data, which measure secondary and compensatory transcription as well as long-lived RNA species predating initial measurements. Alternatively, nascent transcription profiling with PRO-seq captures RNA polymerase density genome-wide at high spatial and temporal resolution (Kwak et al. 2013). PRO-seq, like RNA-seq, is limited in its ability to identify potential upstream REs and regulatory TFs. Only by combining multiple approaches can one fully capture the signaling dynamics driving transcription regulatory cascades.

Differential TF activity defines cell identity and drives cellular responses to environmental stimuli by enforcing gene regulatory programs (Takahashi and Yamanaka 2006). Sequence-specific TFs bind to conserved motifs (Ptashne 1967) in REs within promoters and enhancers to regulate different mechanistic steps in transcription (Fuda et al. 2009). TFs recruit cofactors such as chromatin-modifying enzymes and general transcription machinery to REs. TFs are generally characterized as activators or repressors based upon their interaction partners, and recent studies more specifically describe TFs based upon their molecular function and which mechanistic steps they regulate (Hah et al. 2011; Danko et al. 2013; Duarte et al. 2016; Scholes et al. 2017; Sathyan et al. 2019; Neumayr et al. 2022). In addition to chromatin opening and RNA polymerase recruitment, many transcription steps are precisely regulated, such as RNA polymerase pausing, elongation, and termination. RNA polymerase II (Pol II) pauses ∼30–50 bp downstream from the transcription start site (TSS) (Rougvie and Lis 1988; Rasmussen and Lis 1993), and the vast majority of genes show promoter-proximal Pol II pausing (Muse et al. 2007; Zeitlinger et al. 2007; Core et al. 2008). Further modifications to the Pol II complex triggers pause release and productive elongation (Marshall and Price 1995). Defining the steps regulated by TFs is necessary to understand how TFs coordinate with one another productively or antagonistically to regulate complex gene expression programs.

Transcriptional networks consist of multiple rapid waves of signaling through time with potential regulatory feedback and signal propagation through activation and repression of regulatory factors. These complex regulatory cascades are not captured in traditional gene regulatory networks. Differentiating one wave from the next requires observations at multiple, closely spaced time points. In this study, we perform ATAC-seq and PRO-seq on 3T3-L1 cells at seven time points within the first four hours of adipogenesis. We incorporate accessibility and transcription changes into a multiwave signaling network and identify TF families driving the regulatory cascade.

## Results

### TFs from at least 14 families are associated with dynamic chromatin accessibility in 3T3-L1 differentiation

**Figure 1.**
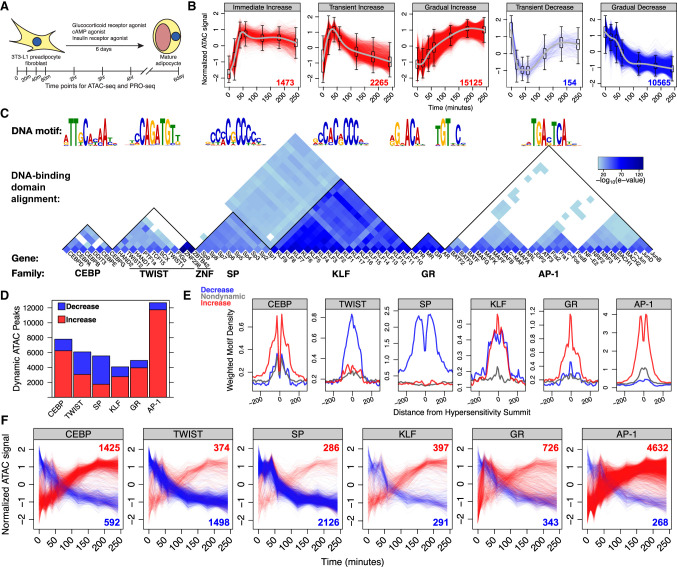
CEBP, TWIST, SP, KLF, GR, and AP-1 TF families drive either increased or decreased chromatin accessibility in adipogenesis. (*A*) Preadipocyte fibroblast 3T3-L1 cells were treated with an adipogenesis cocktail and harvested at the indicated time points for ATAC-seq and PRO-seq experiments. (*B*) Temporal classification of ATAC-seq peaks revealed five major dynamic classes. Each dynamic ATAC peak is a red or blue trace with the number of peaks in the class indicated in the *lower right*; the *x*-axis represents time, and the *y*-axis indicates normalized accessibility. (*C*) De novo motif analysis identified the top six DNA motifs enriched within dynamic peaks. The individual TFs listed in the wedge *below* the DNA sequence logo recognize the respective DNA motifs. The heatmap quantifies the local protein sequence alignment of the DNA-binding domains for the genes, as determined by the Smith–Waterman algorithm (Farrar 2006). (*D*) Dynamic ATAC-seq peaks are classified by the presence of each DNA motif. The red bars represent the number of dynamic ATAC-seq peaks within the immediate increase, transient increase, and gradual increase categories; the blue bars correspond to the transient decrease and gradual decrease classes. (*E*) Red, blue, and gray traces are composite motif densities relative to ATAC peak summits for the increased, decreased, and nondynamic peak classes. The *y*-axis quantifies the density of the indicated position-specific weight matrix, and each motif instance is weighted by its conformity to a composite motif. (*F*) Dynamic traces of peaks that exclusively contain the specified motif indicate that CEBP, GR, and AP-1 associate with increasing accessibility; SP and TWIST associate with decreasing accessibility. Peak traces are colored as in panel *B*. These conclusions are consistent with the reciprocal analysis from panel *E*.

TFs bind promoters and enhancers to modify chromatin structure and influence transcription of nearby genes. To identify dynamic REs and potential TFs that regulate adipogenic differentiation, we induced adipogenesis in 3T3-L1 mouse preadipocytes (see Methods), harvested samples at eight time points, and performed genome-wide chromatin accessibility assays (ATAC-seq) (Fig. 1A). Chromatin accessibility is a molecular measurement used to infer TF binding and RE activity. We identified more than 230,000 accessibility peaks and found that differentiation time is the major driver of variation among the samples (Supplemental Fig. S1A). To address whether the sequencing libraries are saturated, we called peaks on subsets of the total reads and found that the number of called peaks had not reached saturation (Supplemental Fig. S1B). The fraction of reads in peaks (FRiP) varied between 0.2 and 0.3 for most of our ATAC-seq libraries (Supplemental Fig. S1C). However, we note that this score is depressed because we use our total peak set, which includes dynamically accessible peaks that may not be accessible at all time points. Approximately 13% of all peaks change significantly over the time course (Supplemental Fig. S1D). We clustered dynamic peaks based on kinetic profiles (Supplemental Fig. S1E), which resulted in five general response classes (Fig. 1B). To identify candidate sequence-specific TFs that drive RE dynamics, we performed de novo motif analysis on dynamic peaks (Bailey et al. 2015). This approach yielded 14 potential TF family motifs, including CEBP, TWIST, SP, KLF, AP-1, and the steroid hormone receptor motif (Fig. 1C; Supplemental Fig. S1F). TF families comprise multiple proteins containing paralogous DNA-binding domains that recognize very similar sequence motifs (Fig. 1C). For example, multiple factors including androgen receptor, mineralocorticoid receptor, progesterone receptor, and glucocorticoid receptor (GR) bind to the steroid hormone receptor motif. However, GR is the only factor gene that is expressed in 3T3-L1 cells (Supplemental Fig. S1G). Therefore, we refer to the steroid hormone receptor binding consensus sequence as the GR motif. We identified AP-1, CEBP, and GR, which are known positive effectors of adipogenesis (Rubin et al. 1978; Distel et al. 1987; Freytag et al. 1994; Wang et al. 1995; Yeh et al. 1995; Flodby et al. 1996; Tanaka et al. 1997; Moitra et al. 1998; Ramji and Foka 2002; Steger et al. 2010; Siersbæk et al. 2011). Members of the KLF and SP families are known to be associated with both proadipogenic (Inuzuka et al. 1999; Li et al. 2005; Mori et al. 2005; Birsoy et al. 2008; Pei et al. 2011) and anti-adipogenic functions (Tang et al. 1999; Banerjee et al. 2003; Kawamura et al. 2006; Sue et al. 2008). The TWIST family of TFs have previously unappreciated roles in adipogenesis but have been shown to be important for differentiation of other mesenchymal cell types, such as osteoblasts (Yousfi et al. 2001; Bialek et al. 2004). Members of all these factor families are expressed in 3T3-L1 cells (Supplemental Fig. S1G).

TF binding or dissociation from DNA leads to enrichment of cognate motifs in dynamic peaks. The biological functions of the TFs determine whether binding or dissociation results in increased or decreased accessibility. Binding of TFs that recruit activating cofactors, such as histone acetyltransferses or remodeling enzymes that eject nucleosomes, can increase accessibility; dissociation of these factors decreases chromatin accessibility. Likewise, binding and dissociation of factors that recruit deacetylases, repressive methyltransferases, or DNA methyltransferases can affect accessibility. We found that the majority of peaks containing CEBP, KLF, GR, or AP-1 motifs increase accessibility, whereas peaks containing TWIST or SP motifs decrease accessibility (Fig. 1D). We performed the reciprocal analysis and plotted the density of motif instances relative to the summits of increased, decreased, and nondynamic peak classes to confirm the classification (Fig. 1E; Supplemental Fig. S1H). AP-1, GR, and CEBP motifs are strongly enriched around summits of increased peaks, whereas TWIST and SP motifs are enriched around summits of decreased peaks. SP and KLF families have paralogous DNA-binding domains and recognize similar motif sequences; however, we confidently associate chromatin decondensation to KLF factors and chromatin condensation to SP factors (Supplemental Fig. S1I). The SP family is composed of canonical activators (McKnight and Kingsbury 1982); therefore, SP TFs are likely dissociating from the chromatin to reduce accessibility. Although we ascribe opening and closing functions to the KLF and SP families, it is impossible to determine the relative contribution of KLF and SP factors at any individual motif. We believe that the dual enrichment of KLF motifs at both increased and decreased peak summits is owing to erroneous classification of SP-bound REs as KLF-bound REs. This complication is not limited to closely related motifs, as many dynamic peaks contain multiple-factor binding motifs, making it difficult to isolate the contribution of individual factors. To address this complication, we plotted the changes in accessibility at dynamic peaks that contain only a single motif (Fig. 1F). This confirmed that the majority of isolated AP-1, GR, CEBP, and KLF motif-containing peaks increase in accessibility, whereas TWIST and SP motif-containing peaks decrease. The biological interpretation of these results is that the adipogenic cocktail activates members of the AP-1, GR, CEBP, and KLF TF families both directly and through transcriptional activation of family member genes, leading to RE binding and chromatin decondensation. SP and TWIST motifs are associated with decreased accessibility. TFs can act as repressors by binding to chromatin and recruiting chromatin modifiers such as deacetylases. Alternatively, dissociation of an activating TF can lead to gene repression. These results confirm the importance of several TF families and suggest that previously unappreciated TF families, such as TWIST, contribute to adipogenesis.

### SP, NRF, E2F6, KLF, and AP-1 factor motifs are associated with bidirectional transcription at REs

**Figure 2.**
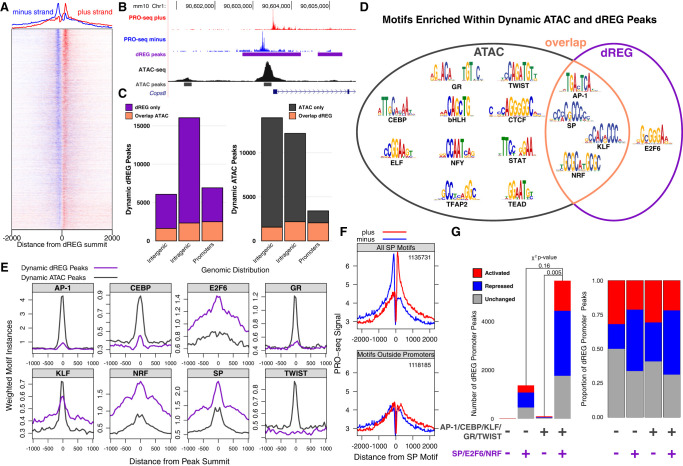
SP, NRF, and E2F6 TF families drive bidirectional transcription dynamics at regulatory regions within gene bodies and promoters. (*A*) The heatmap illustrates more than 200,000 putative REs with a bidirectional transcription signature. (*B*) Both dREG and ATAC-seq identify a RE within the promoter of *Cops8*. The intragenic RE is only identified by its bidirectional PRO-seq signature, whereas the upstream intergenic RE is only identified by ATAC-seq. (*C*) Dynamic ATAC-seq- and dREG-defined REs largely overlap in promoter regions. Intragenic regions are defined based on primary transcript annotation of PRO-seq data; promoters are between 150 bp upstream of and 50 bp downstream from TSSs; and intergenic regions are the remainder of the genome. (*D*) Dynamic ATAC-seq peaks are enriched for a more diverse set of TF motifs than are dynamic dREG peaks. (*E*) Motif density distinguishes TFs associated with dynamic bidirectional transcription from those associated with dynamic accessibility. For example, TWIST and GR motifs are enriched within dynamic ATAC-seq peaks but are rarely found within dynamic dREG peaks. (*F*) SP is only associated with bidirectional transcription at promoters and not distal REs. The *top* plot shows the average normalized PRO-seq signal for plus and minus strands around all 1,135,731 SP motif instances, and the *bottom* plot displays all SP motifs excluding those in promoters (1,118,185). The distinct dual peak profile of bidirectional transcription collapses when only considering SP motifs outside promoters. (*G*) Dynamic bidirectional transcription peaks found in promoters are stratified by the presence or absence of TF motifs. The *left* plot quantifies the total number of peaks, and the *right* plot scales to the proportion of peaks in each category. The *x*-axis factor motif categories are defined by the presence or absence of ATAC-associated factors (AP-1, CEBP, GR, KLF, and TWIST) and dREG-associated factors (SP, E2F6, and NRF). dREG-associated factor motifs are enriched in peaks that decrease bidirectional transcription, suggesting a link between SP, NRF, and E2F6 factors and an early and pervasive decrease in promoter initiation at their target genes.

Coordinate TF binding ultimately results in the recruitment of RNA polymerases and initiation of transcription. In mammals, core promoters and enhancers often lack sequence information that consistently orients initiating RNA polymerases (Core et al. 2014). Therefore, we sought to identify bidirectional transcription signatures as a complement to chromatin accessibility assays to identify REs (Core et al. 2008; Seila et al. 2008; Danko et al. 2013). We captured the short-lived divergent transcripts found at active REs with PRO-seq in parallel with the ATAC-seq adipogenesis time points (Fig. 1A). We used discriminative regulatory-element detection (dREG) to identify peaks of bidirectional transcription from our PRO-seq data (Wang et al. 2019). We identified more than 180,000 dREG peaks (Fig. 2A,B) and an 18% change significantly over the time course (Supplemental Fig. S2A). ATAC-seq and PRO-seq measure distinct but related biological phenomenon; therefore, they identify different but overlapping sets of REs. Approximately 22% of dynamic dREG peaks overlap with dynamic ATAC-seq peaks compared with 20% of dynamic ATAC-seq peaks in the inverse comparison. To further analyze the two classes of REs, we separated the dynamic dREG and ATAC-seq peaks into intragenic, intergenic, and promoter regions (Fig. 2C). Both methods effectively identify REs within promoters (Supplemental Fig. S2B). We find PRO-seq more sensitively detects intragenic REs relative to the other categories, whereas ATAC-seq efficiently detects intergenic REs. We closely evaluated the overlap between ATAC and dREG peaks by plotting PRO-seq signal at ATAC peaks and vice versa (Supplemental Fig. S2C). We observe the distinctive bidirectional transcription signature at ATAC peaks irrespective of whether or not the ATAC peaks intersect dREG peaks. The signature is less intense at ATAC-seq peaks that do not overlap dREG peaks. Likewise, ATAC-seq signal is enriched at dREG-peaks that do not overlap ATAC-seq peaks (Supplemental Fig. S2D). Moreover, dREG peaks within intergenic, intragenic, or promoter regions that do not overlap with ATAC-seq peaks have less bidirectional transcription (Supplemental Fig. S2E). Although we find that bidirectional transcription and accessibility do not perfectly correlate, we are likely underestimating the extent of accessibility and bidirectional transcription overlap.

We sought to identify TFs that drive bidirectional transcription by further characterizing PRO-seq-defined REs. We hypothesized that different sets of TF motifs are enriched within REs defined by ATAC-seq and PRO-seq. For instance, the cognate motifs of TFs that recruit initiation machinery may be preferentially enriched at dREG-defined REs. We performed de novo motif analysis on dynamic dREG peaks and found enrichment of the AP-1, SP, KLF, NRF, and E2F6 motifs (Fig. 2D). Of these, only the E2F6 motif was not also enriched in ATAC-seq peaks. We plotted motif density around the summits of either dynamic ATAC or dREG peaks to further differentiate ATAC- and dREG-defined REs (Fig. 2E; Supplemental Fig. S2F). Of the motifs found de novo in dREG peaks, only E2F6, NRF, and SP were more enriched in dynamic dREG versus dynamic ATAC-seq peaks. We hypothesize that these three factor families regulate bidirectional transcription in adipogenesis. The SP motif is found in >25% of human and mouse promoters, making the SP motif the most enriched *cis*-RE within promoters (Benner et al. 2013). To determine whether divergent transcription signatures found at SP motifs are dominated by SP factors within promoters, we plotted plus- and minus-strand nascent transcription at all SP motif instances (Fig. 2F, top). Indeed, when SP motifs within promoters are removed from the composite input, divergent transcription peaks collapse (Fig. 2F, bottom). We also observe this phenomenon with E2F6 and NRF motifs (Supplemental Fig. S2G), implying that these factors and SP preferentially regulate divergent transcription at promoters. Next, we wanted to determine whether SP, NRF, and E2F6 motifs within the promoters associate with increasing or decreasing divergent transcription. We find that bidirectional transcription tends to decrease in REs with dREG-enriched motifs as opposed to those without dREG-enriched motifs (Fig. 2G). This further supports the previous conclusions that SP and NRF motifs associate with decreases in RE activity (Fig. 1E; Supplemental Fig. S1H). We find distal REs are more likely to show accessibility changes, whereas promoters are more likely to show bidirectional transcription changes.

### Defining predicted TF binding events as *trans*-edges in the network

We determined candidate functional TFs within the set of REs by searching for overrepresented sequence motifs and determining the expression levels of TF family members. However, inferring TF binding from accessibility, motif, and expression data at any individual site remains a challenge (Guertin et al. 2012; Li et al. 2019b). In addition to chromatin accessibility, expression of the TF, and presence of the TF's cognate motif, we leverage the change in accessibility over the time course to infer TF binding and dissociation events in adipogenesis. We term these predicted changes in TF occupancy, which are directed linkages from TFs to REs, as *trans*-edges in our networks. For simplicity, we refer to *trans*-edges as factor binding or dissociation events.

We define the following rules for *trans*-edge inference: (1) The RE must first be defined as an ATAC-seq peak at any time point; (2) the binding motif of the upstream TF must be present in RE; (3) chromatin accessibility must change significantly between two time points to infer binding or dissociation; (4) the direction of accessibility changes must match with the molecular function of the TF as defined in Figure 1; (5) members of the TF family must be expressed at the appropriate time point (e.g., the TF must be expressed at the later time point for binding and the earlier time point for dissociation); and (6) GR, AP-1, and CEBP are directly activated by the adipogenic cocktail, so we infer edges from expressed family members to REs from 0–20 min. We necessitate that the nascent RNA expression of the other TFs changes significantly to infer *trans*-edges from their genic node to an RE node. Mechanistically, TFs have short residency times on DNA, and they are continually binding and dissociating from their sites in vivo (McNally et al. 2000; Chen et al. 2014). When we refer to inferred binding and dissociation within the network, we are strictly referring to overall changes in occupancy at a genomic site within the population of cells.

The following examples highlight implementations of these rules. The *Nr3c1* gene, which encodes GR, decreases expression immediately upon treatment (Supplemental Fig. S3A). We suggest that the rapid transcriptional repression of *Nr3c1* is the reason GR-associated increases in accessibility are transient. Therefore, we restrict binding edges attributed to GR to the first 40 min of the time course. We attribute any significant decreases in accessibility at inferred GR binding REs observed at later time points to dissociation of GR. We label these edges with a *dissociation* attribute. In the case of SP, we find that *Sp1*, *Sp3*, and *Sp4* are all repressed early in the time course (Supplemental Fig. S3B). We hypothesize that the delayed accessibility decrease associated with the SP motifs is owing to transcriptional repression and natural turnover of the SP pool, which result in overall dissociation of SP on chromatin (Fig. 1F). We restrict *trans*-edges for SP to the later part of the time course. Conversely, we observe *Twist2* gene activation early in the time course (Supplemental Fig. S3C). Therefore, we predict that TWIST-associated repression is a result of increased TWIST binding and recruitment of negative cofactors. *Twist2* expression levels have returned to baseline in mature adipocytes (Supplemental Fig. S3D), suggesting that TWIST's effects are transient and can only be captured with an early, high-resolution time course. By focusing only on the REs that change accessibility and integrating with transcription data, we infer TF binding and dissociation events that drive adipogenesis.

We integrated publicly available TF ChIP-seq data sets to assess the performance of *trans*-edge inference. Specifically, we incorporated ChIP-seq profiling of AP-1 (cJun and JunB), KLF (KLF4 and KLF5), CEBPB, and GR (Siersbæk et al. 2011, 2014). All these experiments were performed in 3T3-L1 preadipocytes at 4 h of differentiation. Furthermore, we integrated TWIST2 ChIP-seq data from myoblasts overexpressing 3x-Ty1-tagged TWIST2 protein (Li et al. 2019a). Both the difference in cell type and the disruption of normal TWIST2 function owing to overexpression renders this data set a poor comparison for our system. Nevertheless, we found that 55%–70% of our inferred binding events for these factors overlap called ChIP-seq peaks (Supplemental Fig. S3E). The one exception was for GR, which showed a much lower degree of overlap (35%). This is consistent with our network, which suggests that GR binds and dissociates rapidly from the chromatin at many sites and would not be expected to be bound at the 4-h ChIP-seq time point. Furthermore, CEBP, GR, and KLF REs that show sustained high accessibility (i.e., nonattenuated) displayed a higher degree of overlap with ChIP-seq peaks than those that did not, suggesting that our ATAC-seq dynamics captured fluctuations in factor binding. In addition, we plotted composite ChIP signal at our predicted binding sites and found a strong enrichment in signal at REs that overlap with ChIP-seq peaks (Supplemental Fig. S3F). We also observed a weaker enrichment of ChIP signal around inferred binding events that do not overlap with ChIP-seq peaks, suggesting weaker binding events at these locations were overlooked in ChIP-seq peak calling. However, it is also possible that the enrichment in ChIP-seq signal at these regions reflects underlying accessibility rather than actual factor binding. We note that ChIP assays are limited by antibody specificity, control data sets, and peak thresholding. Regardless, these ChIP-seq data validate the predictive power of using dynamic ATAC signal and the presence of sequence motifs to infer factor binding.

### Proximal changes in accessibility are tightly linked to transcription

**Figure 3.**
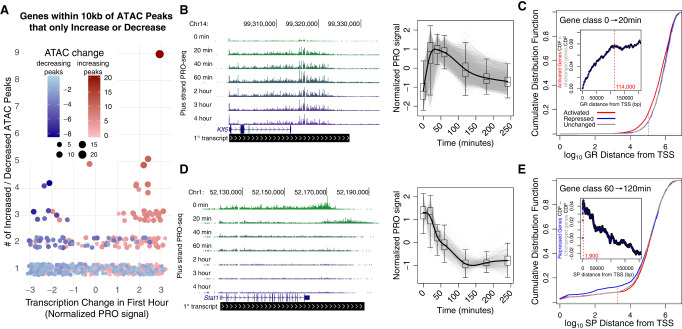
Chromatin accessibility, transcription dynamics, and proximity guide inference of *cis*-edges between REs and target genes. (*A*) Change in gene expression correlates with local accessibility change over the first hour. Each data point represents a gene within 10 kb of either only increased (red) or decreased (blue) peaks. The *y*-axis indicates the number of increased or decreased accessibility peaks near the gene, and the *x*-axis represents the normalized change in gene transcription over the first hour. (*B*) *Klf5* (*left*) is part of a cluster of 1717 immediately and transiently activated genes (gray traces on the *right*). (*C*) Cumulative distribution plots showing distance between GR-bound REs and genes either activated (red), repressed (blue), or unchanged (gray) over the first hour of the time course. The left-shift of the red curve suggests that ATAC-seq peaks with GR motifs are closer to the 20- versus 0-min activated gene class. The *inset* plot reports the difference in cumulative distribution between the activated and unchanged gene classes as distance from the TSS increases. The leveling off of the traces at 114 kb from the TSSs suggests that GR-mediated transcription activation requires GR to bind within 100 kb of the TSS. (*D*) *Stat1* (*left*) is part of a cluster of repressed genes (gray traces on the *right*). (*E*) ATAC-seq peaks with SP motifs are closer to the 60- versus 120-min repressed gene class. Traces converge 1900 bases from the start sites, suggesting that the functional distance of SP-mediated gene repression is within 2 kb of TSSs.

Chromatin accessibility positively correlates with local gene transcription. We confirmed this assertion by quantifying transcription of genes within 10 kb of dynamic ATAC-seq peak sets that exclusively increase or decrease accessibility (Fig. 3A). The majority of genes (63%) with one proximal increasing ATAC-seq peak are activated; likewise, 68% of genes proximal to a single decreasing ATAC-seq peak are repressed. Genes near two or more increased accessibility peaks are much more likely to be associated with transcription activation and vice versa (Fig. 3A). To further validate this association and explore the relationship between RE and target gene distance, we focused on all genes near one dynamic peak and stratified gene/peak pairs based on distance between the TSS and peak summit (Supplemental Fig. S3G–I). The closer the peak and the gene, the more likely gene transcription and peak accessibility correlate in the same direction. This result indicates that proximal REs have a greater impact on gene expression than distal elements. Moreover, we plotted change in gene transcription against distance-scaled local accessibility changes and observed the expected positive correlation between transcription and accessibility at both the early and late phases of the time course (Supplemental Fig. S3J,K). These findings indicate that both accessibility dynamics and distance are important factors when considering the relationship between REs and genes.

We incorporated the distance between REs and genes as well as covariation in their accessibility and transcription to infer functional links, termed *cis*-edges, in our networks. We define *cis*-edges as predicted regulatory relationships between REs and genes. For example, if GR binds a RE within a gene's promoter and induces gene activation, we draw a *cis*-edge between the RE and the gene. Within the network, we assign GR as an attribute to the edge. To confidently infer and annotate *cis*-edges, we must assess whether a class of TFs is associated with increasing or decreasing transcription. Because the distance between a RE and a gene influences the likelihood that accessibility and transcription will covary, we classified the function of a TF class within the context of adipogenesis by determining if peaks with a cognate TF motif are closer to activated or repressed gene classes. We expected that factor families associated with decreases in accessibility, like SP, would be closer to repressed genes on average. To test this hypothesis, we first categorized genes as significantly activated, repressed, or unchanged for each pairwise comparison within the time course. For example, *Klf5* (Fig. 3B, left panel) is one of a subset of 4225 genes immediately activated from 0 to 20 min (Fig. 3B, right panel). Plotting the cumulative distribution function (CDF) for genes against the distance between the closest peak summit and the gene TSS shows that GR peaks tend to be closer to the 4225 activated genes compared with the repressed or unchanged genes (Fig. 3C). To estimate the maximum range that a factor can act, we plotted the difference between the repressed gene class CDF against the unchanged gene class CDF against distance between gene and peak (Fig. 3C, inset). We find that the difference in the CDFs plateaus at ∼114 kb, meaning that inferred GR binding events accumulate at the same rate for activated and unchanged genes at distances >114 kb. This distance constraint represents an empirical observation that suggests a maximum regulatory distance in this system. It is possible that we would detect a different constraint in another cellular context owing to differences in genomic architecture and regulatory environment. Immediately repressed genes like *Stat1* are closer to SP peaks than are control gene sets (Fig. 3D,E). This analysis indicates that SP acts very proximal to its target genes, with an actionable range of <2 kb (Fig. 3E, inset). This finding is consistent with our previous conclusions that decreased SP peaks are primarily found in promoters (Fig. 2F). Our observed maximal distances represent a hypothesized maximal actionable distance for factor activity in 3T3-L1s. However, we anticipate that the optimal distance for most factors is much closer to target genes than these maxima. Therefore, we apply a closer distance threshold when inferring regulatory relationships between individual REs and genes as described in the next section. The lower thresholds increase our confidence in our predicted *cis*-edges.

### Linking REs to target genes

We incorporate these biological principles into logical rules to define *cis*-edge predictions within an adipogenesis network. We develop our rules to maximize confidence in our predicted regulatory interactions. First, a gene and RE must be within 10 kb to infer a *cis*-edge. Second, RE accessibility and gene transcription must covary over the same time range. These two logical rules provisionally link REs and genes, and then we employ additional rules that reflect the biology of individual TFs. For instance, the 10-kb distance metric is made more strict for factors such as SP, for which the functional distance constraint, as determined in Figure 3E, is <10 kb. Temporal rules also influence edge predictions. For instance, GR-bound REs are only significantly closer to genes activated in comparison to the 0-min time point such as the 20-versus-zero comparison (Fig. 3C), meaning that genes activated later in the time course cannot be directly activated by GR binding in the network. Therefore, as with *trans*-edges, we only infer *cis*-edges between GR-bound REs and genes that change early in the time course. Incorporating these observations into our *cis*-edge rules, we infer direct functional relationships between REs, bound TFs, and changes in target gene expression.

### Constrained networks identify genes regulated combinatorially or by individual TF families

Quantifying nascent transcription with PRO-seq maps the position and orientation of RNA polymerase with base-pair resolution. Nascent transcriptional profiling captures engaged RNA polymerase species throughout the genome, including intragenic features such as the proximal promoter and gene body. We can infer regulatory mechanisms of gene sets by quantifying relative changes in RNA polymerase density within the pause region and gene body. For instance, if the rate of RNA polymerase pause release increases between conditions, we expect that the signal in the pause region to decrease and the gene body signal to increase. Previous studies focus on biological systems in which one TF dominates the response, such as ER, HSF, and NF-kB (Hah et al. 2011; Danko et al. 2013; Duarte et al. 2016). In these systems, the composite RNA polymerase signals at activated genes highlight differences in densities between pause and gene body compartments (Hah et al. 2011; Danko et al. 2013; Duarte et al. 2016; Sathyan et al. 2019). A complication in our system is that multiple TFs cooperate to drive transcription changes, making it difficult to identify the target steps (i.e., initiation, pause release) that TFs regulate. To address this complication, we identified genes that are predominantly regulated by a single TF in our network.

**Figure 4.**
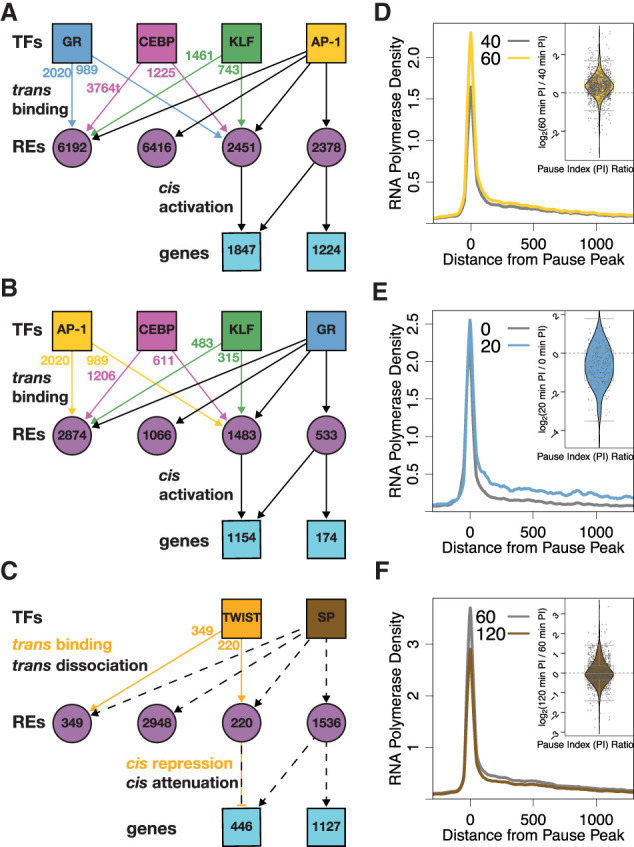
Constrained networks downstream from AP-1, GR, and SP identify genes regulated by individual factors. Simplified networks highlight the number of REs and genes that are combinatorially or individually regulated by AP-1 (*A*), GR (*B*), and SP (*C*). Factors bind/dissociate from REs (purple circles) and regulate genes (blue squares). Colored arrows and numbers indicate the contribution of nonlead factors to RE activity. Combinatorially regulated REs are bound by the lead TF and either one or more of the other TFs. The composite PRO-seq signal is plotted relative to the promoter-proximal pause peak of 1224 genes solely regulated by AP-1 (*D*), 174 genes regulated by GR (*E*), and 1127 genes regulated by SP (*F*). *Inset* violin plots illustrate the change in pause index for the gene set for the indicated time points. Each data point is a gene, and all genes were input from the composite.

We constructed a bipartite network inferring changes in TF binding (*trans*-edges) that regulate downstream changes in transcription (*cis*-edges). Genes and REs can be regulated or bound by either one or a combination of TFs. For example, we constructed a constrained network with RE and gene nodes downstream from individual TFs, including AP-1. In this network, 1224 genes are solely activated by AP-1, and 1847 genes are activated by AP-1 and at least one other factor (Fig. 4A). Most REs downstream from AP-1, both individually and combinatorially bound, are not linked to any downstream genes (12,608 vs. 4829). This network highlights a paradigm in the transcription field that a minority of TF binding events lead to changes in gene expression (Spradling et al. 1975; Westwood et al. 1991). We constructed similar networks for GR (Fig. 4B), SP (Fig. 4C), CEBP (Supplemental Fig. S4A), KLF (Supplemental Fig. S4B), and TWIST (Supplemental Fig. S4C). These networks illustrate the interconnectivity of gene regulation while simultaneously identifying genes that are predominantly regulated by individual factors.

To extract mechanistic information from genes regulated by only one TF, we plotted composite RNA polymerase density from our PRO-seq data around pause peak summits at different time points for the isolated genes (Fig. 4D–F; Supplemental Fig. S4D–F). The resulting traces show the characteristic pause peak centered around zero followed by release of the RNA polymerase into the gene body. Examining RNA polymerase density traces of genes only activated by AP-1 at 60 and 40 min shows an increase in density in the pause region, suggesting increased RNA polymerase recruitment to AP-1-activated genes (Fig. 4D). These time points were chosen because AP-1 peaks are closest to genes activated between 60 and 40 min, suggesting that AP-1 exerts the most transcriptional control during this time range. At first glance, we see a similar result for GR when comparing traces from 0 and 20 min (Fig. 4E). However, the situation becomes more complex when considering the ratio of pause density to gene body density, or pause index (PI). The PI for genes regulated solely by AP-1 increases on average from 40 to 60 min (Fig. 4D, inset). Conversely, the PI for 71% of genes regulated solely by GR decreases, on average, between 0 and 20 min. This suggests that GR primarily activates transcription by inducing pause release (Fig. 4E, inset). The affected step is unique to the factor, as isolated AP-1 genes do not show the decrease in PI from 0 to 20 min observed with isolated GR genes (Supplemental Fig. S4G, inset). Isolated GR genes show increases in PI later in the time course, likely owing to GR dissociation from the genome after the early phase of the time course and an associated decrease in pause release rate (Supplemental Fig. S4H). As for repressed genes, we find a decrease in pause peak and gene body intensity in predicted SP and TWIST target genes (Fig. 4F; Supplemental Fig. S4F). We find that SP target genes show a more symmetrical distribution of PIs (Fig. 4F, inset). Although it is likely that these factors affect Pol II recruitment, we sought to develop a more rigorous approach to determine how changes in initiation and pause release rates can account for observed changes in Pol II density.

### Modeling changes in regulatory transcription steps

**Figure 5.**
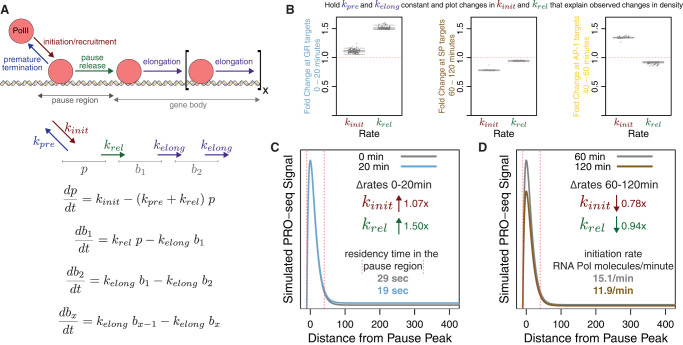
TFs induce changes to transcriptional rate constants. (*A*) The compartment model of transcription contains rate constants for RNA polymerase II (Pol II) initiation/recruitment, premature termination, pause release, and elongation. Polymerase occupancy in the pause or gene body compartment is measured directly from PRO-seq. Differential equations relate rate constants to the rate of change of polymerase density in the compartments. (*B*) We use the compartment model to estimate how rate constants change between 0 and 20 min for the 174 genes regulated solely by GR (*left*), 60 and 120 min for the 1127 genes regulated by SP (*middle*), and 40 and 60 min for the 1224 genes regulated by AP-1 (*right*). For each estimation, we hold *k*_*pre*_ and *k*_*elong*_ constant and calculate values of *k*_*init*_ and *k*_*rel*_ that fit the observed occupancy in the pause and body regions. (*C*,*D*) We simulated composite profiles for a set of parameters from *B*, *left* and *middle*.

We developed a mathematical model to further characterize how TFs target specific steps in the transcription cycle. Our model breaks up the gene unit into two compartments: a pause region and gene body region. PRO-seq directly measures RNA polymerase density within these regions for each gene. We define a series of differential equations to model polymerase density as measured by PRO-seq within the two compartments (Fig. 5A). We establish rate constants representing different transcriptional steps, namely, RNA polymerase recruitment/transcription initiation (*k*_*init*_), premature termination (*k*_*pre*_), pause release (*k*_*rel*_), and elongation (*k*_*elong*_). The values of the rate constants determine the predicted density within the two compartments. We vary the rate constants for *k*_*init*_, *k*_*pre*_, and *k*_*rel*_ over two orders of magnitude and vary the *k*_*elong*_ rate from 600 to 6000 bases per minute to determine the effect on pause and gene body density and how the model compares to observed changes. We make the assumption that *k*_*elong*_ remains constant between time points. Because *k*_*rel*_ and *k*_*init*_ are opposing rates in the model, we cannot distinguish an increase in one rate from a decrease in another. To simplify the model, we keep *k*_*pre*_ constant between time points. We determine how changes in *k*_*init*_ combined with *k*_*rel*_ changes can account for the average density changes for the 174 isolated GR-regulated genes from Figure 4E. A wide range of rate parameters can describe the initial pause and gene body densities, but regardless of the initial rates, a narrow fold-change in these rates can account for the observed changes between time points (Fig. 5B). We find that an approximately 1.07-fold increase in recruitment/initiation and an approximately 1.50-fold increase in pause release explain the changes in compartment occupancy between 0 and 20 min (Fig. 5B, left). We calculated the absolute rate of initiation and residency time of Pol II in the pause region based on the models and plotted a simulated Pol II profile (Fig. 5C). For this simulation, we chose the parameter set with an elongation rate closest to the established consensus rate of approximately 2500 bases per minute (Ardehali and Lis 2009; Jonkers and Lis 2015). Estimated pause residency time drops from 29 sec to 19 sec between 0 and 20 min as a result of the rate constant changes. Taking a similar approach, an approximately 0.78-fold decrease in recruitment/initiation rate with an approximately 0.94-fold change in pause release rate produces observed changes in Pol II occupancy between 60 and 120 min for the 1127 isolated SP genes (Fig. 5B, middle). This corresponds to an initiation/recruitment rate reduction from 15.1 to 11.9 polymerase molecules per minute (Fig. 5D). If SP factors normally stimulate initiation, then mass action would explain dissociation of SP factors upon transcriptional repression of SP genes. Previous studies link SP1 to transcriptional initiation through interaction with the TFIID general TF (Gill et al. 1994). The observed changes in RNA polymerase composite profiles between 40 and 60 min at AP-1 target genes are explained by 1.27- to 1.39-fold increases in initiation rate and 0.85- to 0.93-fold decreases in pause release rate (Fig. 5B, right). These relative changes in *k*_*init*_ and *k*_*rel*_ for AP-1 targets do result in gene activation, but it was unexpected that the profiles are explained by a decrease in *k*_*rel*_. Because composite profiles represent the average of all included genes, it is possible that the composite represents a diverse set of genes that are regulated by different AP-1 family members. We speculate that we could gain a more clear insight if we were able to deconstruct the AP-1 targets and identify gene targets of specific AP-1 factors. The above analyses indicate that we can deconvolve complex transcriptional networks to identify gene targets of individual TFs and determine which steps in the transcription cycle each TF preferentially regulates.

We applied this model and approach to a separate PRO-seq data set of the C7 B cell line treated with dexamethasone to determine whether GR regulates pause release within a different system in which GR is specifically activated. We identified 70 genes activated by dexamethasone treatment. The PI of 80% of these genes decreases between 0 and 60 min (Supplemental Fig. S5A). Compartment modeling of these genes showed that an approximately 1.33-fold increase in pause release rate explained the observed changes in Pol II density at the activated genes (Supplemental Fig. S5B,C). These validation results support the role of GR regulating pause release and highlight the power of predicting the molecular function of TFs within complicated regulatory cascade networks generated from kinetic PRO and ATAC data.

### TFs cooperate to bind REs and activate gene expression

**Figure 6.**
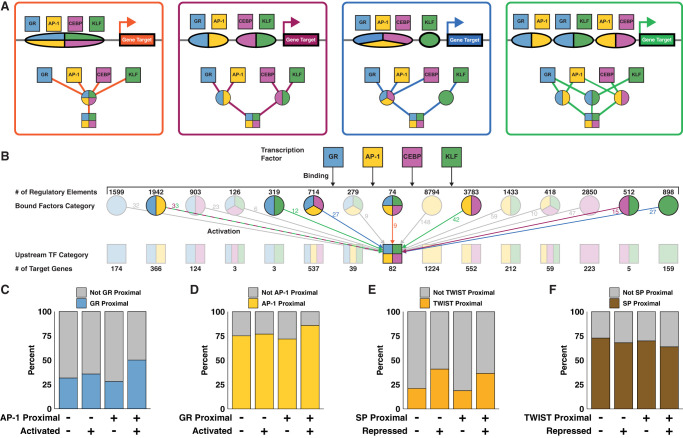
TFs bind REs and activate genes individually and combinatorially. (*A*) Schematics and modular networks illustrate four variations in AP-1, CEBP, GR, and KLF binding patterns that can lead to activation of gene targets: All four factors bind to a single RE (orange); two REs, each bound by two factors (purple); three factors bind one RE and one factor binds another (blue); and a more complex combination with redundant factor contributions at multiple REs (green). (*B*) A wide network depicts cooperation between TFs. *Top* squares indicate GR, AP-1, CEBP, and KLF TF families. Second row circles represent 15 classes of REs, each bound by a different combination of factors. Third row squares represent 15 classes of genes, each regulated by a different combination of factors. There are multiple potential combinations of REs that can produce the same gene class, as illustrated in *A*. The *middle* square representing the 82 genes activated by all four factors is an example of variable regulatory combinations. Colored arrows and numbers correspond to RE combinations as depicted in *A*. (*C*) Genes were sorted into categories based on proximity to AP-1 motifs, proximity to GR motifs, and activation status. We calculated 95% confidence intervals for odds ratios based on contingency tables consisting of genes stratified by their proximity to inferred AP-1 and GR REs and by whether the genes were dynamic. The confidence interval for genes that are not proximal to inferred AP-1 REs is 0.97 to 1.49. The confidence interval for genes that are proximal to inferred AP-1 REs is 2.3 to 2.89. The increase in odds ratio prediction suggests that activated genes proximal to AP-1 are significantly more likely to be proximal to GR than are nonactivated genes. (*D*) The confidence interval for genes not proximal to GR is 0.96 to 1.27. The confidence interval for genes proximal to GR is 1.94 to 2.87. Again, the increase indicates that AP-1 and GR factors coordinate to activate transcription. (*E*) The fraction of repressed genes proximal to TWIST increases regardless of the presence of SP with odds ratio confidence intervals of 2.2–3.07 and 2.21–2.79 when not proximal and proximal to SP peaks, respectively. (*F*) We find a lower proportion of repressed genes proximal to SP motifs in both the presence and absence of TWIST. The odds ratio confidence intervals do not change with the presence of TWIST, going from 0.71–0.89 to 0.64–0.9 when in proximity to predicted TWIST REs.

AP-1, CEBP, GR, and KLF bind REs either individually or in combination in order to activate expression. We classify REs based on the combination of factors that bind and drive accessibility changes. Likewise, we classify genes based on which TFs are immediately upstream in the network. Genes activated by the same combination of factors can be downstream from different classes of REs. We use genes activated by all four of AP-1, CEBP, GR, and KLF to illustrate potential regulatory scenarios. These genes may be downstream from a single RE that binds all factors (Fig. 6A, orange). Alternatively, the gene may be downstream from a pair of REs, each binding two factors (Fig. 6A, purple), three and one (Fig. 6A, blue), or more complicated regulatory schemes (Fig. 6A, green). All 15 possible classes of REs contribute to activation of the 82 genes downstream from AP-1, CEBP, GR, and KLF (Fig. 6B; Supplemental Fig. S6A). The largest population of RE classes is isolated AP-1 peaks with 8794, whereas peaks bound by all activating factors are the smallest category with 74. The distribution of gene classes generally mirrors the distribution of RE classes, with isolated AP-1 genes being the largest class. As discussed above, all factors activate more genes in combination than in isolation. There are comparatively few combinatorially-regulated genes without AP-1 contribution (1924 with AP-1 vs. 149 without). This finding, along with the high number of genes regulated by AP-1, underscores the importance of the AP-1 family in the network. Although the bulk of negatively regulated genes are downstream from either SP or TWIST, ∼20% are affected by both TWIST-mediated repression and SP-mediated attenuation (Supplemental Fig. S6B).

We did not observe a significant relationship between magnitude of RE accessibility change and number of regulatory factors (Supplemental Fig. S6C). We found that the relative change in transcription positively correlates with the number of immediate upstream activators in the network (Supplemental Fig. S6D). Normalizing transcriptional change by local accessibility change eliminates the observed correlation between transcription and number of regulatory factors (Supplemental Fig. S6E). We confirmed this observation by plotting the transcription of all predicted target genes against the total local accessibility stratified by the number of regulatory factors and peaks (Supplemental Fig. S7). We find that more local regulatory peaks, which corresponds to greater total local accessibility, correlate with a greater magnitude of transcription. However, the number of regulatory factors largely does not affect transcription. Therefore, we find that transcription is positively correlated with total local accessibility change, regardless of the number of factors effecting that change. We conclude that if a gene is regulated in the network, the magnitude of expression change is independent from the number of upstream TFs.

Because the degrees of activation and repression are unrelated to the number of upstream factors, we asked if having multiple TFs upstream in the network influences whether a gene is dynamic. To determine whether two TFs cooperate with one another, we considered genes close to dynamic peaks with either a single TF motif or both TF motifs. We determine if the fraction of dynamic and nondynamic genes proximal to a TF is influenced by the presence of another TF. We define TF-proximal genes as genes that are close to dynamic ATAC peaks containing the TF motif. We find that there is no difference between the fraction of GR-proximal activated genes in the absence of AP-1. However, there is an increase in the fraction of activated genes proximal to GR in the presence of AP-1 (Fig. 6C). The reciprocal analysis shows that AP-1 is a more effective activator in the presence of GR (Fig. 6D). These results support the model that AP-1 and GR coordinate with one another to increase the likelihood of gene activation. The repressive factors TWIST and SP do not seem to work together in this way. The fraction of repressed genes proximal to TWIST increases regardless of the presence of SP (Fig. 6E). This suggests that TWIST functions largely independently of SP, supporting our hypothesis that the two TFs result in gene repression through unrelated mechanisms (Supplemental Fig. S3B,C). We find a lower proportion of repressed genes proximal to SP motifs in both the presence and absence of TWIST (Fig. 6F). We speculate that these genes tolerate dissociation of SP and maintain their expression levels despite local decreases in chromatin accessibility. In support of this explanation, we find higher basal transcription and lower magnitude of repression in genes proximal to SP, suggesting these genes are more actively transcribed before loss of SP (Supplemental Fig. S6F,G). These results highlight the complexity of gene regulatory control and how kinetic networks reveal coordinate and independent relationships between TFs.

### Multiwave networks incorporate molecular dynamics and kinetic information

**Figure 7.**
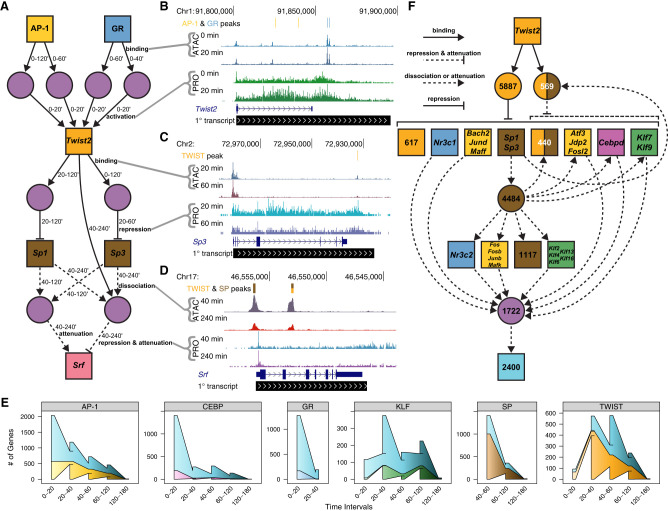
Variations in TF gene expression lead to downstream changes in accessibility and transcription. (*A*) A deep network highlights temporal components of the regulatory cascade. Node and edge characteristics are illustrated as in Figure 4. In addition, we added time interval attributes to respective edges to indicate when REs and genes are changing. (*B*–*D*) UCSC Genome Browser shots indicate accessibility and peaks (*top* three tracks), as well as nascent transcription (next two tracks) dynamics between the indicated time points. (*E*) Wedged bar plots quantify the regulatory kinetics across the time course for indicated factors. The *x*-axis intervals represent the time range in which the indicated number (*y*-axis) of genes are regulated (connected in the network) by the specified factor. Wedges between bars indicate carryover elements from previous time interval, and the outer “wings” represent elements that are not included in the previous time interval. The *top* shaded blue wedges represent genes regulated by multiple factors; *bottom* wedges represent genes that are solely regulated by the indicated factor. (*F*) A *Twist2*-centric network illustrates the high connectivity and influence of *Twist2*.

We further interrogate the adipogenesis gene regulatory network by leveraging temporal information to infer multiple waves of accessibility and transcriptional changes throughout the time course. The importance of TFs can be inferred by the number of predicted direct target genes (Fig. 6) or the total number of connected downstream genes. The latter is captured by temporal multiwave network depictions. We assembled a representative multiwave deep network (Fig. 7A). The differentiation cocktail induces AP-1 and GR binding to thousands of REs to activate thousands of genes; binding at four of these REs results in activation of the *Twist2* gene (Fig. 7A,B). The resulting TWIST2 protein returns to the nucleus and binds hundreds of REs and represses its target genes. Among the hundreds of repressed TWIST2 target genes are the late-acting (40+ min) factors *Sp1* and *Sp3* (Fig. 7C). The decreased occupancy of SP TFs from the genome leads to decreases in RE accessibility and attenuation of gene expression. Our network suggests that SP1/3 dissociation and TWIST2 binding lead to repression of *Srf* (Fig. 7D). We hypothesize that if we were to extend the time course, we would identify the SRF binding motif in REs decreasing in accessibility beyond 4 h as result of attenuated transcription.

Many genes activated in the early phase of the time course are repressed later on, through either active repression or factor dissociation. We detect these negative feedback loops for each activating TF (Supplemental Fig. S8A). About 63% of AP-1, 74% of CEBP, and 80% of GR *cis*-edges are transient. Only 27% of KLF *cis*-edges are attenuated, suggesting that KLF-mediated activation is less transient and less dependent on the extracellular stimuli found in the adipogenic cocktail. Similarly, a minority of TWIST *cis*-edges and no SP *cis*-edges are attenuated, indicating that SP and TWIST factors mediate sustained repression. A much smaller proportion of *trans*-edges are attenuated, implying that accessibility changes downstream from factor binding and dissociation are more stable (Supplemental Fig. S8B) than are changes in nascent transcription.

We find that regulatory potential for each TF varies greatly throughout the time course. AP-1, CEBP, and GR activate the most genes during the initial phase of the time course, indicating that these TFs precipitate the initial wave of signaling during the first 20 min. Transcriptional activation of TWIST and KLF family genes by the initial factors leads to the next wave of signaling after 20 min (Fig. 7B; Supplemental Fig. S8C). We begin to detect changes in accessibility at KLF- and TWIST-bound REs as early as 20 min (Supplemental Fig. S8D); however, these presumptive binding events do not manifest as detectable changes in nascent transcription until 40 min (Fig. 7E). Although we had originally expected changes in accessibility and transcription to be observed concomitantly, these data show that we have the sensitivity to detect changes in RE accessibility before changes in transcription.

In addition to the TFs whose activity is stimulated by the adipogenesis cocktail, we identify transcriptionally regulated TF genes that are highly connected nodes within the network. The *Twist2* gene is the most highly connected node and directly affects accessibility and transcription of thousands of downstream nodes by binding REs and repressing proximal genes (Fig. 7F). TWIST2 acts through intermediate factors, such as SP, AP-1, GR, to repress thousands of additional genes. In the case of SP, TWIST2-mediated repression of *Sp1* and *Sp3* results in SP dissociation and activation attenuation of downstream genes. TWIST2-mediated repression of AP-1 factors causes AP-1 dissociation and attenuation of AP-1-mediated activation. The cumulative result from both direct TWIST2 action and indirect dissociation/attenuation of TWIST2-targeted TF families affects accessibility at 12,662 REs and 4574 genes. We believe that TWIST2 may have been overlooked as an important adipogenic TF because *Twist2* is only transiently activated (Supplemental Fig. S3C), but this kinetic network implicates TWIST2 as a critical intermediary in the adipogenesis cascade.

### TWIST2 represses predicted target genes

**Figure 8.**
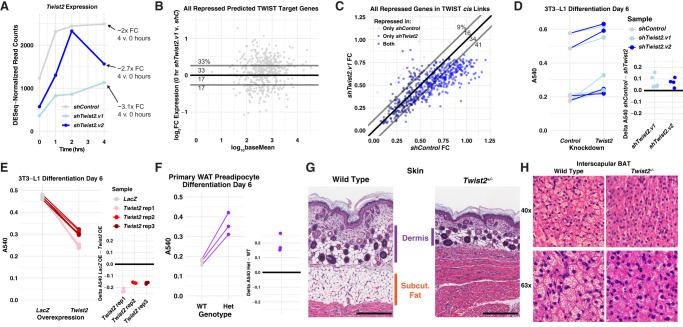
TWIST2 represses target genes and inhibits adipogenesis. (*A*) *Twist2* expression as measured by RNA-seq at indicated time points following differentiation initiation. shTwist2.v1 is a single shRNA construct, whereas shTwist2.v2 is a combination of shRNAs targeting *Twist2*. (*B*) Expression of predicted TWIST2 target genes that are repressed in the RNA-seq time course before addition of the adipogenic cocktail. The *y*-values indicate fold-change of gene expression in *Twist2* knockdown versus control, and *x*-values indicate basal expression of each gene. The majority of genes are expressed to a higher degree in the knockdown samples, suggesting that loss of basal TWIST2 leads to derepression of target genes. (*C*) Each data point represents one of the 520 predicted TWIST2 target genes that are repressed in the RNA-seq time course. We identified the time point comparison for which each gene shows the greatest degree of repression. The *x*-values represent fold-change for the relevant comparison in the shTwist2 samples, and *y*-values represent fold-change for the same comparison in the shControl samples. Predicted TWIST2 target genes largely show a greater degree of repression in the shTwist2 samples owing to a relatively greater magnitude of *Twist2* activation. (*D*) shControl or shTwist2 3T3-L1s were stained with Oil Red O after 6 d of differentiation. Absorbance at 540nm represents total lipid uptake of the cell population. Lines connect control and experimental samples from the same experimental replicate. *Inset* plot displays the difference in absorbance between the indicated shRNA treatment and the control knockdown for each replicate. TWIST2 depletion causes an increase in fat uptake, implicating the TF as a negative regulator of differentiation. (*E*) 3T3-L1s overexpressing either LacZ or TWIST2 were stained with Oil Red O after 6 d of differentiation. TWIST2 overexpression causes a decrease in fat uptake, supporting the conclusion that TWIST2 is a negative regulator of adipogenesis. Lines and *inset* plot are used as in *D*. (*F*) Primary WAT preadipocytes harvested from P3 mice were induced to differentiate for 6 d and stained with Oil Red O. We pooled preadipocytes from animals with the same genotype. Wild-type and heterozygous samples consist of preadipocytes pooled from two and six animals, respectively. The pooled samples were plated in triplicate for the experiment. Preadipocytes harvested from *Twist2*^*+/−*^ pups show increased Oil Red O staining compared with those harvested from wild-type mice. (*G*) Hematoxylin and eosin staining of skin shows collapse of dermal (purple bars) and subcutaneous WAT (orange bar) in P3 *Twist2*^*+/−*^ mice compared with the wild type. Scale bars indicate 200 µm. (*H*) Hematoxylin and eosin staining of interscapular brown fat shows reduced fat droplets (large white/light colored circles) in P14 *Twist2*^*−/−*^ mice compared with the wild type. Images taken at either 40× (*top*) or 63× (*bottom*) magnification.

We tested whether inferred TWIST2-repressed target genes from the network increase expression upon *Twist2* depletion. We used two different shRNA sequences (v1 and v2) to knockdown *Twist2* and harvested RNA for RNA-seq at 0, 1, 2, and 4 h after switching cells into differentiation media. We observed a ∼50%–75% reduction of *Twist2* expression before differentiation (Fig. 8A; Supplemental Fig. S9A). Unlike PRO-seq, RNA-seq requires mature mRNA accumulation above the baseline signal to detect activation, and RNA degradation to detect repression. Therefore, many observed transcriptional changes at the nascent RNA level take much longer to be detected at the mature RNA level. To this point, we find 32,094 total genes expressed in the control RNA-seq data set compared with 13,122 in the PRO-seq data set. This suggests that of all the genes detected by RNA-seq, only ∼40% are actively transcribed. Furthermore, we identify 285 dynamic genes over the first hour of differentiation by RNA-seq versus 9201 by PRO-seq, underscoring the sensitivity of PRO-seq for detecting transcription changes over short time intervals. For RNA-seq analysis, we only focused on the 520 predicted TWIST2 targets that are significantly repressed in both the PRO-seq and RNA-seq time courses. The other 547 predicted TWIST2 target genes from the network are not detected as repressed by conventional RNA-seq, so we would have no power to detect derepression upon *Twist2* depletion. Approximately 66% of the examined genes were expressed at higher levels at the baseline in the *Twist2* knockdown compared with the control, supporting our hypothesis that TWIST2 directly represses the majority of our predicted targets (Fig. 8B; Supplemental Fig. S9B). This result is likely an underestimate of the specificity of our network, because the cells can compensate for chronic RNAi-mediated depletion of *Twist2*.

We then measured the effect of chronic *Twist2* depletion on differentiation-induced transcription. *Twist2* was activated in both the control and the knockdown samples, supporting our PRO-seq results (Fig. 8A; Supplemental Fig. S3C). *Twist2* was more strongly activated in the knockdown sample than in the control (approximately 3.1-fold to approximately twofold). This is likely because the RNAi machinery cannot keep up with the dynamic accumulation of *Twist2* transcripts in the hours following treatment with the differentiation cocktail. Because we inferred that TWIST2 represses target genes in this system, we would expect a greater degree of repression over the time course in the knockdown samples owing to the relatively greater accumulation of TWIST2 protein. This analysis more directly tests the accuracy of predicting TWIST2-target genes compared with chronic knockdown. We find that ∼75% of the predicted targets are repressed to a greater magnitude in the shTwist2 samples compared with the control knockdown (Fig. 8C). We observe supporting results with a second, less effective knockdown (Supplemental Fig. S9C). The above findings support the conclusions from the network and indicate that TWIST2 is a transcriptional repressor of predicted target genes in 3T3-L1 differentiation.

### TWIST2 influences differentiation of 3T3-L1s and primary preadipocytes

To test TWIST2's effect on differentiation of preadipocytes, we depleted TWIST2, induced differentiation of 3T3-L1s, and measured lipid uptake after 6 d of differentiation. We stained differentiated adipocytes with Oil Red O and measured absorbance at 540 nm to quantify lipid uptake. Lipid accumulation is a cellular phenotype that acts as a proxy measurement for adipogenesis. Lipid uptake increased with shTwist2 treatment compared with shControl within each experiment, suggesting that TWIST2 expression negatively regulates differentiation in the 3T3-L1 system (Fig. 8D). As an orthogonal approach, we designed and transduced a tetracycline-inducible 3xFLAG-tagged human *Twist2* construct into 3T3-L1 cells (Supplemental Fig. S9D). After 6 d of differentiation, 3T3-L1s overexpressing *Twist2* showed decreased Oil Red O staining (Fig. 8E; Supplemental Fig. S9E), supporting our previous finding that TWIST2 expression reduces 3T3-L1 differentiation.

Next, we extracted preadipocytes from inguinal white adipose tissue (WAT) of 3 d old *Twist2*^*+/−*^ pups. We induced differentiation in the primary preadipocytes and found that preadipocytes derived from heterozygous mice differentiated to a greater extent than those derived from wild-type mice (Fig. 8F; Supplemental Fig. S9F). The 3T3-L1 and primary cultured preadipocyte results indicate that TWIST2 opposes induced differentiation in both in vitro and ex vivo contexts.

We found that *Twist2*^*+/−*^ mice have a deficiency of dermal and subcutaneous WAT in the skin (Fig. 8G). *Twist2*^−*/−*^ mice have fewer and smaller fat droplets within interscapular brown adipose tissue (BAT) deposits (Fig. 8H). Other groups have reported loss of subcutaneous fat and a paucity of fat storage in *Twist2*^−*/−*^ mice (Šošić et al. 2003; Tukel et al. 2010; Kim et al. 2022). We postulate that TWIST2 acts as a “brake” on adipogenesis, preventing cell exhaustion and apoptosis during the differentiation process. Regulated braking of adipogenesis may be necessary to allow supportive adipose tissues to sufficiently develop in the mouse. Isolated preadipocytes may be able to overcome the additional stress in vitro, but not within their native tissue context.

## Discussion

Kinetic accessibility and nascent transcriptional profiling of developmental cascades can identify key regulatory nodes that may be transiently active, but are nonetheless necessary for proper cellular differentiation. We present an extremely rapid and precise capture of chromatin and transcription changes induced by an adipogenic cocktail. These changes represent the first few waves of differentiation signaling and precipitate the cellular transition process. RE accessibility and gene transcription change within minutes of initiating adipogenesis. By focusing only on dynamically accessible REs, we can infer TF binding and dissociation events that drive adipogenesis without performing hundreds of genomic ChIP experiments. We find a multitude of enriched TF family motifs, many of which have been previously associated with adipogenic REs, including AP-1, GR, KLF, and CEBP (Siersbæk et al. 2014). We do not identify PPARG, the master regulator of adipogenesis (Rosen et al. 2002; Lefterova et al. 2014), as a driver of adipogenic signaling. This agrees with previous conclusions that PPARG does not influence adipogenesis until several days into the process (Nielsen et al. 2008). Stable PPARG activity is indispensable for adipogenesis and maintaining adipocyte identity, but other factors may be critically important and overlooked because their role is transient.

Our method implicates TWIST2 as a novel contributor to adipogenesis. The TWIST subfamily of bHLH TFs homo- and heterodimerize with other bHLH proteins to affect gene expression. Although TWIST family factors all recognize the same DNA motif, different members can act as either activators or repressors. TWIST proteins can repress transcription by nonproductive dimerization with TWIST family activators, competing with TWIST family activators for DNA motifs, or by recruiting chromatin condensers like HDACs to the genome (Hamamori et al. 1999; Gong and Li 2002; Lee et al. 2003; Šošić et al. 2003; Bialek et al. 2004; Hayashi et al. 2007; Koh et al. 2009). Previous studies have implicated TWIST2's role in targeting corepressors (Fu et al. 2011; Kim et al. 2022). Although multiple mechanisms may be at play in our system, we hypothesize that our observed repressive effects are downstream from increased TWIST2 binding. TWIST1 and TWIST2 negatively regulate multiple developmental pathways, including myogenesis, osteogenesis, and myeloid differentiation (Murray et al. 1992; Hebrok et al. 1994; Spicer et al. 1996; Gong and Li 2002; Bialek et al. 2004; Sharabi et al. 2008). The role of the TWIST TF family in adipogenesis is less clear. Although TWIST1 and TWIST2 are known regulators of mature adipose tissue homeostasis, TWIST1 does not affect adipogenesis (Lee et al. 2003; Pan et al. 2009; Dobrian 2012). Homozygous *Twist2* mutations cause Setleis syndrome, a disease characterized by facial lesions lacking subcutaneous fat (Tukel et al. 2010). *Twist2* knockout mice develop such lesions and lack lipid droplets within the liver and brown fat tissue (Fig. 8G,H; Šošić et al. 2003; Tukel et al. 2010). Our in vitro and ex vivo data indicate that TWIST2 acts as a negative regulator of adipogenesis. TWIST2's immediate activation in 3T3-L1 differentiation therefore indicates a negative feedback mechanism to slow differentiation. The TWIST family is a key regulator of the epithelial–mesenchymal transition, further supporting our observation that TWIST2 prevents 3T3-L1 differentiation (Yang et al. 2004). The loss of this negative feedback may result in cell death, leading to the absence of adipose tissue observed in vivo. Even with a well-studied system such as adipogenesis, these methods were able to identify *Twist2* as a novel regulator of the differentiation cascade.

Our networks define gene sets that are predominantly regulated by a single TF. We can track changes in RNA polymerase density within the gene sets to identify the target regulatory steps of individual TFs. Stimulated pause release is an established cause of early gene activation in adipogenesis (Wang et al. 2021). We find that GR is largely responsible for the observed increase in pause release. GR is a well-established activator of gene expression (Vockley et al. 2016), often in combination with AP-1 (Biddie et al. 2011). Other activating factors, including AP-1, increase RNA polymerase recruitment to the gene. By acting on separate steps, GR and AP-1 provide nonredundant stimuli to target genes. We find GR and AP-1 are conditionally dependent upon one another in their potential to activate local genes. A recent study suggests that both AP-1 and CEBP act as pioneer factors that prime the genome for GR-induced transcription activation (Wissink et al. 2019). We find all three of these factor families activate the initial wave of transcription changes, both in combination and in isolation.

We confidently differentiate primary, secondary, and tertiary transcriptional changes by examining multiple, closely spaced time points upon induced adipogenesis. ATAC-seq, ChIP-seq, or chromatin conformation assays alone can only suggest functional relationships between REs and genes (Ren et al. 2000; Lieberman-Aiden et al. 2009). Similarly, PRO-seq and RNA-seq return transcription changes with little information regarding upstream regulation. We define *cis*-regulatory relationships between REs and their target genes by focusing only on ATAC peaks that significantly change accessibility over the time course; likewise, putative target genes are only considered in our network if they change expression over the same time intervals. If REs and genes are proximal to one another and covary in the same direction (i.e., increase in both accessibility and in expression), then we can confidently infer regulatory interactions between TF binding at REs and changes in transcription of the nearby gene. Our bipartite-directed graph networks are unique in the gene regulation field because each edge represents a functional interaction as opposed to an abstract relationship between linked nodes. *Trans*-edges represent binding of TF proteins to cognate DNA elements, and *cis*-edges describe regulatory interactions between REs and target genes. These networks can define gene sets that are predominantly regulated by a single TF and identify the target regulatory steps of the TF. Highly connected nodes in the network are candidate key regulatory hubs in the differentiation cascade. Moreover, these networks ascribe time attributes to each edge, so subgraphs that respect the flow of time are easily extracted from the larger graph. This integrative genomics approach to network construction can be applied to a multitude of cellular responses and transitions to uncover novel biology and new hypotheses.

## Methods

### 3T3-L1 culture and differentiation

3T3-L1 cells were provided by Thurl Harris. 3T3-L1 cells were cultured in high-glucose DMEM (Gibco) supplemented with 10% newborn calf serum, 1% fetal bovine serum (FBS), 100 U/mL penicillin G, and 100 µg/mL streptomycin. We induced adipogenesis ∼3 d after cells reached confluency by switching cells into high-glucose DMEM supplemented with 0.25 µM dexamethasone, 0.5 mM 3-isobutyl-1-methylxanthine, 2.5 U/mL insulin, 10% FBS, 100 U/mL penicillin G, and 100 µg/mL streptomycin (Green and Kehinde 1974; Bernlohr et al. 1984). We collected enough cells at the indicated time points for three replicates of ATAC-seq and PRO-seq.

### ATAC-seq library preparation

We prepared ATAC-seq libraries as previously described (Corces et al. 2017). We trypsinized and collected cells in serum-free growth media. We counted approximately 5 × 10^4^ cells per replicate and transferred them to 1.5-mL tubes. We centrifuged cells at 500*g* for 5 min at 4°C and resuspended the pellet in 50 µL cold lysis buffer (10 mM Tris-HCl, 10 mM NaCl, 3 mM MgCl_2_, 0.1% NP-40, 0.1% Tween-20, 0.01% Digitonin, adjusted to pH 7.4) and incubated on ice for 3 min. We washed the samples with 1 mL cold wash buffer (10 mM Tris-HCl, 10 mM NaCl, 3 mM MgCl_2_, 0.1% Tween-20). We centrifuged at 500*g* for 10 min at 4°C, resuspended cells in the transposition reaction mix (25 µL 2× TD buffer (Illumina), 2.5 µL TDE1 Tn5 transposase (Illumina), 16.5 µL PBS, 0.5 µL 1% digitonin, 0.5 µL 10% Tween-20, 5 µL nuclease-free water), and incubated for 30 min at 37°C. We extracted DNA with the MinElute kit (Qiagen). We attached sequencing adapters to the transposed DNA fragments using the Nextera XT index kit (Illumina) and amplified libraries with six cycles of PCR. We performed PEG-mediated size fractionation (Lis 1980) on our libraries by mixing SPRIselect beads (Beckman) with our sample at a 0.55:1 ratio and then placing the reaction vessels on a magnetic stand. We transferred the right-side selected sample to a new reaction vessel and added more beads for a final ratio of 1.8:1. We eluted the final size-selected sample into nuclease-free water.

### ATAC-seq analyses

We aligned reads to the mm10 mouse genome assembly with Bowtie 2, sorted output BAM files with SAMtools, and converted files to bigWig format with seqOutBias (Li et al. 2009; Langmead and Salzberg 2012; Martins et al. 2018). We called accessibility peaks with MACS2 (Zhang et al. 2008; Gaspar 2018). We sorted reads into peaks using the bigWig R package (https://github.com/andrelmartins/bigwig, v0.2.9) and identified differentially accessible REs with DESeq2 (Love et al. 2014). We cluster dynamic peaks into response groups using DEGreport (https://bioconductor.org/packages/release/bioc/html/DEGreport.html, v1.34.0). We performed de novo motif extraction on dynamic REs with MEME (*e*-value cutoff of 0.01) and used TOMTOM (*e*-value cutoff of 0.05) to match motifs to the HOMER, JASPAR, and UniPROBE TF binding motif databases (Heinz et al. 2010; Bailey et al. 2015; Khan et al. 2018). We use FIMO to identify genome-wide motif occurrences (Cuellar-Partida et al. 2012). We generated DNA sequence logos with ceqLogo (Bailey et al. 2015). We use the bigWig package to assess motif enrichment around ATAC-seq peak summits (https://github.com/andrelmartins/bigwig, v0.2.9).

### PRO-seq library preparation

We performed cell permeabilization as previously described (Mahat et al. 2016). We trypsinized and collected cells in 10 mL ice-cold PBS followed by washing in 5 mL buffer W (10 mM Tris-HCl at pH 7.5, 10 mM KCl, 150 mM sucrose, 5 mM MgCl_2_, 0.5 mM CaCl_2_, 0.5 mM DTT, 0.004 U/mL SUPERaseIN RNase inhibitor [Invitrogen], protease inhibitors [cOmplete, Roche]). We permeabilized cells by incubating with buffer P (10 mM Tris-HCl at pH 7.5, KCl 10 mM, 250 mM sucrose, 5 mM MgCl_2_, 1 mM EGTA, 0.05% Tween-20, 0.1% NP-40, 0.5 mM DTT, 0.004 U/mL SUPERaseIN RNase inhibitor [Invitrogen], protease inhibitors [cOmplete, Roche]) for 3 min. We washed cells with 10 mL buffer W before transferring into 1.5-mL tubes using wide-bore pipette tips. Finally, we resuspended cells in 500 µL buffer F (50 mM Tris-HCl at pH 8, 5 mM MgCl_2_, 0.1 mM EDTA, 50% glycerol, and 0.5 mM DTT). After counting nuclei, we separated cells into 50 µL aliquots with approximately 3 × 10^5^ to 5 × 10^5^ cells each. We snap-froze our aliquots in liquid nitrogen and stored them at −80°C. We prepared PRO-seq libraries as previously described (Sathyan et al. 2019). We included a random eight-base unique molecular identifier (UMI) at the 5′ end of the adapter ligated to the 3′ end of the nascent RNA. We did not perform any size selection in an attempt to not bias our libraries against short nascent RNAs. Raw PRO-seq sequencing files and processed bigWig files were obtained from the NCBI Gene Expression Omnibus (GEO; https://www.ncbi.nlm.nih.gov/geo/) under accession number GSE133147.

### PRO-seq analyses

First we used cutadapt to remove adapters from our reads (Martin 2011). We used fqdedup and the 3′ UMIs to deduplicate our libraries (https://github.com/guertinlab/fqdedup, v1.1.0). Next we removed UMIs and converted reads to their reverse complement with the FASTX-Toolkit (https://github.com/agordon/fastx_toolkit, v0.0.12). As with the ATAC-seq samples, we used Bowtie 2, SAMtools, and seqOutBias to align, sort, and convert reads to bigWig files, respectively (Li et al. 2009; Langmead and Salzberg 2012; Martins et al. 2018). We used primaryTranscriptAnnotation to adjust gene annotations based on our PRO-seq data (Anderson et al. 2020). We queried the bigWig files within the adjusted genomic coordinates with the bigWig R package (https://github.com/andrelmartins/bigwig, v0.2.9) and UCSC Genome Browser Utilities (Kent et al. 2010). We identified differentially expressed genes with DESeq2 (Love et al. 2014). We used dREG to define peaks of bidirectional transcription from our bigWig files (Wang et al. 2019). As with the ATAC-seq samples, we identified overrepresented motifs in dREG-defined REs with MEME and TOMTOM (Bailey et al. 2015). We evaluate motif enrichment around peak summits and polymerase density in the gene body and pause region with the bigWig package (https://github.com/andrelmartins/bigwig, v0.2.9). We define the summit of the pause peak for genes by first identifying the point of maximum density within 1 kb of the TSS. We define the pause region as the 50-bp window around the summit.

### Network construction

The bipartite directional networks with gene and RE nodes were inferred using a data-driven rules-based approach. The first rule to infer *trans*-edges from TF families to individual REs is that the RE must contain the cognate motif for the TF family. The second is that the peak must be dynamically accessible over some part of the time course. The third is that at least one gene encoding a member of the TF family must be expressed and activated (or in the case of SP, repressed) over the same time range. We restrict *trans*-edges attributed to GR to the first 40 min of the time course for reasons discussed in the text. Similarly, we do not draw edges from SP before 40 min. Next, we drew *cis*-edges between REs and proximal genes based on a different rule set. First, REs need to be within 10 kb of gene bodies as defined by primary transcript annotation of our PRO-seq data. We used BEDTools to find gene–RE pairs that satisfied this rule (Quinlan and Hall 2010). Next, the peak and the gene need to covary in accessibility and transcription during the same time range. For example, a gene must be activated at the same time as its local RE is increasing in accessibility. We refined the distance requirements by incorporating constraints from our CDF analysis. For each activating factor (AP-1, CEBP, GR, KLF), we find a set of pairwise comparisons within the time course for which factor REs are significantly closer to activated than nondynamic genes. We find a similar set of comparisons for repressive factors (SP, TWIST). For a gene to be linked to a factor RE with a *cis*-edge, we require that the gene must be dynamic in at least one of the comparisons identified by the CDF analysis for that factor. In addition, our CDF analysis also identifies the maximum distance between a factor RE and a regulated gene for each comparison. The RE and the gene's TSS must be within the relevant distance threshold defined by the CDF.

## Data access

All raw and processed sequencing data generated in this study have been submitted to the NCBI Gene Expression Omnibus (GEO; https://www.ncbi.nlm.nih.gov/geo/) under accession numbers GSE150492 (3T3-L1 ATAC-seq), GSE219041 (C7 cells + dexamethasone PRO-seq), and GSE219051 (shTwist2 3T3-L1 RNA-seq). All code used for data analysis can be found at GitHub (https://github.com/guertinlab/adipogenesis) and as Supplemental Code.

## Supplementary Material

Supplemental Material
