# Supplementary material for: Kinetic networks identify TWIST2 as a key regulatory node in adipogenesis
Source: Genome Res. 2023 Mar;33(3):314–31. doi: 10.1101/gr.277559.122 (PMC10078291; doi:10.1101/gr.277559.122)
Supplement: Supplemental Material [file supp_33_3_314__DC1.html]

Kinetic networks identify TWIST2 as a key regulatory node in adipogenesis — Supplemental Material 

# Kinetic networks identify TWIST2 as a key regulatory node in adipogenesis

## Supplemental Material

- Supplemental\_Code.pdf
- Supplemental\_Figures\_and\_Methods.pdf
