# Supplementary material for: Kinetic networks identify TWIST2 as a key regulatory node in adipogenesis
Source: Genome Res. 2023 Mar;33(3):314–31. doi: 10.1101/gr.277559.122 (PMC10078291; doi:10.1101/gr.277559.122)
Supplement: Supplemental Material [file supp_gr.277559.122_Supplemental_Code.pdf]

An Accessibility and Nascent Transcription Network for Predicting Drivers of 3T3-L1 Adipogenesis

Arun B. Dutta, Bao H. Nguyen, and Michael J. Guertin

February 5, 2023

A step-by-step procedure for analysis and integration of ATAC-seq and PRO-seq data from multiple time points over the first four hours of 3T3-L1 differentiation. For the most recent version of this vignette please visit `https://github.com/guertinlab/adipogenesis`.

Contents

1 Introduction **2**

2 ATAC-seq Analysis **2**
2.1 Download Raw ATAC-seq Sequencing Files From GEO . . . 2
2.2 Align Raw Sequencing Files to the Genome . . . 2
2.3 Convert .bam Files to .bigWig files . . . 3
2.4 Peak calling . . . 4
2.5 Sorting reads into peaks and PCA . . . 4
2.6 Defining and clustering dynamic peaks . . . 5
2.7 Post-clustering processing . . . 7
2.8 Identifying enriched TF binding motifs . . . 9
2.9 Sorting motifs into families . . . 12
2.10 Separating KLF and SP motif instances . . . 16
2.11 Create ATAC data frame . . . 19
2.12 Plotting motif density around ATAC peak summits . . . 22

3 PRO-seq analysis **24**
3.1 Download, align, and process data files . . . 24
3.2 Primary transcript annotation . . . 25
3.3 Calling dynamic genes . . . 29
3.4 Normalizing PRO-seq data for browser . . . 34

4 Integrating accessibility and transcription **34**
4.1 Transcription of genes only near increased or decreased peaks . . . 34
4.2 Evaluating transcription v. accessibility across the genome . . . 36
4.3 Using cumulative distribution functions to assess functional distances . . . 38
4.4 Inferring *trans* and *cis*-edges . . . 41
4.5 Illustrating polymerase density and calculating pause indices . . . 46
4.6 Odds ratio analyses . . . 50
4.7 Attenuated edges . . . 52
4.8 Time networks . . . 55

5 Compartment modeling **56**

6 shTwist2 RNA-seq analysis **56**
6.1 Download .fastq files . . . 56
6.2 Aligning samples to the mouse genome . . . 57
6.3 Create read counts table and plot PCA . . . 58
6.4 Identifying differentially expressed genes across different comparisons . . . 60

6.5 Exploring effect of chronic Twist2 depletion on basal transcription of predicted target genes 61

List of Figures

1 Introduction

The following pipeline is designed to compile on the UVA Rivanna High-Performance Computing system. While most tools and packages are available through Rivanna, you will need to install some manually. To transfer files into and out of Rivanna, use sftp `https://www.rc.virginia.edu/userinfo/rivanna/logintools/cl-data-transfer/`. Remember to modify the directory path for all relevant scripts to match your /scratch/user path.

2 ATAC-seq Analysis

2.1 Download Raw ATAC-seq Sequencing Files From GEO

The ATAC-seq files can be found on the NCBI's Gene Expression Omnibus (GEO) at accession number GSE150492 (`https://www.ncbi.nlm.nih.gov/geo/query/acc.cgi?acc=GSE150492`). The code chunk below will download a table of SRA accession numbers from github and incorporate them into one .slurm file per sample. For each sample we concatenate a header document (downloaded from github and reproduced below) with some new lines into a .slurm file that can be submitted to Rivanna's job manager so that each file can be downloaded in parallel. If working locally, you can set up a loop to download each file individually, but that will take much longer. We use the fasterq-dump command from the sratoolkit to download our files. This command will automatically split each sample into paired end 1 and paired end 2 files with the suffix '_1.fastq' and '_2.fastq' respectively.
header.txt:

```
#!/bin/bash
#SBATCH -n 1
#SBATCH -t 12:00:00
#SBATCH -p standard
#SBATCH -A janeslab
```

script for downloading .fastq files:

```
cd /scratch/abd3x/ATAC

wget https://github.com/guertinlab/adipogenesis/raw/master/Pipeline_ATAC/misc_scripts/ATAC_SRA_table.txt
wget https://github.com/guertinlab/adipogenesis/raw/master/Pipeline_ATAC/misc_scripts/header.txt

echo 'Downloading .fastq'

cat ATAC_SRA_table.txt | while read line
do
sra=$(echo $line | awk '{print $1}')
time=$(echo $line | awk '{print $2}')
rep=$(echo $line | awk '{print $3}')
echo '#SBATCH -o download.'$time'm.rep'$rep'.out' > temp.txt
echo 'module load sratoolkit/2.10.5' > temp2.txt
echo 'fasterq-dump '$sra' -o ATAC_'$time'm_rep'$rep > temp3.txt
echo 'echo DONE' > temp4.txt
cat header.txt temp.txt temp2.txt temp3.txt temp4.txt > download.${time}m.rep$rep.slurm
sbatch download.${time}m.rep$rep.slurm
rm temp.txt
rm temp2.txt
rm temp3.txt
rm temp4.txt
done
```

2.2 Align Raw Sequencing Files to the Genome

As above we will write a script to loop through our samples and incorporate each one into a .slurm script for aligning to the reference mouse genome. For each sample we align the two paired end files to the mm10 mouse genome assembly. We use the Bowtie2 alignment tool (`https://bowtie-bio.sourceforge.net/bowtie2/index.shtml`). Rivanna hosts a Bowtie2-formatted index that we can reference. If you're working locally you'll have to build your own index. After alignment with Bowtie2, we do some quality control and remove any duplicate reads so they do not throw off the analysis.
align_footer.txt:

```
module load gcc/9.2.0 bowtie2/2.2.9 samtools/1.12
name=$(echo $fq | awk -F"_1.fastq" '{print $1}')
echo $name
```

```
echo 'aligning to mouse genome'
bowtie2 --maxins 500 -x /project/genomes/Mus_musculus/UCSC/mm10/Sequence/Bowtie2Index/genome \
-1 $fq -2 ${name}_2.fastq -S ${name}_smp.sam
echo 'quality filter and remove duplicate amplicons'
samtools view -b -q 10 ${name}_smp.sam | samtools sort -n - | \
samtools fixmate -m - - | samtools sort - | samtools markdup -r - ${name}_rmdup.bam
rm ${name}_smp.sam
echo 'zipping .fastq'
gzip ${name}*fastq
echo 'Done'
```

script for generating and submitting alignment .slurm files:

```
cd /scratch/abd3x/ATAC

wget https://github.com/guertinlab/adipogenesis/raw/master/Pipeline_ATAC/misc_scripts/align_footer.txt

for fq in *_1.fastq
do
name=$(echo $fq | awk -F"_1.fastq" '{print $1}')
echo '#SBATCH -o align.'$name'.out' > temp.txt
echo 'fq='$fq > temp2.txt
cat header.txt temp.txt temp2.txt align_footer.txt > align.$name.slurm
sbatch align.$name.slurm
rm temp.txt
rm temp2.txt
done
```

2.3 Convert .bam Files to .bigWig files

We will do two things with the .bam files generated by the previous step. First of all we will convert the .bam files to .bigWig files so we can sort the reads into our called peaks (the next section). Again we will use a script to generate one .slurm file per sample so we can parallelize the conversion process. We use the seqOutBias program to convert our .bam files to .bigWig files (`https://guertinlab.github.io/seqOutBias/`). This program requires a tallymer file for the genome under investigation. Generating this tallymer file takes a long time (multiple hours) but only needs to be done once per genome. We will generate all our .slurm file but only submit one, which will generate the tallymer file. Following completion of that job we will submit all the others and generate our .bigWig files. This step can be done in parallel with the next section, which is calling peaks.
bigwig_convert_footer.txt:

```
module load gcc/7.1.0 seqoutbias/1.3.1
name=$(echo $bam | awk -F"_rmdup.bam" '{print $1}')
echo $name
echo 'converting to .bigWig'
seqOutBias /project/genomes/Mus_musculus/UCSC/mm10/Sequence/WholeGenomeFasta/genome.fa \
$bam --skip-bed --no-scale --bw=$name.bigWig --only-paired --shift-counts --read-size=38
echo 'Done'
```

script for generating and submitting bigwig conversion .slurm files:

```
cd /scratch/abd3x/ATAC

wget https://github.com/guertinlab/adipogenesis/raw/master/Pipeline_ATAC/misc_scripts/bigwig_convert_footer.txt

echo 'Converting .bam files to .bigWig files'

for bam in *_rmdup.bam
do
name=$(echo $bam | awk -F"_rmdup.bam" '{print $1}')
echo '#SBATCH -o bigwig.convert.'$name'.out' > temp.txt
echo 'bam='$bam > temp2.txt
cat header.txt temp.txt temp2.txt bigwig_convert_footer.txt > bigwig.convert.$name.slurm

rm temp.txt
rm temp2.txt
done

#pick one .slurm file to submit to generate tallymer for the mm10 mouse genome assembly
file=$(ls bigwig.convert.*slurm | head -1)
sbatch $file

#when this job is done running (check the associated .out file for 'DONE'), then submit the rest
for file in bigwig.convert.*slurm
do
sbatch $file
done
```

2.4 Peak calling

Below is a .slurm script that, upon submission, will use the MACS2 software to call peaks (`https://pypi.org/project/MACS2/`). We generate one big peaks file from all of our .bam files. We use bedtools to remove 'blacklisted' regions from our peak set. Blacklisted regions are genomic loci that are found in many sequencing datasets despite not reflecting the biology under observation. Finally we take a 200 bp window around the summits of those peaks so that all peaks are the same size. The summit of the peak represents putative transcription factor binding sites.
peak_calling.slurm:

```
#!/bin/bash
#SBATCH -n 1
#SBATCH -t 24:00:00
#SBATCH -o peak.calling.out
#SBATCH -p largemem
#SBATCH -A janeslab

module load macs2/2.2.7.1 gcc/9.2.0 bedtools/2.29.2

#Call peaks
echo 'Calling Peaks'
macs2 callpeak -t *rmdup.bam -f BAMPE -n ATAC --outdir peak_calling --keep-dup 50 -q 0.05

#Download blacklisted mm10 regions
wget https://github.com/Boyle-Lab/Blacklist/raw/master/Pipeline_ATAC/misc_scripts/lists/mm10-blacklist.v2.bed.gz
gunzip mm10-blacklist.v2.bed.gz
mv mm10-blacklist.v2.bed mm10.blacklist.bed

#Remove blacklisted regions
cd peak_calling
bedtools subtract -a ATAC_summits.bed -b ../mm10.blacklist.bed > ATAC_summits_bl_removed.bed
awk '{OFS="\t";} {print $1,$2-99,$3+100,$4,$5}' ATAC_summits_bl_removed.bed > ../peak_windows.bed
echo 'Done'
```

submit peak calling job

```
sbatch peak_calling.slurm
```

2.5 Sorting reads into peaks and PCA

Our next step is to sort our reads from individual samples into peaks in R to generate a counts table. We will also generate a DESeq-normalized counts table and perform a principal components analysis (PCA). A PCA is an easy way to test similarity of data sets. This is equivalent to Supplemental Fig. 1A in the paper. Replicate samples cluster together. Principal component 1 explains the vast majority (91%) of the variation among the samples. Note that the samples separate along principal component 1 based on their time of treatment. This result indicates that the data appropriately reflect the experimental conditions. The PCA can be found in Supplemental Figure 1A.

```
library(bigWig)
library(DESeq2)
library(ggplot2)
source('https://raw.githubusercontent.com/mjg54/znf143_pro_seq_analysis/master/Pipeline_ATAC/misc_scripts/docs/ZNF143_functions.R')

get.raw.counts.interval <- function(df, path.to.bigWig, file.prefix = 'H') {
  df = df[,1:5]
  vec.names = c()
  inten.df=data.frame(matrix(ncol = 0, nrow = nrow(df)))
  for (mod.bigWig in Sys.glob(file.path(path.to.bigWig,
                                        paste(file.prefix, "*.bigWig", sep ='')))) {
    factor.name = strsplit(strsplit(mod.bigWig, "/")[[1]][length(strsplit(mod.bigWig, "/")[[1]])], '\\.')[[1]][1]
    print(factor.name)
    vec.names = c(vec.names, factor.name)
    loaded.bw = load.bigWig(mod.bigWig)
    mod.inten = bed.region.bpQuery.bigWig(loaded.bw, df[,1:3])
    inten.df = cbind(inten.df, mod.inten)
  }
  colnames(inten.df) = vec.names
  r.names = paste(df[,1], ':', df[,2], '-', df[,3], sep='')
  row.names(inten.df) = r.names
  return(inten.df)
}

directory = '/scratch/abd3x/ATAC'
setwd(directory)
peaks = read.table("/scratch/abd3x/ATAC/peak_windows.bed", header = F, sep = "\t")
```

```r
raw.read.counts = get.raw.counts.interval(peaks, directory, file.prefix = 'ATAC')
save(raw.read.counts, file= 'raw.read.counts.Rdata')
########
sample.conditions = sapply(strsplit(as.character(colnames(raw.read.counts)), 'ATAC_'), '[', 2)
sample.conditions = sapply(strsplit(sample.conditions, '_rep'), '[', 1)

sample.conditions = factor(sample.conditions, levels=c("0m","20m","40m","60m","120m","180m","240m"))

deseq.counts.table = DESeqDataSetFromMatrix(raw.read.counts, as.data.frame(sample.conditions), ~ sample.conditions)

dds = DESeq(deseq.counts.table)

#normalized counts table
normalized.counts.atac = counts(dds, normalized=TRUE)
save(normalized.counts.atac,file='normalized.counts.atac.Rdata')

#PCA
rld = rlog(dds, blind=TRUE)

pca.dat = plotPCA(rld, intgroup="sample.conditions", returnData=TRUE)

pca.dat$rep <- sapply(strsplit(pca.dat$name, '_rep'), '[', 2)

percentVar = round(100 * attr(pca.dat, "percentVar"))

colors <- c('#d64e12','#f9a52c','#efdf48','#8bd346','#60dbe8','#16a4d8','#9b5fe0')
alphas <- c(0.33,0.67,1.0)

pca.dat$group <- factor(pca.dat$group,levels=c("0m","20m","40m","60m","120m","180m","240m"))

pdf(file='PCA_atac.pdf',width = 6,height = 5)
print(
  ggplot(pca.dat, aes(x=PC1, y=PC2,color=sample.conditions,alpha=rep)) +
    geom_point(size=4,stroke=0) +
    #geom_violin(alpha=0.5) +
    #geom_jitter(shape=16, col = 'light grey', position=position_jitter(0.2)) +
    theme_minimal() +
    labs(y = paste0('PC2 ',percentVar[2],'% variance'),
         x = paste0('PC1 ',percentVar[1],'% variance'),
         title=NULL,
         color='Treatment',
         alpha='Replicate') +
    #color = 'Near',alpha='Magnitude Total \n ATAC Change') +
    theme(
      panel.grid.minor = element_blank(),
      plot.title = element_text(size=12,face='bold',hjust = 0.5),
      axis.ticks = element_blank(),
      axis.text.y = element_text(size=11,color='black',face='bold'),
      axis.text.x = element_text(size=11,color='black',face='bold'),
      axis.title = element_text(size=12,color='black',face='bold'),
      legend.text = element_text(size=8,color='black',face='bold'),
      legend.title = element_text(size=9,color='black',face='bold')) +
    #coord_cartesian(ylim = c(0, 1.05*max(plot.df$expression))) +
    scale_alpha_manual(values=alphas) +
    scale_color_manual(values=colors)
)
dev.off()
```

2.6 Defining and clustering dynamic peaks

In this chunk we use the likelihood ratio test to identify peaks that are dynamic over the whole time course. We also define a set of highly accessible but not dynamic peaks for future comparisons. We save the nondynamic and full peak sets as .bed files. We will save the dynamic peak sets after clustering. We also plot the number of dynamic and nondynamic peaks for the time course. This is equivalent to Supplemental Fig. 1D in the paper. The numbers are slightly different due to change in software versions and because this plot counts all dynamic peaks, not just those sorted into clusters.

```r
library(lattice)
library(DEGreport)
library(DESeq2)
library(ggplot2)

load('raw.read.counts.Rdata')

sample.conditions = sapply(strsplit(as.character(colnames(raw.read.counts)), 'ATAC_'), '[', 2)
sample.conditions = sapply(strsplit(sample.conditions, '_rep'), '[', 1)

sample.conditions = factor(sample.conditions, levels=c("0m","20m","40m","60m","120m","180m","240m"))

deseq.counts.table = DESeqDataSetFromMatrix(raw.read.counts, as.data.frame(sample.conditions), ~ sample.conditions)
```

```
dds = DESeq(deseq.counts.table)

dds.lrt = DESeq(dds, test="LRT", reduced = ~ 1)
res.lrt = results(dds.lrt)
save(res.lrt, file = 'res.lrt.Rdata')

padj.cutoff = 0.00000001 #1e-8

siglrt.re = res.lrt[res.lrt$padj < padj.cutoff & !is.na(res.lrt$padj),]

rld = rlog(dds, blind=TRUE)

#for clustering (next step)
rld_mat <- assay(rld)
cluster_rlog = rld_mat[rownames(siglrt.re),]
meta = as.data.frame(sample.conditions)
rownames(meta) = colnames(cluster_rlog)
save(cluster_rlog, meta, sample.conditions, file = 'cluster_rlog_pval_1e8.Rdata')

#define set of highly accessible nondynamic peaks set for future comparisons
not.different = rownames(res.lrt[res.lrt$padj > 0.5 & !is.na(res.lrt$padj) &
                                   !is.na(res.lrt$log2FoldChange) & abs(res.lrt$log2FoldChange) < 0.25,])

chr = sapply(strsplit(not.different, ':'), '[', 1)
x = sapply(strsplit(not.different, ':'), '[', 2)
start = sapply(strsplit(x, '-'), '[', 1)
end = sapply(strsplit(x, '-'), '[', 2)

curated.not.different = data.frame(chr,start,end)
write.table(curated.not.different,file='nondynamic_peaks.bed',sep='\t',col.names=F,row.names=F,quote=F)

#generate all peaks set
chr = sapply(strsplit(rownames(res.lrt), ':'), '[', 1)
z = sapply(strsplit(rownames(res.lrt), ':'), '[', 2)
start = sapply(strsplit(z, '-'), '[', 1)
end = sapply(strsplit(z, '-'), '[', 2)

bed = data.frame(chr,start,end,res.lrt$baseMean)
write.table(bed[,1:3], file = 'all_peaks.bed',sep='\t',col.names=F,row.names=F,quote=F)

#plot num. of dynamic peaks

plot.df <- data.frame(type=c('Dynamic','Nondynamic'),
                      num=c(nrow(cluster_rlog),
                            (nrow(res.lrt)-nrow(cluster_rlog))),
                      x=1)

pdf(file='num.dynamic.v.nondynamic.peaks.pdf',height=4,width=4)
print(
  ggplot(plot.df,aes(x = x,y = num,fill=type)) +
    geom_bar(stat='Identity',color='black') +
    labs(title = NULL,
         y = 'Number of Peaks',
         x = NULL,
         fill = 'Type') +
    theme_minimal() +
    theme(panel.grid.minor = element_blank(),
          axis.ticks = element_blank(),
          axis.text.x = element_blank(),
          axis.text.y = element_text(size=16,face='bold',color='black'),
          axis.title.x = element_blank(),
          axis.title.y = element_text(size=18,face='bold'),
          legend.title = element_text(size=16,face='bold'),
          legend.text = element_text(size=14,face='bold'),
          plot.title = element_text(size=17,face='bold',hjust=0.5)) +
    scale_fill_manual(values = c('#f16469','#f1bc7b'))
)
dev.off()
```

Now we cluster dynamic peaks based on shared accessibility dynamics using the DEGreport package. This is a very long, computationally intensive process so we will submit it as a job on the largemem partition. We will submit the 'atac.clustering.slurm' job script which will call the 'atac.clustering.R' script.

atac.clustering.R:

```
library(DESeq2)
library(DEGreport)
library(tibble)
library(lattice)

setwd("/scratch/abd3x/ATAC")

load('cluster_rlog_pval_1e8.Rdata')
```

```
clusters.all.test.1e8 <- degPatterns(cluster_rlog, metadata = meta, minc = 100, time = "sample.conditions",
                                     col=NULL, eachStep = TRUE)

save(clusters.all.test.1e8, file = 'clusters.all.minc100.1e8.Rdata')
```

atac.clustering.slurm:

```
#!/bin/bash
#SBATCH -n 1
#SBATCH -t 96:00:00
#SBATCH -p largemem
#SBATCH -A janeslab
#SBATCH -o atac.clustering.out

module load gcc/9.2.0  openmpi/3.1.6 R/4.2.1

Rscript atac.clustering.R

echo 'DONE'
```

Submit slurm:

```
sbatch atac.clustering.slurm
```

2.7 Post-clustering processing

We save all peaks sorted into clusters into a .bed file. We also save each cluster as its own .bed file and plot a dendrogram to assess cluster similarity. We draw an (arbitrary) lines across the dendrogram to separate the clusters into five 'superclusters' that broadly depict our major classes of accessibility dynamics. The dendrogram can be found in Supplemental Figure S1E.

```
library(lattice)
library(data.table)

load('clusters.all.minc100.1e8.Rdata')

plot.df = clusters.all.test.1e8$normalized

plot.df$sample.conditions <- as.numeric(sapply(strsplit(as.character(plot.df$sample.conditions), 'm'), '[', 1))

plot.df = plot.df[order(plot.df$genes),]
plot.df = plot.df[order(plot.df$sample.conditions),]

plot.df$chr = sapply(strsplit(plot.df$genes, '[.]'), '[', 1)
plot.df$start = sapply(strsplit(plot.df$genes, '[.]'), '[', 2)
plot.df$end = sapply(strsplit(plot.df$genes, '[.]'), '[', 3)

write.table(unique(cbind(plot.df$chr, plot.df$start, plot.df$end)), file = 'dynamic_peaks.bed',
            quote = FALSE, sep = '\t', col.names=FALSE, row.names=FALSE)

#save clusters as .bed files

for (i in unique(plot.df$cluster)) {
  print(i)

  temp <- unique(plot.df[plot.df$cluster == i,c('chr','start','end','cluster')])

  write.table(temp,file = paste0('cluster_',i,'.bed'),
              quote = FALSE, row.names = FALSE, col.names = FALSE, sep = '\t')
}

plot.df$label <- paste0('cluster',plot.df$cluster)

#draw dendrogram

x = as.data.table(plot.df)
plot.df.cluster = dcast(x, genes + label ~ sample.conditions, value.var="value")
avg.clusters = as.data.frame(matrix(nrow = 0, ncol = 7))

for (i in unique(plot.df.cluster$label)) {
  z = data.frame(matrix(colMeans(plot.df.cluster[plot.df.cluster$label == i,3:9]), ncol = 7, nrow = 1))
  rownames(z) = c(i)
  colnames(z) = as.character(colnames(plot.df.cluster)[3:9])
  avg.clusters = rbind(avg.clusters, z)
}

dd = dist(avg.clusters)
hc = hclust(dd, method = "complete")
```

```
pdf('dendrogram.pdf', width=8, height=5)
plot(hc, xlab = "Clusters", main = ' ', hang = -1)
abline(h = 2.1, lty = 2)
dev.off()
```

Next we will plot the traces for each individual cluster as well as each supercluster.

```
#plot clusters organized by supercluster
df = data.frame(index=1:18,cluster.num=
                  unique(plot.df$cluster)[order(unique(as.character(plot.df$cluster)))])
df = df[order(df$cluster.num),]
df = df[reorder(df$cluster.num,c(23,9,18,16,12,4,8,1,11,13,2,5,24,14,10,6,3,7)),]

pdf('atac_clusters_org_by_sc.pdf', width=11, height=15)

trellis.par.set(box.umbrella = list(lty = 1, col="black", lwd=1),
                box.rectangle = list( lwd=1.0, col="black", alpha = 1.0),
                plot.symbol = list(col="black", lwd=1.0, pch ='.'))
print(
  xyplot(value ~  sample.conditions | label, group = genes, data = plot.df, type = c('l'),#type = c('l','p'),
         scales=list(x=list(cex=1.0,relation = "free", rot = 45), y =list(cex=1.0, relation="free")),
         aspect=1.0,
         layout = c(5,5),
         between=list(y=0.5, x=0.5),
         index.cond=list(rev(df$index)),
         skip = c(F,F,F,F,F,
                  F,T,T,T,T,
                  F,F,F,T,T,
                  F,F,F,F,T,
                  F,F,F,F,F),
         ylab = list(label = 'Normalized ATAC signal', cex =1.0),
         xlab = list(label = 'Time (minutes)', cex =1.0),
         par.settings = list(superpose.symbol = list(pch = c(16),
                                                    col=c('grey20'), cex =0.5),
                             strip.background=list(col="grey80"),
                             superpose.line = list(col = c('#99999980'), lwd=c(1),
                                                  lty = c(1))),
         panel = function(x, y, ...) {
           panel.xyplot(x, y, ...)
           panel.bwplot(x, y, pch = '|', horizontal = FALSE, box.width = 15, do.out = FALSE)
           panel.spline(x, y, col = 'blue', lwd =2.0, ...)
         })

)
dev.off()
```

Now we plot the peak traces for each supercluster as one .pdf and as individual files. We also save the peaks in each supercluster as a .bed file. These plots can be found in Figure 1B.

```
plot.df$supercluster <- 'grad.down'
plot.df[plot.df$cluster ==24,'supercluster'] <- 'down.up'
plot.df[plot.df$cluster %in% c(5,2,13),'supercluster'] <- 'up.down'
plot.df[plot.df$cluster %in% c(11,1,8,4),'supercluster'] <- 'grad.up'
plot.df[plot.df$cluster %in% c(12,16,18,9,23),'supercluster'] <- 'up.flat'

#number of peaks in each supercluster
table(plot.df$supercluster) / 7

plot.df.atac = plot.df[,c(1,3,4,6,27:30)]
plot.df.atac$genes = paste0(plot.df.atac$chr,':',plot.df.atac$start,'-',plot.df.atac$end)
colnames(plot.df.atac)[1] <- 'peak'
colnames(plot.df.atac)[3] <- 'time'
save(plot.df.atac,file='plot.df.atac.Rdata')

#plot superclusters
pdf('atac_superclusters.pdf', width=8, height=4)

trellis.par.set(box.umbrella = list(lty = 1, col="black", lwd=1),
                box.rectangle = list( lwd=1.0, col="black", alpha = 1.0),
                plot.symbol = list(col="black", lwd=1.0, pch ='.'))
print(
  xyplot(value ~  time | supercluster, group = peak, data = plot.df.atac, type = c('l'),#type = c('l','p'),
         superclusterales=list(x=list(cex=1.0,relation = "free", rot = 45), y =list(cex=1.0, relation="free")),
         aspect=1.0,
         between=list(y=0.5, x=0.5),
         layout = c(5,1),
         ylab = list(label = 'Normalized ATAC signal', cex =1.0),
         xlab = list(label = 'Time (minutes)', cex =1.0),
         par.settings = list(superpose.symbol = list(pch = c(16),
                                                    col=c('grey20'), cex =0.5),
                             strip.background=list(col="grey80"),
                             superpose.line = list(col = c('#99999980'), lwd=c(1),
                                                  lty = c(1))),
```

```
        panel = function(x, y, ...) {
          panel.xyplot(x, y, ...)
          panel.bwplot(x, y, pch = '|', horizontal = FALSE, box.width = 15, do.out = FALSE)
          panel.spline(x, y, col = 'blue', lwd =2.0, ...)

        })

)
dev.off()

#save individual superclusters as .bed files
for (sc in unique(plot.df.atac$supercluster)) {
  print(sc)

  temp <- unique(plot.df.atac[plot.df.atac$supercluster == sc,c('chr','start','end','supercluster')])

  write.table(temp,file = paste0(sc,'.supercluster.bed'),
              quote = FALSE, row.names = FALSE, col.names = FALSE, sep = '\t')
}

#plot superclusters in individual plots
for(sc in unique(plot.df.atac$supercluster)) {
  print(sc)
  y = plot.df.atac[plot.df.atac$supercluster == sc,]

  pdf(file=paste0(sc,'.supercluster.traces.pdf'),width=3,height=3)

  trellis.par.set(box.umbrella = list(lty = 1, col="black", lwd=1),
                  box.rectangle = list( lwd=1.0, col="black", alpha = 1.0),
                  plot.symbol = list(col="black", lwd=1.0, pch ='.'))

  print(
    xyplot(value ~  time, group = peak, data = y, type = c('l'),
           scales=list(x=list(cex=1.0,relation = "free", rot = 45), y = list(cex=1.0, relation="free")),
           aspect=1.0,
           between=list(y=0.5, x=0.5),
           main = list(label = paste0(sc, ' traces'), cex = 1.5),
           ylab = list(label = 'Normalized ATAC signal', cex =1.0),
           xlab = list(label = 'Time (minutes)', cex =1.0),
           par.settings = list(superpose.symbol = list(pch = c(16),col=c('grey20'), cex =0.5),
                               strip.background=list(col="grey80"),
                               superpose.line = list(col = c('#99999980'), lwd=c(1),lty = c(1))),
           panel = function(x, y, ...) {
             panel.xyplot(x, y, ...)
             panel.bwplot(x, y, pch = '|', horizontal = FALSE, box.width = 15, do.out = FALSE)
             panel.spline(x, y, col = 'blue', lwd = 3.5, ...)
             #replace col = 'blue' with col = col for different sc colors
           })
  )
  dev.off()
}
```

2.8 Identifying enriched TF binding motifs

We use two orthogonal methods to identify TF binding motifs enriched in our dynamic peaks. One is to use assess the percentage of peaks from each cluster that contains any given motif and look for clusters that are significantly more enriched than the nondynamic using a chi squared test.
fimo.motif.enrichment.R

```
Args=commandArgs(TRUE)
motif = Args[1]

library(lattice)

setwd('/scratch/abd3x/ATAC/fimo_motif_enrichment')

supercluster.key = data.frame(row.names = c('cluster_12','cluster_16','cluster_18','cluster_9','cluster_23',
                                            'cluster_11','cluster_1','cluster_8','cluster_4',
                                            'cluster_5','cluster_2','cluster_13',
                                            'cluster_7','cluster_3','cluster_6','cluster_10','cluster_14',
                                            'cluster_24',
                                            'nondynamic'),
                              supercluster = c(rep('up.flat',5),
                                               rep('grad.up',4),
                                               rep('up.down',3),
                                               rep('grad.down',5),
                                               rep('down.up',1),
                                               NA)
)

colors = c('#000000','#dddddd')
```

```
motif.name = strsplit(motif,'.txt')[[1]][1]
print(motif.name)
table = t(read.table(motif,sep='\t',header=T,row.names=1))

result = table
result = result[,c('cluster_12','cluster_16','cluster_18','cluster_9','cluster_23',
                   'cluster_11','cluster_1','cluster_8','cluster_4',
                   'cluster_5','cluster_2','cluster_13',
                   'cluster_7','cluster_3','cluster_6','cluster_10','cluster_14',
                   'cluster_24',
                   'nondynamic')]

sig = FALSE
sig.clusters = c()
for (cluster in colnames(result)) {
  small.table = result[,c(cluster,'nondynamic')]
  output = chisq.test(small.table)
  if (output$p.value < 0.001) {
    change = ''
    if ((small.table[1,1] / small.table[2,1]) > (small.table[1,2]/small.table[2,2])) {
      change = 'enriched'
    } else {
      change = 'depleted'
    }

    sc = as.character(supercluster.key[cluster,])

    write(paste0(motif.name,'\t',cluster,'\t',change,'\t',sc),file="significant_motifs.txt",append=TRUE)
    sig=TRUE
  }
  if (sig == TRUE) {
    pdf(file = paste0(motif.name,'.enrichment.barchart.pdf'),height=9)
    par(las=2)
    barplot(result, col = colors, cex.names= 1.2, legend.text = TRUE,
            args.legend = list(x=2.5,y=113), main = paste0(motif.name,' Enrichment'))
    dev.off()
  }
}
```

fimo_motif_enrichment.slurm

```
#!/bin/bash
#SBATCH -n 1
#SBATCH -t 12:00:00
#SBATCH -o fimo.enrichment.out
#SBATCH -p standard
#SBATCH -A janeslab

module load gcc/9.2.0 bedtools/2.29.2 openmpi/3.1.6 R/4.2.1

#change the directory to wherever you are keeping the ATAC cluster .bed files
cd /scratch/abd3x/ATAC

tab=$'\t'

mkdir fimo_motif_enrichment
cd fimo_motif_enrichment

#loop through each motif
for motif in /scratch/abd3x/ATAC/bed_files//*bed
do
motif_name=$(echo $motif | awk -F"/" '{print $NF}' | awk -F".bed" '{print $1}')
echo $motif_name
touch ${motif_name}.txt
echo "name""$tab""with.motif""$tab""without.motif" >> ${motif_name}.txt

#find how many nondynamic peaks contain the motif
nondyn_with_motif=$(intersectBed -wa -a ../nondynamic_peaks.bed -b $motif | sort -u | wc -l)
nondyn_without_motif=$(intersectBed -v -a ../nondynamic_peaks.bed -b $motif | sort -u | wc -l)

echo "nondynamic$tab$nondyn_with_motif$tab$nondyn_without_motif" >> ${motif_name}.txt

#loop though each cluster
for bed in ../cluster*bed
do
name=$(echo $bed | awk -F"/" '{print $NF}' | awk -F".bed" '{print $1}')
#find how many peaks in each cluster contain the motif
cluster_with_motif=$(intersectBed -wa -a $bed -b $motif | sort -u | wc -l)
cluster_without_motif=$(intersectBed -v -a $bed -b $motif | sort -u | wc -l)

echo "$name$tab$cluster_with_motif$tab$cluster_without_motif" >> ${motif_name}.txt

done

Rscript ../fimo_motif_enrichment.R ${motif_name}.txt
```

```
done

echo 'DONE'
```

The other approach is to perform *de novo* motif extraction on each cluster using MEME. The original script ran MEME in parallel with a different markov_order option but that no longer works on Rivanna for unclear reasons (probably due to MEME updates).

```
wget https://hgdownload-test.gi.ucsc.edu/goldenPath/mm10/bigZips/mm10.chrom.sizes

cd /scratch/abd3x/ATAC
mkdir meme_motif_enrichment

wget https://github.com/guertinlab/adipogenesis/raw/master/Pipeline_ATAC/misc_scripts/meme_header.txt
wget https://github.com/guertinlab/adipogenesis/raw/master/Pipeline_ATAC/misc_scripts/meme_footer.txt

for bed in cluster_*.bed
do
name=$(echo $bed | awk -F".bed" '{print $1}')
echo $name
echo '#SBATCH -o meme.'$name'.out' > temp.txt
echo 'bed='$bed > temp2.txt
cat meme_header.txt temp.txt temp2.txt meme_footer.txt > meme_${name}.slurm
sbatch meme_${name}.slurm
rm temp.txt
rm temp2.txt
done
```

We match all enriched *de novo* motifs to known TF binding motifs using TOMTOM. First we have to download the binding motif databases from JASPAR, Uniprobe, and Homer.

download_databases.sh

```
#Generate homer_uniprobe_jaspar PSWM database

module load gcc/7.1.0  openmpi/3.1.4 python/2.7.16
wget https://raw.githubusercontent.com/guertinlab/adipogenesis/master/Pipeline_ATAC/misc_scripts/HOMER_MEME_conversion.py

wget http://meme-suite.org/meme-software/Databases/motifs/motif_databases.12.19.tgz
tar -xzf motif_databases.12.19.tgz
#JASPAR
mv motif_databases/JASPAR/JASPAR2018_CORE_vertebrates_non-redundant.meme $PWD
#Uniprobe
mv motif_databases/MOUSE/uniprobe_mouse.meme $PWD

#Homer
#CAUTION: the HOMER_MEME_conversion.py was written for Python2 so remember to specify python2.7 when running.
wget https://raw.githubusercontent.com/mjg54/znf143_pro_seq_analysis/master/Pipeline_ATAC/misc_scripts/docs/HOMER_MEME_conversion.py
wget http://homer.ucsd.edu/homer/custom.motifs
python2.7 HOMER_MEME_conversion.py -i custom.motifs -o homer.motifs

#edit databases to work with tomtom
cp JASPAR2018_CORE_vertebrates_non-redundant.meme JASPAR_edited_meme.txt
grep MOTIF JASPAR_edited_meme.txt > motifs.txt
cat motifs.txt | while read motif
do
name=$(echo $motif | awk -F" " '{print $NF}')
temp=$(echo 'MOTIF' $name'_jaspar')
sed -i "s;${motif};${temp};g" JASPAR_edited_meme.txt
done
rm motifs.txt

#edit databases to work with tomtom
cp uniprobe_mouse.meme uniprobe_edited_meme.txt
grep MOTIF uniprobe_edited_meme.txt > motifs.txt
cat motifs.txt | while read motif
do
name=$(echo $motif | awk -F" " '{print $NF}')
temp=$(echo 'MOTIF' $name'_uniprobe')
sed -i "s;${motif};${temp};g" uniprobe_edited_meme.txt
done
#remove 'secondary' motifs
sed -i -e '4210,8365d;' uniprobe_edited_meme.txt

rm motifs.txt

#edit databases to work with tomtom
cp homer.motifs_meme.txt homer_edited_meme.txt
grep MOTIF homer_edited_meme.txt > motifs.txt
cat motifs.txt | while read motif
do
name=$(echo $motif | awk -F" " '{print $NF}')
```

```
name=$(echo $name | awk -F"/" '{print $1}')
temp=$(echo 'MOTIF' $name'_homer')
sed -i "s;${motif};${temp};g" homer_edited_meme.txt
done
rm motifs.txt

#Collect all database motifs into one file
cat homer_edited_meme.txt uniprobe_edited_meme.txt JASPAR_edited_meme.txt > homer_uniprobe_jaspar_edited.txt
```

Now we iterate through the MEME results from each cluster and match the results against the database file.
meme_tomtom.slurm

```
#!/bin/bash
#SBATCH -n 1
#SBATCH -t 12:00:00
#SBATCH -p standard
#SBATCH -A janeslab
#SBATCH -o post.meme.fimo.out

module load gcc/9.2.0  openmpi/3.1.6 meme/5.3.3

#run tomtom on meme output
mkdir tomtom
cd tomtom

echo 'Running TOMTOM'
for i in ../meme_motif_enrichment/*output
do
name=$(echo $i | awk -F"/" '{print $NF}' | awk -F".meme" '{print $1}')
echo $name
tomtom -no-ssc -o $name.tomtom_output -verbosity 1 -min-overlap 5 -dist ed -evalue -thresh 0.05 $i/meme.txt ../homer_uniprobe_jaspar_edited.tx
tomtom -no-ssc -o $name.tomtom_output -verbosity 1 -min-overlap 5 -dist ed -text -evalue -thresh 0.05 $i/meme.txt ../homer_uniprobe_jaspar_edi
done

cd ..

mkdir motifid_clusters
cd motifid_clusters

#extract motif names from tomtom output
echo 'Extracting Motif Names'
for file in ../tomtom/*cluster*tomtom_output/*.txt
do
name=$(echo $file | awk -F"/" '{print $(NF-1)}' | awk -F".tomtom_output" '{print $1}')
echo $name
motifid=$name
#echo $file
linenum=$(awk 'END {print NR}' $file)
#echo $linenum
first=2
i=$first
if [[ $linenum != 5 ]]
then
mkdir $name
while [[  $i -le $linenum ]]
do
head -$i $file | tail -1 > lastline
mapid[$i]=$(awk 'END {print $2}' lastline | awk -F"(" '{print $1}')
#echo mapid[$i]_${mapid[$i]}
echo ${mapid[$i]} >> $name/motifidlist_$motifid.txt
((i = i + 1))
done
head -n $(( $(wc -l $name/motifidlist_$motifid.txt | awk '{print $1}') - 4 )) $name/motifidlist_$motifid.txt > $name/motifidlist_$motifid.fina
rm $name/motifidlist_$motifid.txt
mv $name/motifidlist_$motifid.final.txt $name/motifidlist_$motifid.txt
fi
done

rm lastline
cd ..

echo 'DONE'
```

2.9 Sorting motifs into families

Now we sort the /textitde novo motifs by the supercluster they were extracted from (meaning all 'up.down' motifs get pu together in a directory).
sort_meme_motifs.sh

```
#organize de novo motifs by supercluster
echo 'Sort MEME motifs by supercluster'
mkdir supercluster_meme_motifs
cd supercluster_meme_motifs

mkdir up.down
mkdir up.flat
mkdir grad.up
mkdir down.up
mkdir grad.down

cp -r ../motifid_clusters/cluster_5 up.down
cp -r ../motifid_clusters/cluster_2 up.down
cp -r ../motifid_clusters/cluster_13 up.down

cp -r ../motifid_clusters/cluster_12 up.flat
cp -r ../motifid_clusters/cluster_16 up.flat
cp -r ../motifid_clusters/cluster_18 up.flat
cp -r ../motifid_clusters/cluster_9 up.flat
cp -r ../motifid_clusters/cluster_23 up.flat

cp -r ../motifid_clusters/cluster_11 grad.up
cp -r ../motifid_clusters/cluster_8 grad.up
cp -r ../motifid_clusters/cluster_4 grad.up
cp -r ../motifid_clusters/cluster_1 grad.up

#no motifs tomtom'd out from cluster24 meme result
#cp -r ../motifid_clusters/cluster24 down.up
rm -r down.up

cp -r ../motifid_clusters/cluster_7 grad.down
cp -r ../motifid_clusters/cluster_3 grad.down
cp -r ../motifid_clusters/cluster_6 grad.down
cp -r ../motifid_clusters/cluster_10 grad.down
cp -r ../motifid_clusters/cluster_14 grad.down

for dir in *.*
  do
echo $dir
cd $dir
for int_dir in cluster*
  do
cd $int_dir
mv motifidlist* ..
cd ..
done
cat *txt > $dir.denovo.motifs.txt
mv $dir.denovo.motifs.txt ..
cd ..
done

cd ..
```

Next we sort the motifs enriched from the FIMO analysis by supercluster.

sort_fimo_motifs_supercluster.R

```
#Organize all the fimo identified motifs by supercluster and save them in new directory
setwd('/scratch/abd3x/ATAC')
fimo = read.table('fimo_motif_enrichment/significant_motifs.txt',sep='\t',header=F)

colnames(fimo) = c('motif','cluster','change','supercluster')
enriched = fimo[fimo$change == 'enriched',]

#had to edit the jaspar motif names some during FIMO, changing them back here
enriched$motif = gsub('.var.2.','(var.2)',enriched$motif)
enriched$motif = gsub('\\.\\.','::',enriched$motif)

superclusters = unique(enriched$supercluster)

for (sc in superclusters) {
  print(sc)
  write((as.vector(unique(enriched[enriched$supercluster == sc,]$motif))),
        file=paste0('supercluster_fimo_motifs/',sc,'.fimo.motifs.txt'))
}
```

sort_fimo_motifs.sh

```
module load gcc/9.2.0  openmpi/3.1.6 R/4.2.1

#organize fimo motifs by supercluster
echo 'Sort FIMO motifs by supercluster'
mkdir supercluster_fimo_motifs

Rscript sort_fimo_motifs_supercluster.R
```

Now we identify the overlap between the *de novo* motifs and the FIMO motifs.
fimo_denovo_match.sh

```
echo 'Match MEME and FIMO motifs by supercluster'
mkdir fimo_denovo_match
cd fimo_denovo_match

for file in ../supercluster_meme_motifs/*.txt
do
name=$(echo $file | awk -F"/" '{print $NF}' | awk -F".denovo" '{print $1}')
echo $name
awk 'FNR==NR{a[$1];next}($1 in a){print}' $file ../supercluster_fimo_motifs/$name.fimo.motifs.txt > ${name}_meme_fimo_match.txt
wordcount=$(wc -l ${name}_meme_fimo_match.txt | awk 'END {print $1}')
if [[ $wordcount == 0 ]]
then
rm ${name}_meme_fimo_match.txt
fi
done

cat * > all_matched_motifs.txt
cp all_matched_motifs.txt ..

echo 'DONE'
```

Next we sort all the enriched motifs into a reasonable number of TF families by using TOMTOM to compare every motif against every other motif.
fimo_denovo_sort.R

```
library(igraph)
library(dichromat)

setwd('/scratch/abd3x/ATAC')

threecol=read.table("3_col_combined_motif_db.txt",header=F,stringsAsFactors = F,sep='\t')
colnames(threecol)=c('from','to','e_value')
threecol$weight=abs(log(threecol$e_value))

#create the graph variable
g=graph.data.frame(threecol,directed=F)
g=simplify(g)

cluster=clusters(g)

for(i in 1:length(groups(cluster))) {
  write.table(groups(cluster)[i],file=paste0('PSWM_family_',i,'.txt'),col.names = F, row.names = F, quote = F, sep = '\t')
}

l=layout.fruchterman.reingold(g)
l=layout.norm(l,-1,1,-1,1)

colfunc<-colorRampPalette(c("red","yellow","springgreen","royalblue","purple"))
#pick a distinct color from the palette for each disease and save the list of colors as a vector
mycol = colfunc(length(groups(cluster)))

pdf(file='families.pdf',width=10,height=10)
plot(g,layout=l,rescale=F,vertex.label.cex=.5,xlim=range(l[,1]),  ylim=range(l[,2]),
     edge.width=E(g)$weight/20,vertex.size=degree(g,mode='out')/5,
     edge.curved=T,vertex.label=NA,vertex.color=mycol[cluster$membership],
     margin=0,asp=0)
dev.off()
```

fimo_denovo_sort.sh

```
#pulls each de novo motif from all the clusters and saves each as its own file

#extract individual meme files from combined database
#CAUTION: you only need to run this once, even if you're working through the code again
wget https://raw.githubusercontent.com/guertinlab/adipogenesis/master/Pipeline_ATAC/misc_scripts/MEME_individual_from_db.py

mkdir individual_memes
cd individual_memes

#CAUTION: the MEME_individual_from_db.py was written for Python2 so remember to specify python2.7 when running.
python2.7 ../MEME_individual_from_db.py -i ../homer_uniprobe_jaspar_edited.txt

for file in *meme.txt
do
name=$(echo $file | awk -F"homer_uniprobe_jaspar_edited.txt_" '{print $1}')
mv $file ${name}meme.txt

done

cd ..
```

```
#tomtom all query factors against all others
mkdir tomtom_all_query_factors
cd tomtom_all_query_factors

cat ../all_matched_motifs.txt | while read factor
do
echo $factor
cp ../individual_memes/${factor}_meme.txt $PWD
done

#remove Ptf1a motif b/c it's a forced palindrome
rm Ptf1a_homer_meme.txt
#remove Tal1:Tcf3 motif b/c motif doesn't make biological sense - looks like neither Tal1 nor Tcf3
rm TAL1::TCF3_jaspar_meme.txt
#remove ZNF740 motif
rm ZNF740_jaspar_meme.txt
#remove Pitx1:Ebox b/c it's conflated with TWIST1
rm Pitx1:Ebox_homer_meme.txt

cat *meme.txt > ../all_query_factors_meme.txt

#define motif families
module load gcc/9.2.0  openmpi/3.1.6 meme/5.3.3
for meme in *meme.txt
do
name=$(echo $meme | awk -F".txt_" '{print $NF}' | awk -F"_meme.txt" '{print $1}')
#echo $name
tomtom -no-ssc -o $name.tomtom_output -verbosity 1  -incomplete-scores -min-overlap 1 -dist ed -evalue -thresh 0.0005 $meme ../all_query_facto
cd $name.tomtom_output
cut -f1,2,5 tomtom.tsv | tail -n +2 | sed '$d' | sed '$d' | sed '$d' | sed '$d' >> ../3_col_combined_motif_db_pre.txt
cd ..
done

grep -v '#' 3_col_combined_motif_db_pre.txt > 3_col_combined_motif_db.txt
rm 3_col_combined_motif_db_pre.txt
cp 3_col_combined_motif_db.txt ..
cd ..

module purge
module load gcc/9.2.0  openmpi/3.1.6 R/4.2.1
Rscript fimo_denovo_sort.R
```

Next we generate composite motifs for each family. We used to use ceqlogo from the MEME package to create sequence logos from composite .fasta files, however ceqlogo is no longer part of the package.

```
wget https://raw.githubusercontent.com/guertinlab/adipogenesis/master/Pipeline_ATAC/misc_scripts/tomtom_output_to_composite.py
wget https://raw.githubusercontent.com/guertinlab/adipogenesis/master/Pipeline_ATAC/misc_scripts/meme_header.txt
wget https://raw.githubusercontent.com/guertinlab/adipogenesis/master/Pipeline_ATAC/misc_scripts/generate_composite_motif.R

mkdir composite_motifs
cd composite_motifs

#query tomtom for each factor against all others within each family

for txt in ../PSWM_family*.txt
do

dir_name=$(echo $txt | awk -F'../' '{print $2}' | awk -F'.txt' '{print $1}')
echo $dir_name
mkdir $dir_name
cd $dir_name

cat ../$txt | while read line
do
echo $line
cp ../../individual_memes/${line}_meme.txt $PWD
done
echo ''

query_factor=`head -1 ../$txt`

if [[ $(wc -l < ../$txt) -ge 2 ]]
then
cat ../$txt | { while read line
  do
  query_factor=$line
  rm ref_factors_meme.txt
  mv ${query_factor}_meme.txt ..
  cat *_meme.txt > ref_factors_meme.txt
  mv ../${query_factor}_meme.txt $PWD
  module purge
  module load gcc/9.2.0  openmpi/3.1.6 meme/5.3.3
  tomtom -no-ssc -o ${query_factor}.tomtom_output -verbosity 1 -incomplete-scores \
  -min-overlap 1 -dist ed -evalue -thresh 0.0005 ${query_factor}_meme.txt ref_factors_meme.txt
```

```
  if [[ $(wc -l < ${query_factor}.tomtom_output/tomtom.tsv) -ge $max_motif ]]
  then
  max_motif=$(wc -l < ${query_factor}.tomtom_output/tomtom.tsv)
  final_query=$query_factor
  fi
  done

  echo FINAL_QUERY IS $final_query
  wc -l ${final_query}.tomtom_output/tomtom.tsv
  cd ${final_query}.tomtom_output

  module load gcc/7.1.0  openmpi/3.1.4 python/2.7.16
  python2.7 ../../../tomtom_output_to_composite.py -i tomtom.xml
  mv tomtom.xml_test_index_pswm.txt ../composite.values.txt
  mv tomtom.xml_test_index_rc_offset.txt ../composite.index.txt
  cd ../..
}
fi

cd ..

done

cd ..

module purge
module load gcc/9.2.0  openmpi/3.1.6 R/4.2.1

for family in PSWM*
  do
cd $family
num=$(ls *txt | wc -l)

if [[ $num -ge 2 ]]
then
#generate composite PSWM
Rscript ../../generate_composite_motif.R $family
cat ../../meme_header.txt ${family}_composite_PSWM.txt > ${family}_meme.txt
else
  line=`grep MOTIF *meme.txt`
cp *meme.txt ${family}_meme.txt
sed -i "s;${line};MOTIF   Composite;g" ${family}_meme.txt
fi

#generate logo
#module load gcc/7.1.0 meme/4.10.2
#ceqlogo -i ${family}_meme.txt -m Composite -o ${family}.eps
#ceqlogo -i ${family}_meme.txt -m Composite -o ${family}.rc.eps -r
cd ..

done

cd ..
```

2.10 Separating KLF and SP motif instances

The KLF and SP motifs are very similar and will be sorted into the same family by TOMTOM. However in our dataset, KLF motifs are associated with increases in accessibility and SP motifs are associated with decreases. The following code more confidently distinguishes SP from KLF motifs. These analyses are reported in Supplemental Figure S1I.

```
#downlaod all relevant scripts
wget https://raw.githubusercontent.com/guertinlab/adipogenesis/master/Pipeline_ATAC/misc_scripts/generate_composite_motif.KLF.R
wget https://raw.githubusercontent.com/guertinlab/adipogenesis/master/Pipeline_ATAC/misc_scripts/generate_composite_motif.SP.R
wget https://raw.githubusercontent.com/guertinlab/adipogenesis/master/Pipeline_ATAC/misc_scripts/prep.SP.KLF.fimo.R
wget https://raw.githubusercontent.com/guertinlab/adipogenesis/master/Pipeline_ATAC/misc_scripts/SP_KLF_split.R
wget https://raw.githubusercontent.com/guertinlab/adipogenesis/master/Pipeline_ATAC/misc_scripts/extract.motifs.from.combined.family.R
wget https://raw.githubusercontent.com/guertinlab/adipogenesis/master/Pipeline_ATAC/misc_scripts/post.composite.fimo.supp.R
wget https://raw.githubusercontent.com/guertinlab/adipogenesis/master/Pipeline_ATAC/misc_scripts/plot.motif.enrichment.supp.R

#generate new directory
rm -r SP_KLF_split
mkdir SP_KLF_split
cd SP_KLF_split

echo Generate composite SP and KLF motifs

#separating SP motifs
mkdir SP
cd SP
```

```
motifs=`grep -E 'SP|Sp' ../../PSWM_family_7.txt`

for motif in $motifs
do
cp  /scratch/abd3x/ATAC/individual_memes/$motif*meme.txt $PWD
done

max_motif=0
final_query=''

#query tomtom for each SP factor against all others
for line in *meme.txt
do
name=$(echo $line | awk -F"_meme.txt" '{print $1}')
echo $name
query_factor=$line
mv ${query_factor} ..
rm ref_factors.txt
cat *_meme.txt > ref_factors.txt
mv ../${query_factor} $PWD
#increased e-value threshold to 0.05!

module purge
module load gcc/9.2.0  openmpi/3.1.6 meme/5.3.3

tomtom -no-ssc -oc $name.tomtom_output -verbosity 1 -min-overlap 5 -mi 1 -dist pearson -evalue -thresh 0.05 ${query_factor} ref_factors.txt
if [[ $(wc -l < $name.tomtom_output/tomtom.tsv) -ge $max_motif ]]
then
max_motif=$(wc -l < $name.tomtom_output/tomtom.tsv)
final_query=$name
fi
done

echo FINAL_QUERY IS $final_query
wc -l $final_query.tomtom_output/tomtom.tsv
cd $final_query.tomtom_output

module purge
module load gcc/7.1.0  openmpi/3.1.4 python/2.7.16

python2.7 ../../../tomtom_output_to_composite.py -i tomtom.xml
mv tomtom.xml_test_index_pswm.txt ../composite.values.txt
mv tomtom.xml_test_index_rc_offset.txt ../composite.index.txt
cd ..

module purge
module load gcc/9.2.0  openmpi/3.1.6 R/4.2.1

#generate composite SP PSWM
Rscript ../../generate_composite_motif.SP.R

cat ../../meme_header.txt SP_composite_PSWM.txt > SP_composite_meme.txt

#module load gcc/7.1.0 meme/4.10.2

#generate composite SP logo
#ceqlogo -i SP_composite_meme.txt -m Composite -o SP_composite.eps
#ceqlogo -i SP_composite_meme.txt -m Composite -o SP_composite.rc.eps -r

cd ..

#separating KLF motifs
mkdir KLF
cd KLF

module load gcc/9.2.0  mvapich2/2.3.3 meme/5.1.0

motifs=`grep -E 'KLF|Klf' ../../PSWM_family_7.txt`

for motif in $motifs
do
cp  /scratch/abd3x/ATAC/individual_memes/$motif*meme.txt $PWD
done

max_motif=0
final_query=''

#query tomtom for each KLF factor against all others
for line in *meme.txt
do
name=$(echo $line | awk -F"_meme.txt" '{print $1}')
echo $name
query_factor=$line
mv ${query_factor} ..
rm ref_factors.txt
cat *_meme.txt > ref_factors.txt
```

```
mv ../${query_factor} $PWD
#increased e-value threshold to 0.05!

module purge
module load gcc/9.2.0  openmpi/3.1.6 meme/5.3.3

tomtom -no-ssc -oc $name.tomtom_output -verbosity 1 -min-overlap 5 -mi 1 -dist pearson -evalue -thresh 0.05 ${query_factor} ref_factors.txt
if [[ $(wc -l < $name.tomtom_output/tomtom.tsv) -ge $max_motif ]]
then
max_motif=$(wc -l < $name.tomtom_output/tomtom.tsv)
final_query=$name
fi
done

echo FINAL_QUERY IS $final_query
wc -l $final_query.tomtom_output/tomtom.tsv
cd $final_query.tomtom_output

module purge
module load gcc/7.1.0  openmpi/3.1.4 python/2.7.16

python2.7 ../../../tomtom_output_to_composite.py -i tomtom.xml
mv tomtom.xml_test_index_pswm.txt ../composite.values.txt
mv tomtom.xml_test_index_rc_offset.txt ../composite.index.txt
cd ..

#generate composite KLF PSWM

module purge
module load gcc/9.2.0  openmpi/3.1.6 R/4.2.1

Rscript ../../generate_composite_motif.KLF.R

cat ../../meme_header.txt KLF_composite_PSWM.txt > KLF_composite_meme.txt

#module load gcc/7.1.0 meme/4.10.2

#generate composite KLF logo
#ceqlogo -i KLF_composite_meme.txt -m Composite -o KLF_composite.eps
#ceqlogo -i KLF_composite_meme.txt -m Composite -o KLF_composite.rc.eps -r

cd ..
```

Now we use FIMO to find the top 2 million instances of each motif. As with alignments, we submit a .slurm script for each motif family.
fimo_script.sh

```
rm -r fimo_composites
mkdir fimo_composites

wget https://raw.githubusercontent.com/guertinlab/adipogenesis/master/Pipeline_ATAC/misc_scripts/fimo_footer.txt

for i in PSWM*txt
do
name=$(echo $i | awk -F".txt" '{print $1}')
echo $name
echo '#SBATCH -o' $name'.fimo.out' > temp.txt
echo 'i='../composite_motifs/$name/${name}_meme.txt > temp2.txt
cat header_1.txt temp.txt temp2.txt fimo_footer.txt > $name.fimo.slurm
sbatch $name.fimo.slurm
rm temp.txt
rm temp2.txt
done
```

We rename the FIMO .bed files to be more informative.

```
module load gcc/9.2.0 bedtools/2.29.2

cd /scratch/abd3x/ATAC/fimo_composites

for i in *_2M.txt
do
name=$(echo $i | awk -F"/" '{print $NF}' | awk -F"_2M.txt" '{print $1}')
echo $name
intersectBed -loj -a ../dynamic_peaks.bed -b $i > ${name}_fimo.bed
intersectBed -loj -a ../nondynamic_peaks.bed -b $i > ${name}_fimo_nondyn.bed
intersectBed -loj -a ../all_peaks.bed -b $i > ${name}_fimo_all.bed
cat $i | cut -f1-3,5 | sort -k1,1 -k2,2n > ${name}_2M.bed
done

#transfer bed files for AP1, GR, CEBP, and TWIST into main figures directory
#check that family number matches up to corresponding motif
```

```
rm -r main_figure_beds
mkdir main_figure_beds

cp PSWM_family_1_fimo.bed main_figure_beds/AP1_fimo.bed
cp PSWM_family_3_fimo.bed main_figure_beds/GR_fimo.bed
cp PSWM_family_5_fimo.bed main_figure_beds/CEBP_fimo.bed
cp PSWM_family_18_fimo.bed main_figure_beds/TWIST_fimo.bed

cp PSWM_family_1_2M.bed main_figure_beds/AP1_2M.bed
cp PSWM_family_3_2M.bed main_figure_beds/GR_2M.bed
cp PSWM_family_5_2M.bed main_figure_beds/CEBP_2M.bed
cp PSWM_family_18_2M.bed main_figure_beds/TWIST_2M.bed

#rename 2M files for generating final ATAC dataframe
#skip SP/KLF (family 7)
cp PSWM_family_1_fimo_all.bed BHLH_fimo_all.bed
cp PSWM_family_2_fimo_all.bed TCF21_fimo_all.bed
cp PSWM_family_3_fimo_all.bed GR_fimo_all.bed
cp PSWM_family_4_fimo_all.bed BHLHA15_fimo_all.bed
cp PSWM_family_5_fimo_all.bed CEBP_fimo_all.bed
cp PSWM_family_6_fimo_all.bed CTFCL_fimo_all.bed
#cp PSWM_family_7_fimo_all.bed SPKLF_fimo_all.bed
cp PSWM_family_8_fimo_all.bed ETS_fimo_all.bed
cp PSWM_family_9_fimo_all.bed ZBTB33_fimo_all.bed
cp PSWM_family_10_fimo_all.bed MAZ_fimo_all.bed
cp PSWM_family_11_fimo_all.bed NFIC_fimo_all.bed
cp PSWM_family_12_fimo_all.bed NFY_fimo_all.bed
cp PSWM_family_13_fimo_all.bed NRF_fimo_all.bed
cp PSWM_family_14_fimo_all.bed NUR77_fimo_all.bed
cp PSWM_family_15_fimo_all.bed STAT_fimo_all.bed
cp PSWM_family_16_fimo_all.bed TFAP2A_fimo_all.bed
cp PSWM_family_17_fimo_all.bed TEAD_fimo_all.bed
cp PSWM_family_18_fimo_all.bed TWIST_fimo_all.bed
cp PSWM_family_19_fimo_all.bed ZNF263_fimo_all.bed
```

Last steps to separate SP and KLF motifs.

```
cd /scratch/abd3x/ATAC/SP_KLF_split

module load gcc/9.2.0  openmpi/3.1.6 R/4.2.1 meme/5.3.3 bedtools/2.29.2

echo Starting prep R script

#generate sp_fimo.txt, sp_fimo_nondyn.txt, and sp_klf_2M.txt
#caution: it is suggested to run this Rscript manually to verify slope and intercept of dataset; may vary depending on input
Rscript ../prep.SP.KLF.fimo.R

echo Starting SP FIMO
fimo --thresh 0.01 --text SP/SP_composite_meme.txt sp_fimo.txt > output_sp1.txt
echo Starting KLF FIMO
fimo --thresh 0.01 --text KLF/KLF_composite_meme.txt sp_fimo.txt > output_klf.txt

#Query top 2 million FIMO hits of SP/KLF against their composite meme
cp /scratch/abd3x/ATAC/fimo_composites/PSWM_family_7_2M.bed $PWD/sp_klf_2M.bed
bedtools getfasta -fi /project/genomes/Mus_musculus/UCSC/mm10/Sequence/WholeGenomeFasta/genome.fa -bed sp_klf_2M.bed > sp_klf_2M.fasta

fimo --thresh 0.01 --text SP/SP_composite_meme.txt sp_klf_2M.txt > output_sp1_2M.txt
fimo --thresh 0.01 --text KLF/KLF_composite_meme.txt sp_klf_2M.txt > output_klf_2M.txt

echo Starting split R script

#Generate SP_unsorted.bed and KLF_unsorted.bed
Rscript ../SP_KLF_split.R
Rscript ../extract.motifs.from.combined.family.R

echo DONE
```

2.11 Create ATAC data frame

Now we create a data frame that contains all the relevant information for our ATAC peaks including accessibility over the time course, motif presence, and pairwise accessibility comparisons. Since the computationally intensive steps are complete we can move to the local system, although one can continue working on the cluster if preferred. The final step of adding distributions (e.g. promoter v. intergenic v. intragenic) can only be done after primary transcript annotation of the PRO-seq data.

```
library(DESeq2)

categorize.deseq.df <- function(df, fdr = 0.05, log2fold = 0.0, treat
                                = 'Auxin') {
```

```
  df.effects.lattice = df
  df.effects.lattice$response = 'All Other Peaks'

  if(nrow(df.effects.lattice[df.effects.lattice$padj < fdr & !is.na(df.effects.lattice$padj) & df.effects.lattice$log2FoldChange > log2fold,])
    df.effects.lattice[df.effects.lattice$padj < fdr & !is.na(df.effects.lattice$padj) & df.effects.lattice$log2FoldChange > log2fold,]$respon
  }
  if(nrow(df.effects.lattice[df.effects.lattice$padj < fdr & !is.na(df.effects.lattice$padj) & df.effects.lattice$log2FoldChange < log2fold,])
    df.effects.lattice[df.effects.lattice$padj < fdr & !is.na(df.effects.lattice$padj) & df.effects.lattice$log2FoldChange < log2fold,]$respon
  }
  if(nrow(df.effects.lattice[df.effects.lattice$padj > 0.5 & !is.na(df.effects.lattice$padj) & abs(df.effects.lattice$log2FoldChange) < 0.25,]
    df.effects.lattice[df.effects.lattice$padj > 0.5 & !is.na(df.effects.lattice$padj) & abs(df.effects.lattice$log2FoldChange) < 0.25,]$respo
  }

  return(df.effects.lattice)
}

setwd("/Users/guertinlab/Adipogenesis/ATAC_analysis_redo")

peaks = unique(read.table('all_peaks.bed',sep='\t'))

peaks$location = paste0(peaks$V1,':',peaks$V2,'-',peaks$V3)

df = data.frame(row.names = peaks$location,chr=peaks[,1],start=peaks[,2],end=peaks[,3])

#add counts
load('normalized.counts.atac.Rdata')

zero.min = c()
twenty.min = c()
forty.min = c()
sixty.min = c()
onetwenty.min = c()
oneeighty.min = c()
twoforty.min = c()
genes = c()

print('Getting ATAC means')
for (i in 1:nrow(normalized.counts.atac)) {
  zero.min = append(zero.min, mean(normalized.counts.atac[i,19:21]))
  twenty.min = append(twenty.min, mean(normalized.counts.atac[i,1:3]))
  forty.min = append(forty.min, mean(normalized.counts.atac[i,10:12]))
  sixty.min = append(sixty.min, mean(normalized.counts.atac[i,16:18]))
  onetwenty.min = append(onetwenty.min,mean(normalized.counts.atac[i,4:6]))
  oneeighty.min = append(oneeighty.min, mean(normalized.counts.atac[i,7:9]))
  twoforty.min = append(twoforty.min, mean(normalized.counts.atac[i,13:15]))
  genes = append(genes,rownames(normalized.counts.atac)[i])
}

atac.means = data.frame(row.names = genes, min.0 = zero.min, min.20 = twenty.min,
                        min.40 = forty.min, min.60 = sixty.min, min.120 = onetwenty.min,
                        min.180 = oneeighty.min, min.240 = twoforty.min)

save(atac.means,file='atac.means.Rdata')

df = merge(df,atac.means,by='row.names',all=TRUE)
rownames(df) = df[,1]
df = df[,-1]

#add cluster information
print('Adding Cluster Info')
print(head(df))

supercluster.df = data.frame(cluster=c(12,16,18,9,23,11,8,4,1,5,2,13,7,3,6,10,14,24),
                             supercluster=c(rep('up.flat',5),rep('grad.up',4),
                                            rep('up.down',3),rep('grad.down',5),rep('down.up',1)))

cluster.df = data.frame()
for (file in Sys.glob(file.path(paste0('cluster_bed_cluster*.bed')))) {
  cluster = as.numeric(strsplit(strsplit(file,'cluster_bed_cluster')[[1]][2],'.bed')[[1]][1])
  print(cluster)
  sc = supercluster.df[supercluster.df$cluster == cluster,]$supercluster
  x = read.table(file)
  x$location = paste0(x$V1,':',x$V2,'-',x$V3)
  cluster.df = rbind(cluster.df,data.frame(peak=x$location,cluster=cluster,supercluster=sc))
}

df = merge(df,cluster.df,by.x='row.names',by.y=1,all.x=TRUE)
rownames(df) = df[,1]
df = df[,-1]

#add TF family scores
print('Adding TF Family Scores')
print(head(df))

scores.df = data.frame()
for (file in Sys.glob('fimo_composites/*_fimo_all.bed')) {
```

```
  factor = strsplit(strsplit(file,'/')[[1]][2],'_fimo_all.bed')[[1]][1]
  print(factor)
  x = read.table(file,sep='\t')
  x = x[x$V5 != -1,]
  if(factor %in% c('SP','KLF')) {
    y = aggregate(as.numeric(V7)~V1+V2+V3, data=x, FUN=sum)
  } else {
    y = aggregate(as.numeric(V8)~V1+V2+V3, data=x, FUN=sum)
  }
  colnames(y) = c('chr', 'start', 'end', factor)
  rownames(y) = paste0(y[,1], ':', y[,2], '-', y[,3])

  scores.df = rbind(scores.df,data.frame(peak = rownames(y),score=y[,4],factor = factor))
}

fimo.scores.all.atac = data.frame(peak = unique(scores.df$peak))

for(factor in unique(scores.df$factor)) {
  temp = scores.df[scores.df$factor == factor,]
  temp = temp[,c(1,2)]
  fimo.scores.all.atac = merge(fimo.scores.all.atac,temp,by='peak',all.x=TRUE)
  colnames(fimo.scores.all.atac)[ncol(fimo.scores.all.atac)] = factor
}

rownames(fimo.scores.all.atac) = fimo.scores.all.atac$peak
fimo.scores.all.atac = fimo.scores.all.atac[,-1]
save(fimo.scores.all.atac,file='fimo.scores.all.atac.Rdata')

df = merge(df,fimo.scores.all.atac,by = 'row.names',all = TRUE)
rownames(df) = df$Row.names
df = df[,-1]

#add pairwise comparisons
print('Adding Pairwise Comparisons')
print(head(df))

load('df.preadipo.Rdata')

time.pts.key = data.frame(time.pts=c(rep(20,3),rep(120,3),rep(180,3),rep(40,3),rep(240,3),rep(60,3),rep(0,3)),
                          cols = c(1:21))

time.pts = c(0,20,40,60,120,180,240)

comparisons.df = data.frame()
for (i in 1:(length(time.pts)-1)) {
  for (j in (i+1):length(time.pts)) {

    print(paste0(time.pts[j],'.v.',time.pts[i]))

    a = time.pts.key[time.pts.key$time.pts == time.pts[i],]$cols
    b = time.pts.key[time.pts.key$time.pts == time.pts[j],]$cols
    merged.counts.small = df.preadipo[,c(a,b)]

    # number of replicates per condition
    unt = 3
    trt = 3

    sample.conditions = factor(c(rep("untreated",unt), rep("treated",trt)), levels=c("untreated","treated"))
    mm.deseq.counts.table = DESeqDataSetFromMatrix(merged.counts.small, DataFrame(sample.conditions), ~ sample.conditions)

    mm.atac = mm.deseq.counts.table
    atac.size.factors = estimateSizeFactorsForMatrix(merged.counts.small)

    sizeFactors(mm.atac) = atac.size.factors
    mm.atac = estimateDispersions(mm.atac)
    mm.atac = nbinomWaldTest(mm.atac)
    res.mm.atac = results(mm.atac)

    lattice = categorize.deseq.df(res.mm.atac, fdr = 0.001, log2fold = 0.0, treat = '')
    lattice = as.data.frame(lattice[,c(2,6,7)])
    colnames(lattice) = paste0(colnames(lattice),'.',time.pts[j],'.v.',time.pts[i])

    comparisons.df = merge(comparisons.df,lattice,by='row.names',all=TRUE)
    rownames(comparisons.df) = comparisons.df$Row.names
    comparisons.df = comparisons.df[,-1]
  }
}

save(comparisons.df,file='comparisons.df.Rdata')

df = merge(df,comparisons.df,by = 'row.names',all = TRUE)
rownames(df) = df$Row.names
df = df[,-1]

print('Add baseMean and overall time course info')
```

```
load('res.lrt.Rdata')
res.lrt = as.data.frame(res.lrt[,c(1,2,6)])
res.lrt$response = 'Nondynamic'
res.lrt[res.lrt$padj < 0.00000001 & !is.na(res.lrt$padj),]$response = 'Dynamic'
colnames(res.lrt) = paste0(colnames(res.lrt),'.time.course')

df = merge(df,res.lrt,by = 'row.names',all = TRUE)
rownames(df) = paste0(df$chr,':',df$start,'-',df$end)
df = df[,-1]

#The following can be done after primary transcript annotation
#print('Add distribution')

#df$distribution = 'Intergenic'

#x = read.table('all_ATAC_peaks_intragenic.bed')
#x$location = paste0(x$V1,':',x$V2,'-',x$V3)
#df[rownames(df) %in% x$location,]$distribution = 'Intragenic'

#x = read.table('all_ATAC_peaks_promoters.bed')
#x$location = paste0(x$V1,':',x$V2,'-',x$V3)
#df[rownames(df) %in% x$location,]$distribution = 'Promoter'

final.atac.all.df=df
save(final.atac.all.df,file='final.atac.all.df.Rdata')
```

2.12 Plotting motif density around ATAC peak summits

To plot motif density around accessibility peaks we first convert our peak .bed files to .bigWigs using UCSC tools. Then we use the bigWig package to load the files into R and plot the composite density around the summits of different classes of ATAC-seq peaks (stored as .bed files). This approach can be taken to plot the enrichment of any continuous signal stored as a .bigWig (e.g. ATAC, ChIP, evolutionary conservation) at regions of interest stored in a .bed file (e.g. peaks, TSSs, motifs). These analyses are found in Figure 1E and Supplemental Figure S1H.

```
cd fimo_composites/main_figure_beds/

  for bed in *2M.bed
do
name=$(echo $bed | awk -F"/" '{print $NF}' | awk -F"_2M.bed" '{print $1}')
echo $name
#summing scores of motifs w/in peak
cat $bed | mergeBed -i stdin -c 4 -o sum > ${name}_merged_2M.bed
bedGraphToBigWig ${name}_merged_2M.bed ../../mm10.chrom.sizes ${name}_mm10_instances.bigWig
done
```

```
library(lattice)
library(bigWig)

bed.window <- function(bed, half.window) {
  bed[,2] = (bed[,2] + bed[,3])/2 - half.window
  bed[,3] = bed[,2] + 2 * half.window
  #This shouldn't be necessary, but I used it as a workaround while troubleshooting
  bed = subset(bed, bed[,2] > 0)
  return(bed)
}

plot.fimo.lattice <- function(dat, fact = 'Motif', summit = 'Hypersensitivity Summit', class= '',
                              num.m = -200, num.p =90, y.low =0, y.high = 0.2,
                              col.lines = c(rgb(0,0,1,1/2), rgb(1,0,0,1/2),
                                            rgb(0.1,0.5,0.05,1/2), rgb(0,0,0,1/2),
                                            rgb(1/2,0,1/2,1/2), rgb(0,1/2,1/2,1/2), rgb(1/2,1/2,0,1/2)),
                              fill.poly = c(rgb(0,0,1,1/4), rgb(1,0,0,1/4), rgb(0.1,0.5,0.05,1/4),
                                            rgb(0,0,0,1/4), rgb(1/2,0,1/2,1/4))) {

  pdf('motif_enrichment_around_summits.pdf')#, width=6.83, height=3.5)
  print(xyplot(density ~ range|tf, groups = category, data = dat, type = 'l',
               scales=list(x=list(cex=0.8,relation = "free"), y =list(cex=0.8,axs = 'i',relation = "free")),
               xlim=c(num.m,num.p),
               col = col.lines,
               auto.key = list(points=F, lines=T, cex=0.8, columns = 2),
               par.settings = list(superpose.symbol = list(pch = c(16), col=col.lines, cex =0.7),
                                   superpose.line = list(col = col.lines, lwd=c(2,2,2,2,2,2),
                                                         lty = c(1,1,1,1,1,1,1,1,1))),
               cex.axis=1.0,
               par.strip.text=list(cex=0.9, font=1, col='black',font=2),
               aspect=1.0,
               between=list(y=0.5, x=0.5),
               index.cond = list(c(4:6,1:3)),
```

```
                lwd=2,
                ylab = list(label = "Weighted Motif Density", cex =1,font=2),
                xlab = list(label = 'Distance from ATAC-seq Peak Summit', cex =1,font=2),
                strip = function(..., which.panel, bg) {
                  bg.col = 'grey'#c("blue","grey65","red")
                  strip.default(..., which.panel = which.panel, bg = rep(bg.col, length = which.panel)[which.panel])
                }
  ))
  dev.off()
}

load('plot.df.atac.Rdata')
load('fimo.scores.all.atac.Rdata')
fimo.scores.atac <- fimo.scores.all.atac

plot.df = data.frame()
for(i in 1:ncol(fimo.scores.atac)) {
  temp = plot.df.atac[plot.df.atac$genes %in% rownames(fimo.scores.atac[!is.na(fimo.scores.atac[,i]),]),]
  temp$family = colnames(fimo.scores.atac)[i]
  plot.df = rbind(plot.df,temp)
}

plot.df$status = 'Activated'
plot.df[plot.df$supercluster == 'gradual.down' | plot.df$supercluster == 'down.up',]$status = 'Repressed'

all.fimo = data.frame(matrix(ncol = 4, nrow = 0))
colnames(all.fimo) = c('density', 'tf', 'category', 'range')

half.win = 600
file.suffix = '_mm10_instances.bigWig'
dir = 'fimo_composites/main_figure_beds/'

decreased = plot.df[plot.df$status == 'Repressed',7:9]
decreased[,2] = as.numeric(decreased[,2])
decreased[,3] = as.numeric(decreased[,3])
decreased = bed.window(decreased,half.win)

increased = plot.df[plot.df$status == 'Activated',7:9]
increased[,2] = as.numeric(increased[,2])
increased[,3] = as.numeric(increased[,3])
increased = bed.window(increased,half.win)

not.different = read.table('nondynamic_peaks.bed')
not.different = not.different[not.different$V1 != 'chrM',]
not.different = bed.window(not.different,half.win)

all.fimo = data.frame()

for(i in 1:ncol(fimo.scores.atac)) {
  factor = colnames(fimo.scores.atac)[i]

  mod.bigWig = paste0(dir,factor,file.suffix)
  factor.name = factor
  print(factor.name)

  loaded.bw = load.bigWig(mod.bigWig)

  dec.inten = bed.step.probeQuery.bigWig(loaded.bw, decreased,
                                           gap.value = 0, step = 10, as.matrix = TRUE)
  dec.query.df = data.frame(cbind(colMeans(dec.inten), factor.name,
                                  'Closed', seq(-half.win, (half.win-10), 10)), stringsAsFactors=F)
  colnames(dec.query.df) = c('density', 'tf', 'category', 'range')

  inc.inten = bed.step.probeQuery.bigWig(loaded.bw, increased,
                                           gap.value = 0, step = 10, as.matrix = TRUE)
  inc.query.df = data.frame(cbind(colMeans(inc.inten), factor.name,
                                  'Opened', seq(-half.win,(half.win-10), 10)), stringsAsFactors=F)
  colnames(inc.query.df) = c('density', 'tf', 'category', 'range')

  ctrl.inten = bed.step.probeQuery.bigWig(loaded.bw, not.different,
                                            gap.value = 0, step = 10, as.matrix = TRUE)
  ctrl.query.df = data.frame(cbind(colMeans(ctrl.inten), factor.name,
                                   'Nondynamic', seq(-half.win, (half.win-10), 10)), stringsAsFactors=F)
  colnames(ctrl.query.df) = c('density', 'tf', 'category', 'range')

  tf.all = rbind(dec.query.df, inc.query.df, ctrl.query.df)

  all.fimo = rbind(all.fimo,tf.all)
}

all.fimo[,1] = as.numeric(all.fimo[,1])
all.fimo[,4] = as.numeric(all.fimo[,4])

plot.fimo.lattice(all.fimo, num.m = -500, num.p = 500,
                  col.lines = c('blue','grey65','red'))
```

3 PRO-seq analysis

3.1 Download, align, and process data files

Much of this analysis is derived from the primary transcript annotation documentation as described here: `https://github.com/WarrenDavidAnderson/genomicsRpackage/tree/master/primaryTranscriptAnnotation.` We download and align PRO-seq data same as ATAC-seq. First we download the .fastq files as parallel jobs.

```
mkdir /scratch/abd3x/PRO
cd /scratch/abd3x/PRO

wget https://raw.githubusercontent.com/guertinlab/adipogenesis/master/Pipeline_ATAC/misc_scripts/PRO_SRA_table.txt
wget https://raw.githubusercontent.com/guertinlab/adipogenesis/master/Pipeline_ATAC/misc_scripts/header.txt

echo 'Downloading .fastq'

cat PRO_SRA_table.txt | while read line
do
sra=$(echo $line | awk '{print $1}')
time=$(echo $line | awk '{print $2}')
rep=$(echo $line | awk '{print $3}')
echo '#SBATCH -o download.'$time'm.rep'$rep'.out' > temp.txt
echo 'module load sratoolkit/2.10.5' > temp2.txt
echo 'fasterq-dump '$sra' -o PRO_'$time'm_rep'${rep}.fastq > temp3.txt
echo 'echo DONE' > temp4.txt
cat header.txt temp.txt temp2.txt temp3.txt temp4.txt > download.${time}m.rep$rep.slurm
sbatch download.${time}m.rep$rep.slurm
rm temp.txt
rm temp2.txt
rm temp3.txt
rm temp4.txt
done
```

We need several pieces of software to successfully align and process the PRO-seq data.

```
#install ucsc packages via conda env (conda activate myenv2)
#use 'conda activate myenv2' to access packages
module load bioconda/py3.8
conda create -n myenv2 -c bioconda ucsc-bedgraphtobigwig ucsc-bigwigmerge

#install packages for alignment and put on $PATH
#cutadapt
python3 -m pip install --user --upgrade cutadapt
cp /home/abd3x/.local/bin/cutadapt /home/abd3x/bin

#fastx-toolkit (need fastx_trimmer, fastx_reverse_complement)
mkdir fastx_bin
cd fastx_bin
wget http://hannonlab.cshl.edu/fastx_toolkit/fastx_toolkit_0.0.13_binaries_Linux_2.6_amd64.tar.bz2
tar -xjf fastx_toolkit_0.0.13_binaries_Linux_2.6_amd64.tar.bz2
cp bin/* /home/abd3x/bin/
  cd..

#fqdedup
#install rust
curl --proto '=https' --tlsv1.2 -sSf https://sh.rustup.rs | sh
#restart console so cargo is added to $PATH automatically
git clone https://github.com/guertinlab/fqdedup.git
cd fqdedup
cargo build --release
cp /scratch/abd3x/PRO/fqdedup/target/release/fqdedup /home/abd3x/bin
```

Now we will use a script to submit a job to align each sample.
align_footer.txt

```
name=$(echo $i | awk -F"/" '{print $NF}' | awk -F"fastq" '{print $1}')
echo $name

module load  bioconda/py3.8 gcc/9.2.0 bowtie2/2.2.9 samtools/1.12
source activate myenv2

echo 'trim adapters'
cutadapt -m 26 -a TGGAATTCTCGGGTGCCAAGG $i | \
fqdedup -i - -o - | \
fastx_trimmer -Q33 -f 9 -l 38 | \
fastx_reverse_complement -Q33 -z -o ${name}.processed.fastq.gz
```

```
echo 'align to mouse genome'
bowtie2 -p 3 -x /project/genomes/Mus_musculus/UCSC/mm10/Sequence/Bowtie2Index/genome -U ${name}.processed.fastq.gz | \
samtools view -b - | \
samtools sort - -o ${name}.sorted.bam

echo 'sort and separate plus/minus reads'
samtools view -bh -F 20 ${name}.sorted.bam > ${name}_pro_plus.bam
samtools view -bh -f 0x10 ${name}.sorted.bam > ${name}_pro_minus.bam

echo 'DONE'
```

```
for i in *fastq
do
name=$(echo $i | awk -F"/" '{print $NF}' | awk -F".fastq" '{print $1}')
echo $name
echo '#SBATCH -o' $name'.align.out' > temp.txt
echo 'i='$i > temp2.txt
cat header.txt temp.txt temp2.txt align_footer.txt > $name.align.slurm
sbatch $name.align.slurm
rm temp.txt
rm temp2.txt
done
```

Now we convert the output to .bigWig files.
bigwig_footer.txt:

```
echo 'convert to bigwig'
seqOutBias /project/genomes/Mus_musculus/UCSC/mm10/Sequence/WholeGenomeFasta/genome.fa ${name}_pro_plus.bam --no-scale --skip-bed \
--bw=${name}_plus_body_0-mer.bigWig --tail-edge --read-size=30
seqOutBias /project/genomes/Mus_musculus/UCSC/mm10/Sequence/WholeGenomeFasta/genome.fa ${name}_pro_minus.bam --no-scale --skip-bed \
--bw=${name}_minus_body_0-mer.bigWig --tail-edge --read-size=30
```

bigwig_script.sh:

```
for bam in *_pro_plus.bam
do
name=$(echo $bam | awk -F"_pro_plus.bam" '{print $1}')
echo $name
echo '#SBATCH -o' $name'.bigwig.out' > temp.txt
echo 'name='$name > temp2.txt
cat header.txt temp.txt temp2.txt bigwig_footer.txt > $name.bigwig.slurm
rm temp.txt
rm temp2.txt
done

#caution: you can't use the tallymer mappability file from ATAC because it has a different read size!
#run one slurm file to completion to generate requisite tallymer mappability file
sbatch PRO_0m_rep1.bigwig.slurm

#after this is done, start all others (and repeat the first one)
for slurm in *bigwig*slurm
do
sbatch $slurm
done
```

3.2 Primary transcript annotation

We perform primary transcript annotation to define transcription start and end sites for each gene.
primary_transcript_annotation.R:

```
library(devtools)
library(NMF)
library(dplyr)
library(bigWig)
library(pracma)
library(RColorBrewer)
#install_github("WarrenDavidAnderson/genomicsRpackage/primaryTranscriptAnnotation")
library(primaryTranscriptAnnotation)

setwd('/scratch/abd3x/PRO/primary_transcript_annotation')

source('pTA.functions.R')

# import data for first exons, annotate, and remove duplicate transcripts
fname = "../gencode.mm10.firstExon.bed"
dat0 = read.table(fname,header=F,stringsAsFactors=F)
names(dat0) = c('chr', 'start', 'end', 'gene', 'xy', 'strand')
```

```
dat0 = unique(dat0)
gencode.firstExon = dat0
# import data for all transcripts, annotate, and remove duplicate transcripts
fname = "../gencode.mm10.transcript.bed"
dat0 = read.table(fname,header=F,stringsAsFactors=F)
names(dat0) = c('chr', 'start', 'end', 'gene', 'xy', 'strand')
dat0 = unique(dat0)
gencode.transcript = dat0
# chromosome sizes
chrom.sizes = read.table("../mm10.chrom.sizes",stringsAsFactors=F,header=F)
names(chrom.sizes) = c("chr","size")

plus.file = "../pro_plus_merged.bigWig"
minus.file = "../pro_minus_merged.bigWig"
bw.plus = load.bigWig(plus.file)
bw.minus = load.bigWig(minus.file)

#get intervals for furthest TSS and TTS +/- interval
largest.interval.bed = get.largest.interval(bed=gencode.transcript)
# filtering which read count and read density might be consider as insignificant
transcript.reads = read.count.transcript(bed=gencode.transcript, bw.plus=bw.plus, bw.minus=bw.minus)

# evaluate count and density distributions
pdf("Histogram_density_distibution.pdf", useDingbats = FALSE, width=6.4, height=5.4)
par(mfrow=c(1,2))
hist(log(transcript.reads$density), breaks=200,
     col="black",xlab="log10 read density",main="")
hist(log(transcript.reads$counts), breaks=200,
     col="black",xlab="log10 read count",main="")
dev.off()

# specify which genes to cut based on low expression, visualize cutoffs
pdf("Histogram_density_distibution_treshold.pdf", useDingbats = FALSE, width=6.4, height=5.4)
den.cut = -4
cnt.cut = 2
ind.cut.den = which(log(transcript.reads$density) < den.cut)
ind.cut.cnt = which(log(transcript.reads$counts) < cnt.cut)
ind.cut = union(ind.cut.den, ind.cut.cnt)
par(mfrow=c(1,2))
hist(log(transcript.reads$density), breaks=200,
     col="black",xlab="log"[10]~" read density",main="")
abline(v=den.cut, col="red")
hist(log(transcript.reads$counts), breaks=200,
     col="black",xlab="log"[10]~" read count",main="")
abline(v=cnt.cut, col="red")
dev.off()

# remove "unexpressed" genes
unexp = names(transcript.reads$counts)[ind.cut]
largest.interval.expr.bed = largest.interval.bed[
  !(largest.interval.bed$gene %in% unexp),]
# select the TSS for each gene and incorporate these TSSs
# into the largest interval coordinates
bp.range = c(20,120)
cnt.thresh = 2
bed.out = largest.interval.expr.bed
bed.in = gencode.firstExon[gencode.firstExon$gene %in% bed.out$gene,]
TSS.gene = get.TSS(bed.in=bed.in, bed.out=bed.out,
                   bw.plus=bw.plus, bw.minus=bw.minus,
                   bp.range=bp.range, cnt.thresh=cnt.thresh)
TSS.gene = TSS.gene$bed

# parameters and analysis
window = 1000
bp.bin = 10
bed1 = TSS.gene
bed2 = largest.interval.expr.bed
bed2 = bed2[bed2$gene %in% bed1$gene,]
tss.eval = eval.tss(bed1=bed1, bed2=bed2,
                    bw.plus=bw.plus, bw.minus=bw.minus,
                    window=window, bp.bin=bp.bin, fname="TSSres.pdf")

#Inferred TSSs showed as distribution of paused RNA polymerase density
pdf("TS.pdf", useDingbats = FALSE, width=6.4, height=5.4)
bk = seq(-window/2,window/2, bp.bin)
hist(tss.eval$tss.dists.inf$dist, main="overlay",
     xlab="dist from TSS to read max (bp)",
     breaks=bk, col='black')
bk = seq(-window/2,window/2, bp.bin)
hist( tss.eval$tss.dists.lng$dist,
      breaks=bk, col='red', add=T)
dev.off()

# identify duplicates
dups = get.dups(bed = TSS.gene)
max(dups$cases)
```

```
head(dups)
write.table(dups,"duplicateCoords.bed",quote=F,sep="\t",col.names=F,row.names=F)

#put *merged.bigWig files on cyverse and look at duplicate examples in browser
#annotations that should be entirely removed go into 'duplicate_remove.csv'
#annotations that should be kept should be ordered and put into 'duplicate_keepadjacent.csv'
remove.genes.id <- read.csv(file="duplicate_remove.csv",
                            header=TRUE, sep=",", stringsAsFactors=F)
fix.genes.id <- read.csv("duplicate_keepadjacent.csv",
                         header= TRUE, sep=",", stringsAsFactors=F)
# remove genes based on manual analysis, genes cosidered as adjecent will be adressed below
genes.remove1 = c(remove.genes.id$remove, fix.genes.id$upstream, fix.genes.id$downstream)
TSS.gene.filtered1 = TSS.gene[!(TSS.gene$gene %in% genes.remove1),]
# confirm that there are no any overlaps
#this object should have no data
dups1 =get.dups(bed = TSS.gene.filtered1)

# overlap analysis
overlap.data = gene.overlaps(bed = TSS.gene.filtered1)
has.start.inside = overlap.data$has.start.inside
is.a.start.inside = overlap.data$is.a.start.inside
head(has.start.inside)
dim(has.start.inside) #271
head(is.a.start.inside)
dim(is.a.start.inside) #323
head(overlap.data$cases)
dim(overlap.data$cases) #503

# Checking those genes in overlap.gene lists
overlap.genes = overlap.data$cases$gene %>% unique
length(grep("^AC[0-9][0-9]",overlap.genes)) #7
length(grep("^AL[0-9][0-9]",overlap.genes)) #3
length(grep("^AP[0-9][0-9]",overlap.genes)) #0
# set to remove usually poorly annotated genes in overlapping regions
genes.remove2 = overlap.genes[grep("^AC[0-9][0-9]",overlap.genes)]
genes.remove2 = c(genes.remove2, overlap.genes[grep("^AL[0-9][0-9]",overlap.genes)])
genes.remove2 = c(genes.remove2, overlap.genes[grep("^AP[0-9][0-9]",overlap.genes)])
# identify genes with multiple starts inside (i.e. 'big' genes)
mult.inside.starts = inside.starts(vec = is.a.start.inside$xy)
length(mult.inside.starts) #31
# add genes with multiple starts inside to remove
genes.remove2 = c(genes.remove2, mult.inside.starts %>% unique) #41
# remove filtered genes and re-run overlap analysis
in.dat = TSS.gene.filtered1[!(TSS.gene.filtered1$gene %in% genes.remove2),]
overlap.data = gene.overlaps( bed = in.dat )
has.start.inside = overlap.data$has.start.inside
is.a.start.inside = overlap.data$is.a.start.inside
case.dat = overlap.data$cases
length(unique(case.dat$cases)) #171
# data for manual analysis and curration
fname = "nonid_overlaps.txt"
write.table(case.dat,fname,col.names=T,row.names=F,sep="\t",quote=F)
overlaps = case.dat %>% select(chr,start,end,gene,cases)
write.table(overlaps,"nonid_overlaps.bed",quote=F,sep="\t",col.names=F,row.names=F)

write.table(TSS.gene.filtered1,"TSS.gene.filtered1.bed",quote=F,sep="\t",col.names=F,row.names=F)

remove.genes.ov = read.csv("overlaps_remove.csv",
                           header=TRUE, stringsAsFactors=F)
fix.genes.ov =read.csv("overlaps_keepadjacent.csv",
                       header=TRUE, stringsAsFactors=F)
genes.remove3 = c(genes.remove2, remove.genes.ov$remove,
                  fix.genes.ov$upstream, fix.genes.ov$downstream)
# read corrected start/end for 10 genes in bed file

TSS.gene.filtered2 = TSS.gene.filtered1[
  !(TSS.gene.filtered1$gene %in% genes.remove3),]
# verify the abscence of overlaps
overlap.data = gene.overlaps( bed = TSS.gene.filtered2 )
overlap.data$cases

fix.genes.ov =read.csv("overlaps_keepadjacent.csv",
                       header=TRUE, stringsAsFactors=F)
# get the coordinates for adjacent gene pairs
fix.genes = rbind(fix.genes.id, fix.genes.ov)
bp.bin = 5
knot.div = 40
shift.up = 100
delta.tss = 50
diff.tss = 1000
dist.from.start = 50
adjacent.coords = adjacent.gene.coords(fix.genes=fix.genes, bed.long=TSS.gene,
                                       exon1=gencode.firstExon,
                                       bw.plus=bw.plus, bw.minus=bw.minus,
                                       knot.div=knot.div, bp.bin=bp.bin,
                                       shift.up=shift.up, delta.tss=delta.tss,
```

```
                                        dist.from.start=dist.from.start,
                                        diff.tss=diff.tss, fname="adjacentSplines.pdf")

# visualize coordinates for a specific pair
adjacent.coords.plot(adjacent.coords=adjacent.coords,
                     pair=fix.genes[1,],
                     bw.plus=bw.plus,
                     bw.minus=bw.minus)

# aggregate downstream adjacent genes with main data
TSS.gene.filtered3 = rbind(TSS.gene.filtered2, adjacent.coords)
overlap.data = gene.overlaps( bed = TSS.gene.filtered3 )
overlap.data$cases

#get intervals for TTS evaluation
add.to.end = 100000
fraction.end = 0.2
dist.from.start = 50
bed.for.tts.eval = get.end.intervals(bed=TSS.gene.filtered3,
                                     add.to.end=add.to.end,
                                     fraction.end=fraction.end,
                                     dist.from.start=dist.from.start)
# distribution of clip distances
pdf("Histogram_TTS_clipdistance.pdf", useDingbats = FALSE, width=6.4, height=5.4)
hist(bed.for.tts.eval$xy, xlab="clip distance (bp)",col="black",main="")
dev.off()

# identify gene ends
add.to.end = max(bed.for.tts.eval$xy)
knot.div = 40
pk.thresh = 0.05
bp.bin = 50
knot.thresh = 5
cnt.thresh = 5
tau.dist = 50000
frac.max = 1
frac.min = 0.3
inferred.coords = get.TTS(bed=bed.for.tts.eval, tss=TSS.gene.filtered3,
                          bw.plus=bw.plus, bw.minus=bw.minus,
                          bp.bin=bp.bin, add.to.end=add.to.end,
                          pk.thresh=pk.thresh, knot.thresh=knot.thresh,
                          cnt.thresh=cnt.thresh, tau.dist=tau.dist,
                          frac.max=frac.max, frac.min=frac.min, knot.div=knot.div)

coords = inferred.coords$bed
# check for the percentage of identified TTSs that match the search region boundry
TTS.boundary.match(coords=coords, bed.for.tts.eval=bed.for.tts.eval) #0.1968006
# look at whether TTSs identified at the search boundry had clipped boundries
frac.bound.clip = TTS.boundary.clip(coords=coords, bed.for.tts.eval=bed.for.tts.eval)
frac.bound.clip$frac.clip #0.919607
frac.bound.clip$frac.noclip #0.08039303
# plot didtribution of clip distances for genes with boundary TTSs
pdf("bp_clipped_for_boundry.pdf", useDingbats = FALSE, width=6.4, height=5.4)
hist(frac.bound.clip$clip.tts,main="",col="black",
     xlab="bp clipped for boundry genes")
dev.off()

# plot new coord id
gene.end = bed.for.tts.eval
# ploting NR3C1
long.gene = largest.interval.bed
pdf("Genenr3c1.pdf", onefile=FALSE)
tts.plot(coords=coords, gene.end=gene.end, long.gene=long.gene,
         gene = "Nr3c1", xper=0.1, yper=0.2,
         bw.plus=bw.plus, bw.minus=bw.minus, bp.bin=5,
         frac.min=frac.min, frac.max=frac.max,
         add.to.end=add.to.end, tau.dist=tau.dist)
dev.off()

# plot tts curve
pdf("Gene_curvenr3c1.pdf", onefile=FALSE)
gene.end.plot(bed=bed.for.tts.eval, gene="Nr3c1",
              bw.plus=bw.plus, bw.minus=bw.minus,
              bp.bin=bp.bin, add.to.end=add.to.end, knot.div=knot.div,
              pk.thresh=pk.thresh, knot.thresh=knot.thresh,
              cnt.thresh=cnt.thresh, tau.dist=tau.dist,
              frac.max=frac.max, frac.min=frac.min)
dev.off()

write.table(coords,'primary_transcript_annotation.bed',sep='\t',quote=F,col.names=F,row.names=F)
```

```
cd /scratch/abd3x/primary_transcript_annotation
wget https://raw.githubusercontent.com/guertinlab/adipogenesis/master/Pipeline_PRO/misc_scripts/pTA.functions.R
wget https://raw.githubusercontent.com/guertinlab/adipogenesis/master/Pipeline_PRO/misc_scripts/overlaps_remove.csv
wget https://raw.githubusercontent.com/guertinlab/adipogenesis/master/Pipeline_PRO/misc_scripts/overlaps_keepadjacent.csv
```

```
wget https://raw.githubusercontent.com/guertinlab/adipogenesis/master/Pipeline_PRO/misc_scripts/duplicate_remove.csv
wget https://raw.githubusercontent.com/guertinlab/adipogenesis/master/Pipeline_PRO/misc_scripts/duplicate_keepadjacent.csv

#remove DOS \r\n\ artifact from .csv
sed -i 's/\r$//' *csv

#!/bin/bash

module load bioconda/py3.8 ucsc-tools/3.7.4 gcc/9.2.0  openmpi/3.1.6 R/4.2.1

cd /scratch/abd3x/PRO

# should not run wget in slurm--download connection is often severed
wget https://hgdownload-test.gi.ucsc.edu/goldenPath/mm10/bigZips/mm10.chrom.sizes
wget ftp://ftp.ebi.ac.uk/pub/databases/gencode/Gencode_mouse/release_M25/gencode.vM25.annotation.gtf.gz
gunzip gencode.vM25.annotation.gtf.gz

# get the first exons for protein coding genes
grep 'transcript_type "protein_coding"' gencode.vM25.annotation.gtf | \
awk '{if($3=="exon"){print $0}} ' | \
grep -w "exon_number 1" | \
cut -f1,4,5,7,9 | tr ";" "\t" | \
awk '{for(i=5;i<=NF;i++){if($i~/^gene_name/){a=$(i+1)}} print $1,$2,$3,a,"na",$4}' | \
tr " " "\t" | tr -d '"' > gencode.mm10.firstExon.bed

# get all transcripts for protein coding genes
grep 'transcript_type "protein_coding"' gencode.vM25.annotation.gtf | \
awk '{if($3=="transcript"){print $0}} ' | \
cut -f1,4,5,7,9 | tr ";" "\t" | \
awk '{for(i=5;i<=NF;i++){if($i~/^gene_name/){a=$(i+1)}} print $1,$2,$3,a,"na",$4}' | \
tr " " "\t" | tr -d '"' > gencode.mm10.transcript.bed

# chrom sizes file
chromsizes=mm10.chrom.sizes
# merge plus
plusfiles=$(ls *plus*bigWig)
bigWigMerge ${plusfiles} pro_plus_merged.bedGraph
LC_COLLATE=C sort -k1,1 -k2,2n pro_plus_merged.bedGraph > pro_plus_merged_sorted.bedGraph
bedGraphToBigWig pro_plus_merged_sorted.bedGraph ${chromsizes} pro_plus_merged.bigWig
# merge minus
minusfiles=$(ls *minus*bigWig)
bigWigMerge ${minusfiles} pro_minus_merged.bedGraph
LC_COLLATE=C sort -k1,1 -k2,2n pro_minus_merged.bedGraph > pro_minus_merged_sorted.bedGraph
bedGraphToBigWig pro_minus_merged_sorted.bedGraph ${chromsizes} pro_minus_merged.bigWig

# this pTA Rscript takes 1.5hr to compile--run using slurm for convenience
Rscript primary_transcript_annotation.R
```

3.3 Calling dynamic genes

We use DESeq2 to normalize read counts and define genes that are dynamic over the time course as with the ATAC-seq peaks.

```
library(bigWig)
library(DESeq2)
library(DEGreport)
library(tibble)
library(lattice)
library(tidyr)
source('https://raw.githubusercontent.com/mjg54/znf143_pro_seq_analysis/master/docs/ZNF143_functions.R')

dir = '/scratch/abd3x/PRO/'

setwd(dir)

gene.file = read.table(paste0(dir,'primary_transcript_annotation/primary_transcript_annotation.bed'), header=FALSE)

get.raw.counts.interval.pro <- function(df, path.to.bigWig, file.prefix = 'H', file.suffix = '.bigWig') {
  vec.names = c()
  inten.df=data.frame(matrix(ncol = 0, nrow = nrow(df)))

  for (mod.bigWig in Sys.glob(file.path(path.to.bigWig, paste0(file.prefix, "*plus", file.suffix)))) {
    print(mod.bigWig)
    factor.name = strsplit(strsplit(mod.bigWig, "/")[[1]][length(strsplit(mod.bigWig, "/")[[1]])], '_plus')[[1]][1]
    print(factor.name)
    vec.names = c(vec.names, factor.name)
    loaded.bw.plus = load.bigWig(mod.bigWig)
    loaded.bw.minus = load.bigWig(paste0(path.to.bigWig,'/',factor.name,'_minus',file.suffix))
    mod.inten = bed6.region.bpQuery.bigWig(loaded.bw.plus, loaded.bw.minus, df)
    inten.df = cbind(inten.df, mod.inten)
  }
  colnames(inten.df) = vec.names
```

```
  r.names = paste(df[,1], ':', df[,2], '-', df[,3],'_', df[,4], sep='')
  row.names(inten.df) = r.names
  return(inten.df)
}

df.3t3 = get.raw.counts.interval.pro(gene.file, dir, file.prefix = '3T3',file.suffix = '_body_0-mer.bigWig')

colnames(df.3t3) = sapply(strsplit(colnames(df.3t3), '3T3_'), '[', 2)

save(df.3t3, file = 'df.3t3.Rdata')

#for normalizing bigWigs for browser
write.table(estimateSizeFactorsForMatrix(df.3t3),file = 'norm.bedGraph.sizeFactor.txt', quote =F, col.names=F)

sample.conditions = factor(sapply(strsplit(as.character(colnames(df.3t3)), '_'), '[', 1))

sample.conditions = factor(sample.conditions, levels=c("t0","20min","40min","60min","2hr","3hr","4hr"))

deseq.counts.table = DESeqDataSetFromMatrix(df.3t3, as.data.frame(sample.conditions), ~ sample.conditions)

dds = DESeq(deseq.counts.table)

#counts table
normalized.counts.pro = counts(dds, normalized=TRUE)
save(normalized.counts.pro,file='normalized.counts.pro.Rdata')

#PCA
rld = rlog(dds, blind=TRUE)

#I think two ATAC-seq samples were labeled incorrectly. Contacted GEO, confirmed with an old email from Warren.
x = plotPCA(rld, intgroup="sample.conditions", returnData=TRUE)
plotPCAlattice(x, file = 'PCA_pro.pdf')

#clustering
dds.lrt = DESeq(dds, test="LRT", reduced = ~ 1)

res.lrt = results(dds.lrt)
save(res.lrt, file = 'res.lrt.Rdata')

padj.cutoff = 1e-40

siglrt.re = res.lrt[res.lrt$padj < padj.cutoff & !is.na(res.lrt$padj),]

rld_mat <- assay(rld)
cluster_rlog = rld_mat[rownames(siglrt.re),]
meta = as.data.frame(sample.conditions)
rownames(meta) = colnames(cluster_rlog)
save(cluster_rlog, meta, sample.conditions, file = 'cluster_rlog_pval_1e40.Rdata')

#define dynamic genes here rather than after clustering b/c ~600 genes not sorted into clusters
a = strsplit(rownames(cluster_rlog),'_')
gene = unlist(a)[2*(1:nrow(cluster_rlog))]
a = unlist(a)[2*(1:nrow(cluster_rlog))-1]
a = strsplit(a,':')
chr = unlist(a)[2*(1:nrow(cluster_rlog))-1]
a = unlist(a)[2*(1:nrow(cluster_rlog))]
a = strsplit(a,'-')
start = unlist(a)[2*(1:nrow(cluster_rlog))-1]
end = unlist(a)[2*(1:nrow(cluster_rlog))]

bed = data.frame(chr=chr,start=start,end=end,gene=gene)

write.table(bed, file = 'dynamic_genes.bed', quote = FALSE, sep = '\t', col.names=FALSE, row.names=FALSE)
```

We submit the clustering script as a SLURM job because it takes a long time.
pro_clustering.R

```
library(DESeq2)
library(DEGreport)
library(tibble)
library(lattice)

setwd('/scratch/abd3x/PRO')

load('cluster_rlog_pval_1e40.Rdata')

clusters.all.test.1e40 <- degPatterns(cluster_rlog, metadata = meta, minc = 100, time = "sample.conditions", col=NULL, eachStep = TRUE)

save(clusters.all.test.1e40, file = 'clusters.all.minc100.1e40.Rdata')
```

pro_script.sh

```
#!/bin/bash
#SBATCH -n 1
```

```
#SBATCH -t 96:00:00
#SBATCH -p largemem
#SBATCH -A janeslab
#SBATCH -o pro.clustering.out

module load gcc/9.2.0  openmpi/3.1.6 R/4.2.1

Rscript pro_clustering.R

echo 'DONE'
```

After clustering we plot the traces of all the clusters. The PRO-seq signal is too dynamic for clusters to be very informative.

```
library(lattice)
library(data.table)

setwd('/scratch/abd3x/PRO')

source('/scratch/abd3x/ATAC/plot.traces.R')

load('clusters.all.minc100.1e40.Rdata')

#generate 'plot.df' object
plot.df = clusters.all.test.1e40$normalized

plot.df$sample.conditions = as.character(plot.df$sample.conditions)
plot.df$sample.conditions[plot.df$sample.conditions == 't0'] = 0
plot.df$sample.conditions[plot.df$sample.conditions == '20min'] = 20
plot.df$sample.conditions[plot.df$sample.conditions == '40min'] = 40
plot.df$sample.conditions[plot.df$sample.conditions == '60min'] = 60
plot.df$sample.conditions[plot.df$sample.conditions == '2hr'] = 120
plot.df$sample.conditions[plot.df$sample.conditions == '3hr'] = 180
plot.df$sample.conditions[plot.df$sample.conditions == '4hr'] = 240
plot.df$sample.conditions = as.numeric(plot.df$sample.conditions)
plot.df = plot.df[order(plot.df$genes),]
plot.df = plot.df[order(plot.df$sample.conditions),]

plot.df$cluster = paste('cluster', as.character(plot.df$cluster), sep = '')

plot.df$chr = sapply(strsplit(plot.df$genes, '[.]'), '[', 1)
plot.df$start = sapply(strsplit(plot.df$genes, '[.]'), '[', 2)
plot.df$end = sapply(strsplit(sapply(strsplit(plot.df$genes, '[.]'), '[', 3),'_'), '[', 1)
plot.df$gene = sapply(strsplit(plot.df$genes, '_'), '[', 2)

save(plot.df,file='plot.df.Rdata')

#plot all clusters
for (i in unique(plot.df$cluster)) {
  print(i)
  write.table(plot.df[plot.df$cluster == i,
                      c('chr','start','end', 'value', 'cluster')][!duplicated(plot.df[plot.df$cluster == i,]$genes),],
              file = paste0('cluster_bed_',
                            gsub(" ", "", i, fixed = TRUE),'.bed'),
              quote = FALSE, row.names = FALSE, col.names = FALSE, sep = '\t')
}

pdf('pro_clusters.pdf', width=11, height=15)

trellis.par.set(box.umbrella = list(lty = 1, col="black", lwd=1),
                box.rectangle = list( lwd=1.0, col="black", alpha = 1.0),
                plot.symbol = list(col="black", lwd=1.0, pch ='.'))
print(
  xyplot(value ~  sample.conditions | cluster, group = genes, data = plot.df, type = c('l'),#type = c('l','p'),
         scales=list(x=list(cex=1.0,relation = "free", rot = 45), y =list(cex=1.0, relation="free")),
         aspect=1.0,
         between=list(y=0.5, x=0.5),
         ylab = list(label = 'Normalized PRO signal', cex =1.0),
         xlab = list(label = 'Time (minutes)', cex =1.0),
         par.settings = list(superpose.symbol = list(pch = c(16),
                                                     col=c('grey20'), cex =0.5),
                             strip.background=list(col="grey80"),
                             superpose.line = list(col = c('#99999980'), lwd=c(1),
                                                   lty = c(1))),
         panel = function(x, y, ...) {
           panel.xyplot(x, y, ...)
           panel.bwplot(x, y, pch = '|', horizontal = FALSE, box.width = 15, do.out = FALSE)
           panel.loess(x, y, ..., col = "blue", lwd =2.0,  span = 1/2, degree = 1, family = c("gaussian"))

         })

)
dev.off()

#dendrogram
```

```
x = as.data.table(plot.df)
plot.df.cluster = dcast(x, genes + cluster ~ sample.conditions, value.var="value")

avg.clusters = as.data.frame(matrix(nrow = 0, ncol = 7))
for (i in unique(plot.df.cluster$cluster)) {
  z = data.frame(matrix(colMeans(plot.df.cluster[plot.df.cluster$cluster == i,3:9]), ncol = 7, nrow = 1))
  rownames(z) = c(i)
  colnames(z) = as.character(colnames(plot.df.cluster)[3:9])
  avg.clusters = rbind(avg.clusters, z)
}

dd = dist(avg.clusters)
hc = hclust(dd, method = "complete")

pdf('dendrogram.pdf', width=8, height=5)
plot(hc, xlab = "Clusters", main = ' ', hang = -1)
abline(h = 2, lty = 2)
dev.off()

#save plotting object
#there are ~600 dynamic genes that are NOT in the plotting object b/c they are not sorted into clusters
plot.df.pTA = plot.df[,c(1:6,27:30)]
save(plot.df.pTA,file='plot.df.pTA.Rdata')

plot.traces(unique(plot.df.pTA$gene),'All Dynamic Genes')
```

Now we put all PRO-seq information into a single data frame.

```
library(DESeq2)

categorize.deseq.df <- function(df, fdr = 0.05, log2fold = 0.0, treat
                                 = 'Auxin') {

  df.effects.lattice = df
  df.effects.lattice$response = 'All Other Genes'

  if(nrow(df.effects.lattice[df.effects.lattice$padj < fdr & !is.na(df.effects.lattice$padj)
                             & df.effects.lattice$log2FoldChange > log2fold,]) > 0) {
    df.effects.lattice[df.effects.lattice$padj < fdr & !is.na(df.effects.lattice$padj)
                       & df.effects.lattice$log2FoldChange > log2fold,]$response = 'Activated'
  }
  if(nrow(df.effects.lattice[df.effects.lattice$padj < fdr & !is.na(df.effects.lattice$padj)
                             & df.effects.lattice$log2FoldChange < log2fold,]) > 0) {
    df.effects.lattice[df.effects.lattice$padj < fdr & !is.na(df.effects.lattice$padj)
                       & df.effects.lattice$log2FoldChange < log2fold,]$response = 'Repressed'
  }
  if(nrow(df.effects.lattice[df.effects.lattice$padj > 0.5 & !is.na(df.effects.lattice$padj)
                             & abs(df.effects.lattice$log2FoldChange) < 0.25,]) > 0) {
    df.effects.lattice[df.effects.lattice$padj > 0.5 & !is.na(df.effects.lattice$padj)
                       & abs(df.effects.lattice$log2FoldChange) < 0.25,]$response = 'Unchanged'
  }

  return(df.effects.lattice)
}

setwd("/Users/guertinlab/Adipogenesis/PRO_analysis_redo")

#dynamic.genes = read.table('dynamic_genes.bed',sep='\t')

pTA = read.table('primary_transcript_annotation/primary_transcript_annotation.bed')

df = data.frame(matrix(nrow=nrow(pTA),ncol=0))

rownames(df) = paste0(pTA[,1],':',pTA[,2],'-',pTA[,3],'_',pTA[,4])

df$chr = pTA[,1]
df$start = pTA[,2]
df$end = pTA[,3]
df$gene = pTA[,4]
df$strand = pTA[,6]

df$location = paste0(df$chr,':',df$start,'-',df$end)

#add TSS
func <- function(gene) {
  if(df[df$gene == gene,]$strand == '+') {
    return(df[df$gene == gene,]$start)
  } else {
    return(df[df$gene == gene,]$end)
  }
}

df$TSS = sapply(df$gene,func)

#add size
df$size = abs(df$start-df$end)
```

```
#add counts
load('normalized.counts.pro.Rdata')

zero.min = c()
twenty.min = c()
forty.min = c()
sixty.min = c()
onetwenty.min = c()
oneeighty.min = c()
twoforty.min = c()
genes = c()

print('Getting PRO means')
for (i in 1:nrow(normalized.counts.pro)) {
  zero.min = append(zero.min, mean(normalized.counts.pro[i,19:21]))
  twenty.min = append(twenty.min, mean(normalized.counts.pro[i,1:3]))
  forty.min = append(forty.min, mean(normalized.counts.pro[i,10:12]))
  sixty.min = append(sixty.min, mean(normalized.counts.pro[i,16:18]))
  onetwenty.min = append(onetwenty.min,mean(normalized.counts.pro[i,4:6]))
  oneeighty.min = append(oneeighty.min, mean(normalized.counts.pro[i,7:9]))
  twoforty.min = append(twoforty.min, mean(normalized.counts.pro[i,13:15]))
  genes = append(genes,rownames(normalized.counts.pro)[i])
}

pro.means = data.frame(row.names = genes, min.0 = zero.min, min.20 = twenty.min,
                       min.40 = forty.min, min.60 = sixty.min, min.120 = onetwenty.min,
                       min.180 = oneeighty.min, min.240 = twoforty.min)

save(pro.means,file='pro.means.Rdata')

df = merge(df,pro.means,by='row.names',all=TRUE)
rownames(df) = df[,1]
df = df[,-1]

#add pairwise comparisons
print('Adding Pairwise Comparisons')

load('df.3t3.Rdata')

time.pts.key = data.frame(time.pts=c(rep(20,3),rep(120,3),rep(180,3),rep(40,3),rep(240,3),rep(60,3),rep(0,3)),
                          cols = c(1:21))

time.pts = c(0,20,40,60,120,180,240)

comparisons.df = data.frame(row.names=rownames(df))
for (i in 1:(length(time.pts)-1)) {
  for (j in (i+1):length(time.pts)) {

    print(paste0(time.pts[j],'.v.',time.pts[i]))

    a = time.pts.key[time.pts.key$time.pts == time.pts[i],]$cols
    b = time.pts.key[time.pts.key$time.pts == time.pts[j],]$cols
    merged.counts.small = df.3t3[,c(a,b)]

    # number of replicates per condition
    unt = 3
    trt = 3

    sample.conditions = factor(c(rep("untreated",unt), rep("treated",trt)), levels=c("untreated","treated"))
    mm.deseq.counts.table = DESeqDataSetFromMatrix(merged.counts.small, DataFrame(sample.conditions), ~ sample.conditions)

    mm.atac = mm.deseq.counts.table
    atac.size.factors = estimateSizeFactorsForMatrix(merged.counts.small)

    sizeFactors(mm.atac) = atac.size.factors
    mm.atac = estimateDispersions(mm.atac)
    mm.atac = nbinomWaldTest(mm.atac)
    res.mm.atac = results(mm.atac)

    lattice = categorize.deseq.df(res.mm.atac, fdr = 0.001, log2fold = 0.0, treat = '')
    lattice = as.data.frame(lattice[,c(2,6,7)])
    colnames(lattice) = paste0(colnames(lattice),'.',time.pts[j],'.v.',time.pts[i])

    comparisons.df = merge(comparisons.df,lattice,by='row.names',all=TRUE)
    rownames(comparisons.df) = comparisons.df$Row.names
    comparisons.df = comparisons.df[,-1]
  }
}

save(comparisons.df,file='comparisons.df.Rdata')

df = merge(df,comparisons.df,by = 'row.names',all = TRUE)
rownames(df) = df$Row.names
df = df[,-1]

print('Add baseMean and overall time course info')
```

```
load('res.lrt.Rdata')
res.lrt = as.data.frame(res.lrt[,c(1,2,6)])
res.lrt$response = 'Nondynamic'
res.lrt[res.lrt$padj < 1e-40 & !is.na(res.lrt$padj),]$response = 'Dynamic'
colnames(res.lrt) = paste0(colnames(res.lrt),'.time.course')

df = merge(df,res.lrt,by = 'row.names',all = TRUE)
rownames(df) = df$Row.names
df = df[,-1]

final.pro.all.df=df
save(final.pro.all.df,file='final.pro.all.df.Rdata')
```

3.4 Normalizing PRO-seq data for browser

We merge and normalize all .bigWig files from each condition for visualization on the browser. Like with alignment, we will submit each condition as a separate job on Rivanna. The resulting 'normalized.bigWig' files can be uploaded to the browser.
bw_convert_footer.txt

```
plus_1=${name}_rep1_plus*.bigWig
minus_1=${name}_rep1_minus*.bigWig
plus_2=${name}_rep2_plus*.bigWig
minus_2=${name}_rep2_minus*.bigWig
plus_3=${name}_rep3_plus*.bigWig
minus_3=${name}_rep3_minus*.bigWig
Rscript normalization_factor.R $plus_1 $minus_1 $plus_2 $minus_2 $plus_3 $minus_3 $name
reads=`awk '{SUM+=$2}END{print SUM}' ${name}_normalization.txt`
norm=$(echo 10000000/$reads | bc -l | xargs printf "%.*f\n" 3)
echo $norm
bigWigMerge $plus_1 $plus_2 $plus_3  ${name}_plus_merged.bedGraph
sort -k1,1 -k2,2n ${name}_plus_merged.bedGraph > ${name}_plus_merged_sorted.bedGraph
python normalize_bedGraph.py -i ${name}_plus_merged_sorted.bedGraph -n ${norm} -o ${name}_plus_merged_scaled.bedGraph
bedGraphToBigWig ${name}_plus_merged_scaled.bedGraph mm10.chrom.sizes ${name}_plus_merged_normalized.bigWig
bigWigMerge $minus_1  $minus_2 $minus_3  ${name}_minus_merged.bedGraph
sort -k1,1 -k2,2n ${name}_minus_merged.bedGraph > ${name}_minus_merged_sorted.bedGraph
python normalize_bedGraph.py -i ${name}_minus_merged_sorted.bedGraph -n ${norm} -o ${name}_minus_merged_scaled.bedGraph
bedGraphToBigWig ${name}_minus_merged_scaled.bedGraph mm10.chrom.sizes ${name}_minus_merged_normalized.bigWig

echo 'DONE'
```

bw_convert_script.sh

```
wget https://raw.githubusercontent.com/guertinlab/adipogenesis/master/Pipeline_PRO/misc_scripts/normalization_factor.R
wget https://raw.githubusercontent.com/guertinlab/adipogenesis/master/Pipeline_PRO/misc_scripts/normalize_bedGraph.py

for bw in  *_rep1_plus_body_0-mer.bigWig
do
name=$(echo $bw | awk -F"/" '{print $NF}' | awk -F"_rep1_plus_body_0-mer.bigWig" '{print $1}')
echo $name
echo '#SBATCH -o' $name'.bw.conversion.out' > temp.txt
echo 'name='$name > temp2.txt
cat header.txt temp.txt temp2.txt bw_convert_footer.txt > $name.bw.convert.slurm
sbatch $name.bw.convert.slurm
rm temp.txt
rm temp2.txt
done
```

4 Integrating accessibility and transcription

4.1 Transcription of genes only near increased or decreased peaks

First identify all dynamic genes within 10 kilobases of a dynamic peak. We will need this for network inference later.

```
slopBed -i dynamic_genes.bed -g mm10.chrom.sizes -b 10000 > dynamic_genes_plus10kb.bed

intersectBed -wa -wb -a dynamic_peaks.bed \
-b dynamic_genes_plus10kb.bed > all_dynamic_genes_near_peaks.bed
```

Now we will identify genes that are only near peaks that either increase or decrease and plot their change in transcription over the first hour. This analysis shows that peaks near multiple increased

peaks are more likely to be activated, and vice versa. Being near a single increased or decreased peak does not significantly affect the likelihood of activation v. repression. This is Figure 3A.

```
library(ggplot2)
library(ggnewscale)

load('final.atac.all.df.Rdata')
load('plot.df.atac.Rdata')
load('final.pro.all.df.Rdata')
load('plot.df.pTA.Rdata')

x = read.table('all_dynamic_genes_near_peaks.bed')
x$ATAC.peak = paste0(x$V1,':',x$V2,'-',x$V3)

final.atac.all.df$early = 'Unchanged'

final.atac.all.df[rownames(final.atac.all.df) %in% rownames(
  unique(rbind(final.atac.all.df[final.atac.all.df[,21] == 'Decreased',],
               final.atac.all.df[final.atac.all.df[,24] == 'Decreased',],
               final.atac.all.df[final.atac.all.df[,27] == 'Decreased',],
               final.atac.all.df[final.atac.all.df[,39] == 'Decreased',],
               final.atac.all.df[final.atac.all.df[,42] == 'Decreased',],
               final.atac.all.df[final.atac.all.df[,54] == 'Decreased',]
  ))),]$early = 'Down'

final.atac.all.df[rownames(final.atac.all.df) %in% rownames(
  unique(rbind(final.atac.all.df[final.atac.all.df[,21] == 'Increased',],
               final.atac.all.df[final.atac.all.df[,24] == 'Increased',],
               final.atac.all.df[final.atac.all.df[,27] == 'Increased',],
               final.atac.all.df[final.atac.all.df[,39] == 'Increased',],
               final.atac.all.df[final.atac.all.df[,42] == 'Increased',],
               final.atac.all.df[final.atac.all.df[,54] == 'Increased',]
  ))),]$early = 'Up'

final.pro.all.df$early = 'Unchanged'

final.pro.all.df[rownames(final.pro.all.df) %in% rownames(
  unique(rbind(final.pro.all.df[final.pro.all.df[,18] == 'Repressed',],
               final.pro.all.df[final.pro.all.df[,21] == 'Repressed',],
               final.pro.all.df[final.pro.all.df[,24] == 'Repressed',],
               final.pro.all.df[final.pro.all.df[,36] == 'Repressed',],
               final.pro.all.df[final.pro.all.df[,39] == 'Repressed',],
               final.pro.all.df[final.pro.all.df[,51] == 'Repressed',]
  ))),]$early = 'Down'

final.pro.all.df[rownames(final.pro.all.df) %in% rownames(
  unique(rbind(final.pro.all.df[final.pro.all.df[,18] == 'Activated',],
               final.pro.all.df[final.pro.all.df[,21] == 'Activated',],
               final.pro.all.df[final.pro.all.df[,24] == 'Activated',],
               final.pro.all.df[final.pro.all.df[,36] == 'Activated',],
               final.pro.all.df[final.pro.all.df[,39] == 'Activated',],
               final.pro.all.df[final.pro.all.df[,51] == 'Activated',]
  ))),]$early = 'Up'

func <- function(peak) {
  return(final.atac.all.df[rownames(final.atac.all.df) == peak,]$early)
}

x$atac.early = sapply(x$ATAC.peak,func)

func <- function(gene) {
  return(final.pro.all.df[final.pro.all.df$gene == gene,]$early)
}

x$pro.early = sapply(x$V7,func)
temp = x
save(temp,file='temp.Rdata')

z = data.frame()
for(gene in unique(x$V7)) {
  temp = x[x$V7 == gene,]
  if(!('Unchanged' %in% temp$atac.early) & length(unique(temp$atac.early)) == 1) {
    if(unique(temp$atac.early)[1] == 'Up') {
      temp$dir = 'Increased'
    } else if (unique(temp$atac.early)[1] == 'Down') {
      temp$dir = 'Decreased'
    }
    z = rbind(z,temp)
  }
}

genes = c()
num = c()
dir = c()
pro.changes = c()
atac.changes = c()
```

```
for(gene in unique(z$V7)) {
  if(gene %in% plot.df.pTA$gene) {
    genes = append(genes,gene)
    temp = z[z$V7 == gene,]
    num = append(num,nrow(temp))
    dir = append(dir,unique(temp$dir))

    pro.change = plot.df.pTA[plot.df.pTA$gene == gene &
                              plot.df.pTA$sample.conditions == 60,]$value -
      plot.df.pTA[plot.df.pTA$gene == gene & plot.df.pTA$sample.conditions == 0,]$value
    pro.changes = append(pro.changes,pro.change)

    atac.change = 0
    for(peak in temp$ATAC.peak) {
      if(peak %in% plot.df.atac$genes) {
        atac.change = sum(atac.change,
                          (plot.df.atac[plot.df.atac$genes == peak &
                                          plot.df.atac$sample.conditions == 60,]$value -
                             plot.df.atac[plot.df.atac$genes == peak & plot.df.atac$sample.conditions == 0,]$value))
      }
    }
    atac.changes = append(atac.changes,atac.change)
  }
}

plot.df = data.frame(genes,num,dir,pro.changes,atac.changes)

pdf('genes.near.only.up.or.down.peaks.pdf',width=9,height=9)
print(
  ggplot(plot.df,aes(x=num,y=pro.changes,dir=dir)) +
    geom_jitter(aes(color=atac.changes,size=abs(atac.changes)),
                show.legend = TRUE,width=0.2,data = subset(plot.df,dir == 'Increased')) +
    scale_color_continuous('Magnitude Total \n ATAC Change \n Increasing Peaks',low='pink',high='dark red') +
    new_scale_color() +
    geom_jitter(aes(color=atac.changes,size=abs(atac.changes)),
                show.legend = TRUE,width=0.2,data = subset(plot.df,dir == 'Decreased')) +
    scale_color_continuous('Magnitude Total \n ATAC Change \n Decreasing Peaks',high='light blue',low='dark blue') +

    theme_minimal() +
    labs(y = 'Txn Change in First Hour', x = '# of Increased / Decreased Peaks Near Gene',
         title='Genes That Are Only Near Either Increased or Decreased Peaks',
         size = 'Magnitude Total ATAC Change') +
    theme(
      panel.grid.minor = element_blank(),
      plot.title = element_text(size=14,face='bold',hjust = 0.5),
      axis.ticks = element_blank(),
      axis.text = element_text(size=11,face='bold'),
      axis.title = element_text(size=12,face='bold'),
      legend.text = element_text(size=10,face='bold'),
      legend.title = element_text(size=11,face='bold')) +
    scale_x_continuous(breaks = seq(0,10,1)) +
    scale_y_continuous(breaks = seq(-4,4,1))
)
dev.off()
```

4.2 Evaluating transcription v. accessibility across the genome

To expand the previous analysis, we evaluated the relationship between transcription and accessibility at various comparisons throughout the time course. For each pairwise time point comparison we identify significantly differentially expressed genes and then plot the change in transcription of that genes over the comparison against the distance-scaled change in local accessibility. This is Figure 3J and K.

```
library(ggplot2)

mkdir('access_v_txn')
setwd('access_v_txn')

load('../final.atac.all.df.Rdata')
load('../final.pro.all.df.Rdata')

time.pts = c(0,20,40,60,120,180,240)

comparisons = c()
for (i in 1:(length(time.pts)-1)) {
  for (j in (i+1):length(time.pts)) {
    comparisons = append(comparisons,paste0(time.pts[j],'.v.', time.pts[i]))
  }
}

for(k in 1:length(comparisons)) {
```

```
col1 = grep(paste0('response.',comparisons[k]),colnames(final.atac.all.df))
col2 = grep(paste0('response.',comparisons[k]),colnames(final.pro.all.df))

x = read.table('../all_dynamic_genes_near_peaks.bed')

x$location = paste0(x$V1,':',x$V2,'-',x$V3)
x$peak.type = 'ATAC'

x$peak.dir = 'NA'
x$gene.dir = 'NA'
for(i in 1:nrow(x)) {
  x$peak.dir[i] = final.atac.all.df[rownames(final.atac.all.df) == x$location[i],col1]
  x$gene.dir[i] = final.pro.all.df[final.pro.all.df$gene == x$V7[i],col2]
}

x = x[!x$gene.dir %in% c('All Other Genes','Unchanged'),]

dyn.genes = c()
for(i in 1:length(unique(x$V7))) {
  temp = x[x$V7 == unique(x$V7)[i],]
  if('Decreased' %in% temp$peak.dir || 'Increased' %in% temp$peak.dir) {
    dyn.genes = append(dyn.genes,unique(x$V7)[i])
  }
}

df = x[x$V7 %in% dyn.genes,]

if(nrow(df) > 0) {
  z = data.frame(gene = unique(df$V7),x=NA,y=NA)

  col1 = grep(paste0('log2FoldChange.',comparisons[k]),colnames(final.pro.all.df))
  col2 = grep(paste0('log2FoldChange.',comparisons[k]),colnames(final.atac.all.df))

  for(i in 1:nrow(z)) {
    z$y[i] = final.pro.all.df[final.pro.all.df$gene == z$gene[i],col1]
    temp = df[df$V7 == z$gene[i],]
    for(j in 1:nrow(temp)) {
      gene = temp$V7[j]
      tss = final.pro.all.df[final.pro.all.df$gene == gene,]$TSS
      summit = temp$V2[j] + 100

      temp$dist[j] = abs(summit-tss)
      if(temp$dist[j] == 1 || temp$dist[j] == 0) {
        temp$dist[j] = 1.258925
      }

      temp$acc.change[j] = final.atac.all.df[rownames(final.atac.all.df) == temp$location[j],col2]/ log(temp$dist[j],10)

    }
    z$x[i] = sum(temp$acc.change)
  }

  pdf(paste0('txn.v.access.',comparisons[k],'.pdf'))
  print(
    ggplot(z,aes(x=x,y=y)) +
      geom_point(size=1) +
      geom_hline(yintercept = 0) +
      geom_vline(xintercept = 0) +
      #geom_smooth(method = 'lm',se=FALSE) +
      theme_minimal() +
      labs(y = 'log10 Transcription Fold Change',
           x = 'Environment Score',
           title=paste0('Txn v. Access ',comparisons[k],' Only Sig Changed Genes')) +
      theme(panel.grid.minor = element_blank(),
            plot.title = element_text(size=14,face='bold',hjust = 0.5),
            axis.ticks = element_blank(),
            axis.text = element_text(size=12,face='bold'),
            axis.title = element_text(size=14,face='bold'))
  )
  dev.off()

  print(comparisons[k])
  print((nrow(z[z$x > 0 & z$y > 0,]) + nrow(z[z$x < 0 & z$y < 0,])) / nrow(z))

  fam.df = data.frame(fam = c('AP1','CEBP','GR','KLF','SP','TWIST'),
                      dir =  c(rep('Increased',4),rep('Decreased',2)))

  for(i in 1:nrow(fam.df)) {
    fam = fam.df[i,]$fam
    colnum = which(colnames(final.atac.all.df) == fam)
    temp = final.atac.all.df[rownames(final.atac.all.df) %in% df$location,]
    temp = temp[!is.na(temp[,colnum]),]
    col1 = which(colnames(final.atac.all.df) == paste0('response.',comparisons[k]))
    dir = fam.df[fam.df$fam == fam,]$dir
    temp = temp[temp[,col1] == dir,]
```

```
      family.peaks = rownames(temp)

      if(length(family.peaks)  > 1) {
        chr = sapply(strsplit(unique(family.peaks), ':'), '[', 1)
        suffix = sapply(strsplit(unique(family.peaks), ':'), '[', 2)
        start = sapply(strsplit(suffix, '-'), '[', 1)
        end = sapply(strsplit(suffix, '-'), '[', 2)

        write.table(data.frame(chr,start,end),
                    file=paste0(fam,'.',dir,'.peaks.',comparisons[k],'.bed'),
                    sep = '\t',quote = F, row.names = F, col.names = F)
      }

      family.genes = x[x$location %in% rownames(temp),]$V7

      temp2 = z[z$gene %in% family.genes,]

      if (nrow(temp2) > 1) {

        temp2$col = 'black'
        if(dir == 'Decreased') {
          if(nrow(temp2[temp2$y < 0 & temp2$x < 0,]) > 0) {
            temp2[temp2$y < 0 & temp2$x < 0,]$col = 'blue'
            write(temp2[temp2$y < 0 & temp2$x < 0,]$gene,
                  file=paste0('family.',fam,'.down.genes.',comparisons[k],'.txt'))
          }
        } else {
          if(nrow(temp2[temp2$y > 0 & temp2$x > 0,]) > 0) {
            temp2[temp2$y > 0 & temp2$x > 0,]$col = 'red'
            write(temp2[temp2$y > 0 & temp2$x > 0,]$gene,
                  file=paste0('family.',fam,'.up.genes.',comparisons[k],'.txt'))
          }
        }

        pdf(paste0(fam,'.',comparisons[k],'.txn.v.access.pdf'))
        print(
          ggplot(temp2,aes(x=x,y=log(y,10))) +
            geom_point(size=1,color=temp2$col) +
            geom_hline(yintercept = 0) +
            geom_vline(xintercept = 0) +
            theme_minimal() +
            labs(y = 'log10 Transcription Fold Change',
                 x = 'log10 Accessibility Fold Change / log10 Distance',
                 title=paste0(fam,' Txn v. Access ',comparisons[k])) +
            theme(panel.grid.minor = element_blank(),
                  plot.title = element_text(size=14,face='bold',hjust = 0.5),
                  axis.ticks = element_blank(),
                  axis.text = element_text(size=12,face='bold'),
                  axis.title = element_text(size=14,face='bold'))
        )
        dev.off()
      }
    }
  }
}
```

4.3 Using cumulative distribution functions to assess functional distances

We use a cumulative distribution function to evaluate whether peaks containing a specific factor motif (and only that motif) are generally closer to activated, repressed, or unchanged genes. For example, GR peaks are closer to activated genes than they are to repressed or unchanged genes. Furthermore, we identify the distance at which the difference in CDFs between activated / repressed genes and unchanged genes plateaus. This distance represents an inferred maximal actionable distance of the factor in this system. This analysis also identifies the pairwise comparisons throughout the time course for which each factor is significantly closer to either activated or repressed. These comparisons provide the framework for our time-related rules when inferring networks below. This analysis is Figure 3C and E.

```
source('https://raw.githubusercontent.com/mjg54/znf143_pro_seq_analysis/master/docs/ZNF143_functions.R')
library(lattice)
library(latticeExtra)


system('mkdir ecdf')
setwd('ecdf')

load('../final.atac.all.df.Rdata')
load('../final.pro.all.df.Rdata')
```

```
get.tss <- function(bedfile) {
  if (ncol(bedfile) > 6) {
    bedfile = bedfile[,c(1:6)]
  }
  for (i in 1:nrow(bedfile)) {
    if (bedfile[i,'strand'] == '+') {
      bedfile[i,3] = bedfile[i,2] + 1
    } else {
      bedfile[i,2] = bedfile[i,3] - 1
    }
  }
  return(bedfile)
}

bedTools.closest<-function(functionstring="/usr/local/bin/bedtools/closestBed",bed1,bed2,opt.string="") {
  #create temp files
  #a.file=tempfile()
  #b.file=tempfile()
  #out   =tempfile()
  #bed1[,1] = as.character(bed1[,1])
  #bed2[,1] = as.character(bed2[,1])
  #bed1[,2] = as.numeric(as.character(bed1[,2]))
  #bed2[,2] = as.numeric(as.character(bed2[,2]))
  #bed1[,3] = as.numeric(as.character(bed1[,3]))
  #bed2[,3] = as.numeric(as.character(bed2[,3]))

  #bed1 =  bed1[do.call(order, bed1[,c(colnames(bed1)[1], colnames(bed1)[2])]),]
  #bed2 =  bed2[do.call(order, bed2[,c(colnames(bed2)[1], colnames(bed2)[2])]),]
  options(scipen =99) # not to use scientific notation when writing out

  #write bed formatted dataframes to tempfile
  write.table(bed1,file= 'a.file.bed', quote=F,sep="\t",col.names=F,row.names=F)
  write.table(bed2,file= 'b.file.bed', quote=F,sep="\t",col.names=F,row.names=F)

  # create the command string and call the command using system()
  command1=paste('sort -k1,1 -k2,2n', 'a.file.bed', '> a.file.sorted.bed')
  #cat(command1,"\n")
  try(system(command1))
  command2=paste('sort -k1,1 -k2,2n', 'b.file.bed', '> b.file.sorted.bed')
  #cat(command2,"\n")
  try(system(command2))

  command=paste(functionstring, opt.string,"-a",'a.file.sorted.bed',"-b",'b.file.sorted.bed',">",'out.file.bed',sep=" ")
  #cat(command,"\n")
  try(system(command))

  res=read.table('out.file.bed',header=F, comment.char='')
  #unlink(a.file);unlink(b.file);unlink(out)
  colnames(res) = c(colnames(bed1), colnames(bed2)[1:ncol(bed2)], 'dis' )
  return(res)
}

cdf.deseq.df.arun <- function(deseq.df = final.pro.all.df, name.col, type.peak = 'ATAC',
                              peaks = final.atac.all.df, tf.motif = 'CEBP',
                              dir = 'Increased',
                              closestBedDir = '/usr/local/bin/closestBed') {

  deseq.df = deseq.df[!(deseq.df$chr %in% c('chrM','chrY')),]

  bed.tss.activated =  get.tss(deseq.df[deseq.df[,name.col] == 'Activated',])[,c(1:4,6,5)]
  #paste(head(bed.tss.activated))
  bed.tss.repressed = get.tss(deseq.df[deseq.df[,name.col] == 'Repressed',])[,c(1:4,6,5)]
  bed.tss.unchanged = get.tss(deseq.df[deseq.df[,name.col] == 'Unchanged',])[,c(1:4,6,5)]
  bed.tss.dregs = get.tss(deseq.df[deseq.df[,name.col] == 'All Other Genes',])[,c(1:4,6,5)]
  peaks = peaks[!is.na(peaks[,tf.motif]),]
  #only peaks that increase/decrease?
  #peaks = peaks[!is.na(peaks[,tf.motif]) & peaks[,name.col] == dir,]
  act.distance = bedTools.closest(functionstring= closestBedDir,
                                bed1 = bed.tss.activated, bed2 = peaks[,c(1:3)], opt.string = '-D a')
  unreg.distance = bedTools.closest(functionstring= closestBedDir,
                                  bed1 = bed.tss.unchanged, bed2 = peaks[,c(1:3)], opt.string = '-D a')
  repress.distance = bedTools.closest(functionstring= closestBedDir,
                                    bed1 = bed.tss.repressed, bed2 = peaks[,c(1:3)], opt.string = '-D a')
  dregs.distance = bedTools.closest(functionstring= closestBedDir,
                                  bed1 = bed.tss.dregs, bed2 = peaks[,c(1:3)], opt.string = '-D a')

  #if there isn't at least one peak / chr
  if(-1 %in% act.distance[,8] | -1 %in% unreg.distance[,8] | -1 %in% repress.distance[,8] | -1 %in% dregs.distance[,8]) {
    return(print('BREAK'))
  }

  df.up.can = cbind(act.distance[,c(4, 10)], 'Activated', "Canonical")
  df.un.can = cbind(unreg.distance[,c(4, 10)], 'Unchanged', "Canonical")
  df.down.can = cbind(repress.distance[,c(4, 10)], 'Repressed', "Canonical")
  df.dregs.can = cbind(dregs.distance[,c(4, 10)], cat = 'All Other Genes', "Canonical")
```

```
colnames(df.up.can) = c(colnames(df.up.can)[1:2], 'status', 'er')
colnames(df.un.can) = c(colnames(df.up.can)[1:2], 'status', 'er')
colnames(df.down.can) = c(colnames(df.up.can)[1:2], 'status', 'er')
colnames(df.dregs.can) = c(colnames(df.up.can)[1:2], 'status', 'er')

df.all = rbind(df.up.can, df.un.can, df.down.can)

#df.all = rbind(df.up.can, df.un.can, df.down.can, df.dregs.can)
#change distance to 1 if peak overlaps TSS
if(0 %in% df.all$dis) {
  df.all[df.all$dis == 0,]$dis = 1
}
#only w/in 10kb?
#df.all = df.all[abs(df.all$dis) < 10000,]
#

act.p.value = ks.test(x = log(abs(df.all$dis)[df.all$status == 'Activated']),
                      y = log(abs(df.all$dis)[df.all$status == 'Unchanged']))$p.value
rep.p.value = ks.test(x = log(abs(df.all$dis)[df.all$status == 'Repressed']),
                      y = log(abs(df.all$dis)[df.all$status == 'Unchanged']))$p.value

if(act.p.value < 0.001 | rep.p.value < 0.001) {

  pdf(paste0("cdf_", tf.motif, "_", type.peak, "_", name.col, ".pdf"), width=4.2, height=3.83)

  col.lines = c("#FF0000", "#0000FF", "grey60")
  print(ecdfplot(~log(abs(dis), base = 10), groups = status, data = df.all,
                 auto.key = list(lines=TRUE, points=FALSE),
                 col = col.lines,
                 aspect = 1,
                 scales=list(relation="free",alternating=c(1,1,1,1)),
                 ylab = 'Cumulative Distribution Function',
                 xlab = expression('log'[10]~'peak w/motif Distance from TSS'),
                 between=list(y=1.0),
                 type = 'a',
                 xlim = c(0,max(log(abs(df.all$dis), base = 10))),
                 lwd=2,
                 par.settings = list(superpose.line = list(col = col.lines, lwd=3),
                                     strip.background=list(col="grey85")),
                 panel = function(...) {
                   panel.abline(v= 200, lty =2)
                   panel.ecdfplot(...)
                 }))
  dev.off()

  #distance calc
  if(dir == 'Increased') {
    p.value = act.p.value
  } else {
    p.value = rep.p.value
  }

  if(p.value < 0.001) {

    df.all$status = as.character(df.all$status)

    if(dir == 'Increased') {
      gene.dir = 'Activated'
    } else {
      gene.dir = 'Repressed'
    }

    act = ecdf(abs(df.all$dis)[df.all$status == gene.dir])
    unc = ecdf(abs(df.all$dis)[df.all$status == 'Unchanged'])

    act.y = seq(0, 2000000, by=1000)
    unc.y = seq(0, 2000000, by=1000)

    #can play with nknots as we did for SP
    #600
    spl = smooth.spline(act.y, act(act.y) - unc(unc.y), nknots = 100)
    pred = predict(spl)

    #find the point at which the tangent of the spline goes from positive to negative
    pred1 = predict(spl, deriv=1)

    print('the distance that CDFs are parallel or converge')
    print(act.y[min(which(pred1$y<=0)) - 1])
    pred$y[pred$y < 0] <- 0

    dist = act.y[min(which(pred1$y<=0)) - 1]

    comparison = strsplit(name.col,'response.')[[1]][2]
    pdf(paste0("empirical_distance_determination_",tf.motif,"_",comparison,".pdf"),
        width=3.83, height=3.83, useDingbats=FALSE)
    par(pty="s")
```

```
      plot(act.y, act(act.y) - unc(unc.y), cex=0.5, xlim = c(0, 200000),
           xlab = paste0(tf.motif,' distance from TSS (bp)'),
           ylab = paste0(gene.dir,' Genes CDF - Unchanged genes CDF'))
      abline(v = act.y[min(which(pred1$y<=0)) - 1], col =2, lty =2)
      lines(pred, col = 'blue', lwd =1)
      dev.off()
    }

    write(paste0(tf.motif,'\t',name.col,'\t',type.peak,'\t',act.p.value,
                 '\t',rep.p.value,'\t',dist),file=paste0(tf.motif,"_ecdf_pvalues.txt"),append=TRUE)
  }
  #return(df.all)

}

write(paste0('tf.motif','\t','name.col','\t','type.peak','\t','act.p.value',
             '\t','rep.p.value','\t','dist'),file="ecdf_pvalues.txt",append=TRUE)

time.pts = c(0,20,40,60,120,180,240)

comparisons = c()
for (i in 1:(length(time.pts)-1)) {
  for (j in (i+1):length(time.pts)) {
    comparisons = append(comparisons,paste0(time.pts[j],'.v.', time.pts[i]))
  }
}

family.df = data.frame(
  factors = colnames(final.atac.all.df[13:18]),
  dir = c('Increased','Increased','Increased','Increased','Decreased','Decreased')
)

for(factor in family.df$factors) {

  print(factor)
  atac.dir = family.df[family.df$factors == factor,]$dir

  temp = final.atac.all.df[final.atac.all.df$response.time.course == 'Dynamic',]
  temp = temp[,13:18]
  colnum = which(colnames(temp) == factor)
  other.cols = c(1:6)[-colnum]
  temp = temp[!is.na(temp[,colnum]),]
  for(num in other.cols) {
    temp = temp[is.na(temp[,num]),]
  }
  peaks = final.atac.all.df[rownames(final.atac.all.df) %in% rownames(temp),]

  for(comparison in comparisons) {
    print(comparison)
    print('ATAC')
    cdf.deseq.df.arun(deseq.df = final.pro.all.df,
                      type.peak = 'ATAC',
                      peaks = peaks,
                      name.col = paste0('response.',comparison),
                      tf.motif = factor,
                      dir = atac.dir,
                      closestBedDir = '/usr/local/bin/closestBed')

  }
}
```

4.4 Inferring *trans* and *cis*-edges

We implement a set of simple rules to infer *trans*-edges between TFs and regulatory elements and *cis*-edges between regulatory elements and genes. The rules are laid out in the code below. But first we need to create an object that contains the gene names of all members of our TF families.

```
system('wget https://raw.githubusercontent.com/guertinlab/adipogenesis/master/Pipeline_PRO/TFClass_to_Gencode.csv')
x = read.csv('TFClass_to_Gencode.csv')
x[x==""] = NA
colnames(x) = gsub('X','',colnames(x))

key = data.frame()
for (col in 1:ncol(x)) {
    y = x[,col]
    y = as.vector(y[!is.na(y)])
    key = rbind(key,data.frame(y, rep(names(x)[col],length(y))))
}
colnames(key) = c('tf','class')

df = data.frame()
```

```
families = c(1:19)

for(fam in Sys.glob(file.path('PSWM_family_*txt'))) {
    x = read.table(fam)[,1]
    x = sapply(strsplit(x, '_'), '[', 1)
    x = gsub('::','_',x)
    first = sapply(strsplit(x, '_'), '[', 1)
    second = sapply(strsplit(x, '_'), '[', 2)
    x = as.vector(na.omit(c(first,second)))
    x = gsub(':','_',x)
    first = sapply(strsplit(x, '_'), '[', 1)
    second = sapply(strsplit(x, '_'), '[', 2)
    x = as.vector(na.omit(c(first,second)))
    num = strsplit(strsplit(strsplit(fam,'/')[[1]][6],'_')[[1]][3],'.txt')[[1]][1]
    y = rep(num, length(x))
    df = rbind(df,data.frame(factors=x,fam=y))
}

func <- function(str) {
     first = toupper(strsplit(str,'')[[1]][1])
     rest = paste0(tolower(strsplit(str,'')[[1]][2:nchar(str)]),collapse='')
     return(paste0(first,rest))
}

df$factors = sapply(df$factors,func)

func <- function(str) {
  return(key[key$tf == str,]$class)
}

df$class = sapply(df$factors,func)

#manual annotation
z = data.frame(tf = c('Fra1','Fra2','Jun-ap1','Nf-e2','Nrf2',
                      'Are','Gre','Pr','Eklf','Nrf','Stat5',
                      'Tcfap2e','Znf263','Ap4','Nfy','Ets','Gfx'),
               class = c('1.1.2.1','1.1.2.1','1.1.1.1','1.1.1.2','1.1.1.2',
                         '2.1.1.1','2.1.1.1','2.1.1.1','2.3.1.2','0.0.6.0',
                         '6.2.1.0','1.3.1.0','2.3.3.0','1.2.6.4','4.2.1.0',
                         '3.5.2.1','2.3.2.1'))

for(tf in z$tf) {
    df[df$factors == tf,]$class = z[z$tf == tf,]$class
}

df$class = as.character(df$class)

df = df[!(df$class == 'character(0)'),]

tf.genes = data.frame()
for(fam in families) {
    print(fam)
    print(unique(df[df$fam == fam,]$class))
    no.factors = 0
    factors = c()
    for(class in unique(df[df$fam == fam,]$class)) {
        factors = append(factors,key[key$class == class,]$tf)
        no.factors = no.factors + nrow(key[key$class == class,])
    }
    temp = data.frame(fam = rep(fam,no.factors),factors = factors)
    tf.genes = rbind(tf.genes,temp)
    print(no.factors)
    cat("\n")
}

family.tf.class = df
save(family.tf.class,file='family.tf.class.Rdata')
save(tf.genes,file='tf.genes.Rdata')
```

Now we infer *cis* and *trans*-links.

```
load('final.atac.all.df.Rdata')
load('final.pro.all.df.Rdata')

#add TF genes data and size corrected read counts to final.pro.all.df
load('tf.genes.Rdata')

tf.genes$name = ''
tf.genes[tf.genes$fam == 1,]$name = 'AP1'
tf.genes[tf.genes$fam == 2,]$name = 'bHLH'
tf.genes[tf.genes$fam == 3,]$name = 'GR'
tf.genes[tf.genes$fam == 4,]$name = 'Bhlha15'
tf.genes[tf.genes$fam == 5,]$name = 'CEBP'
tf.genes[tf.genes$fam == 6,]$name = 'CTCF'
tf.genes[tf.genes$fam == 7,]$name = 'SP'
tf.genes[tf.genes$fam == 8,]$name = 'ELK/ETV'
```

```
tf.genes[tf.genes$fam == 9,]$name = 'ZBTB33'
tf.genes[tf.genes$fam == 10,]$name = 'Maz'
tf.genes[tf.genes$fam == 11,]$name = 'NFI'
tf.genes[tf.genes$fam == 12,]$name = 'NFY'
tf.genes[tf.genes$fam == 13,]$name = 'NRF'
#tf.genes[tf.genes$fam == 14,]$name = 'Nur77'
tf.genes[tf.genes$fam == 15,]$name = 'STAT'
tf.genes[tf.genes$fam == 16,]$name = 'AP2'
tf.genes[tf.genes$fam == 17,]$name = 'TEAD'
tf.genes[tf.genes$fam == 18,]$name = 'TWIST'
tf.genes[tf.genes$fam == 19,]$name = 'ZNF263'
tf.genes = tf.genes[-grep('^Egr',tf.genes$factors),]
tf.genes[grep('Klf',tf.genes$factors),]$name = 'KLF'

final.pro.all.df$factor.family = NA
for(i in 1:nrow(tf.genes)) {
  if(tf.genes$factors[i] %in% final.pro.all.df$gene) {
    final.pro.all.df[final.pro.all.df$gene == tf.genes$factors[i],]$factor.family = tf.genes$name[i]
  }
}

final.pro.all.df[,(ncol(final.pro.all.df) + 1):(ncol(final.pro.all.df) + 7)] =
  (final.pro.all.df[,9:15])/(final.pro.all.df$size/1000)
colnames(final.pro.all.df)[(ncol(final.pro.all.df)-6):ncol(final.pro.all.df)] =
  paste0('size.corrected.min.',c(0,20,40,60,120,180,240))
expression.df = final.pro.all.df[,(ncol(final.pro.all.df)-7):ncol(final.pro.all.df)]
expression.df = na.omit(expression.df)

#define comparisons
time.pts = c(0,20,40,60,120,180,240)

start = c()
end = c()
comparisons = c()
for (i in 1:(length(time.pts)-1)) {
  for (j in (i+1):length(time.pts)) {
    start = append(start,time.pts[i])
    end = append(end,time.pts[j])
    comparisons = append(comparisons,paste0(time.pts[j],'.v.', time.pts[i]))
  }
}
comparisons.df = data.frame(comparisons,start,end, stringsAsFactors = FALSE)

#assign factor to up or down
family.df=data.frame(factors = c('AP1','CEBP','GR','KLF','SP','TWIST'),
                     dirs = c(rep('Increased',4),rep('Decreased',2)), stringsAsFactors = FALSE)

#define edges
trans.links = data.frame(stringsAsFactors = FALSE)
cis.links = data.frame(stringsAsFactors = FALSE)

#loop through factors
for(factor in family.df$factors) {
  options(stringsAsFactors=FALSE)
  print(factor)
  dir = family.df[family.df$factors == factor,]$dirs
  temp = expression.df[expression.df$factor.family == factor,]

  #loop through time points and decide whether or not the factor is active at that time point
  #skip 240
  for(i in 1:(length(time.pts)-1)) {
    time = time.pts[i]
    #Rule 1: is the comparison consistent with accessibility changes?
    #based on qualitative observation of Fig 1F
    #GR - not active > 40
    #SP - not active < 40

    if(factor == 'GR' & time > 40) {
      next
    }

    if(factor == 'SP' & time < 40) {
      next
    }

    #Rule 2: is at least one factor gene expressed at the time?
    colnum = which(colnames(temp) == paste0('size.corrected.min.',time))
    if(factor != 'SP' & !(any(temp[,colnum] > 10))) {
      next
    }

    #Rule 3: if time is not 0, then at least one factor gene must be activated in the time.v.0 comparison
    colnum = which(colnames(final.pro.all.df) == paste0('response.',time,'.v.0'))
    temp$response = final.pro.all.df[rownames(final.pro.all.df) %in% rownames(temp),colnum]
    #if factor is expressed at 0, it can be active at 0 minutes
    if(time != 0) {
```

```
    if(factor != 'SP' & !(any(temp$response == 'Activated'))) {
      next
    } else if(factor == 'SP' & !(any(temp$response == 'Repressed'))) {
      next
    }

    if(factor != 'SP') {
      temp = temp[temp$response == 'Activated',]
    } else {
      temp = temp[temp$response == 'Repressed',]
    }

    colnum = which(colnames(temp) == paste0('size.corrected.min.',time))
    colnum2 = colnum - 1
    temp$diff = (temp[,colnum] - temp[,colnum2]) / temp[,colnum]
    #Rule 4: factor must be increased in expression from last time point (not necessarily significantly 'Activated')
    #this ensures the factor expression is going up by at least 10%
    #leading into this time point as opposed to remaining stable or decreasing from a higher level
    #if(factor != 'SP' & !(any(temp$diff > 0.1))) {
    #  next
    #} else if(factor == 'SP' & !(any(temp$diff < -0.1))) {
    #  next
    #}
  }

  #if the factor is active at the time point, then figure out what peaks are increasing/decreasing

  #Rule 5: does the peak have the factor motif?
  factor.col = which(colnames(final.atac.all.df) == paste0(factor))
  factor.df = final.atac.all.df[!is.na(final.atac.all.df[,factor.col]),]

  #Rule 6: is the peak increasing/decreasing from that time point to the next by at least 10%
  #this makes sure the peak is actually being activated at the time pt and is not going up later
  #for ex. if gene goes up from 0 to 20 and peak goes up from 60 to 120, then 120.v.20 for the peak will be 'Increased'
  #so make sure peak is going up AT the time pt and not just some time after the time pt
  colnum = which(colnames(final.atac.all.df) == paste0('min.',time))
  colnum2 = which(colnames(final.atac.all.df) == paste0('min.',time.pts[i+1]))

  if(!(factor %in% c('SP','TWIST'))) {
    factor.df = factor.df[factor.df[,colnum2] > (1.1*factor.df[,colnum]),]
  } else {
    factor.df = factor.df[factor.df[,colnum2] < (0.9*factor.df[,colnum]),]
  }

  #Rule 7: does the peak significantly change in a future comparison?
  future.comps = comparisons.df[comparisons.df$start == time,]$comparisons
  peaks = c()
  #comps = c()

  for(comp in future.comps) {
    colnum = which(colnames(factor.df) %in% paste0('response.',comp))

    if(!(factor %in% c('SP','TWIST'))) {
      if(nrow(factor.df[factor.df[,colnum] == 'Increased',]) > 0) {
        peaks = unique(append(peaks,rownames(factor.df[factor.df[,colnum] == 'Increased',])))
        #comps = append(comps,rep(comp,nrow(factor.df[factor.df[,colnum] == 'Increased',])))
      }
    } else {
      if(nrow(factor.df[factor.df[,colnum] == 'Decreased',]) > 0) {
        peaks = unique(append(peaks,rownames(factor.df[factor.df[,colnum] == 'Decreased',])))
        #comps = append(comps,rep(comp,nrow(factor.df[factor.df[,colnum] == 'Decreased',])))
      }
    }
  }

  #add trans edges to links df
  if(length(peaks) > 0) {
    links = data.frame(source = factor,
                       target = peaks,
                       time = rep(time,length(peaks)),
                       type = 'trans', stringsAsFactors = FALSE)
    trans.links = rbind(trans.links,links)
  }
}

#cis-edges
genes.near.peaks = read.table('all_genes_near_peaks.bed', stringsAsFactors = FALSE)
genes.near.peaks$ATAC.peak = paste0(genes.near.peaks$V1,':',genes.near.peaks$V2,'-',genes.near.peaks$V3)

factor.links = trans.links[trans.links$source == factor,]

x = genes.near.peaks[genes.near.peaks$ATAC.peak %in% factor.links$target,]

for(peak in unique(factor.links$target)) {
  near.genes = x[x$ATAC.peak == peak,]$V7
  potential.times = factor.links[factor.links$target == peak,]$time
```

```
    comparisons = paste0('response.',comparisons.df[comparisons.df$start %in% potential.times,]$comparisons)

    cdf = read.table(paste0('ecdf/',factor,'_ecdf_pvalues.txt'), stringsAsFactors = FALSE)
    cdf$activated.fdr = p.adjust(cdf[,4])
    cdf$repressed.fdr = p.adjust(cdf[,5])
    if(dir == 'Increased') {
      comparisons = comparisons[comparisons %in% cdf[cdf$activated.fdr < 0.01,]$V2]
      gene.dir = 'Activated'
    } else {
      comparisons = comparisons[comparisons %in% cdf[cdf$repressed.fdr < 0.01,]$V2]
      gene.dir = 'Repressed'
    }

    for(time in potential.times) {
      colnum = which(colnames(final.pro.all.df) == paste0('min.',time))
      colnum2 = colnum + 1

      for(gene in near.genes) {

        #Rule 8: is the gene Activated/Repressed in a comparison that is significant in the CDF?
        if(!(any(final.pro.all.df[final.pro.all.df$gene == gene,comparisons] == gene.dir))) {
          next
        }

        #Rule 9: is the distance w/in the acceptable distance defined by the cdf?
        dist = abs(final.pro.all.df[final.pro.all.df$gene == gene,'TSS'] -
                     (as.numeric(final.atac.all.df[rownames(final.atac.all.df) == peak,'start']) + 100))
        dist.thresh = data.frame(comparisons =
                                   comparisons[which(final.pro.all.df[final.pro.all.df$gene ==
                                                                        gene,comparisons] == gene.dir)],
                                 thresh = 0)

        for(comparison in dist.thresh$comparisons) {
          dist.thresh[dist.thresh$comparisons == comparison,'thresh'] = as.numeric(cdf[cdf$V2 == comparison,'V6'])
        }
        if(!any(dist < dist.thresh$thresh)) {
          next
        }

        #Rule 10: is the gene increasing or decreasing at the time the peak is increasing?
        if(!(factor %in% c('SP','TWIST')) &
           !(final.pro.all.df[final.pro.all.df$gene == gene,colnum2] >
             (1.1*final.pro.all.df[final.pro.all.df$gene == gene,colnum]))) {
          next
        } else if (factor %in% c('SP','TWIST') &
                   !(final.pro.all.df[final.pro.all.df$gene == gene,colnum2] <
                     (0.9*final.pro.all.df[final.pro.all.df$gene == gene,colnum]))){
          next
        }


        cis.links = unique(rbind(cis.links,
                                 c(peak,
                                   gene,
                                   time,
                                   'cis',
                                   factor)))
      }
    }
  }
}
colnames(cis.links) = c('source','target','time','type','factor')

save(cis.links,file='cis.links.Rdata')
save(trans.links,file='trans.links.Rdata')
```

Once we have defined *cis* and *trans* links we can identify regulatory elements and genes regulated by individual factors or combinations of TFs.

```
family.df=data.frame(factors = c('AP1','CEBP','GR','KLF','SP','TWIST'),
                     dirs = c(rep('Increased',4),rep('Decreased',2)), stringsAsFactors = FALSE)

for(factor in unique(cis.links$factor)) {
  print(factor)

  dir = family.df[family.df$factors == factor,"dirs"]
  other.factors = family.df[family.df$dirs == dir & family.df$factors != factor,'factors']

  #all.factor.peaks = unique(trans.links[trans.links$source == factor,]$target)
  shared.peaks = unique(trans.links[trans.links$source == factor &
                                      trans.links$target %in%
                                      trans.links[trans.links$source %in% other.factors,]$target,]$target)
  isolated.peaks = unique(trans.links[trans.links$source == factor &
                                        !(trans.links$target %in%
                                            trans.links[trans.links$source %in% other.factors,]$target),]$target)
```

```
  shared.peaks.leaf = shared.peaks[shared.peaks %in% cis.links$source]
  shared.peaks.terminal = shared.peaks[!(shared.peaks %in% cis.links$source)]

  isolated.peaks.leaf = isolated.peaks[isolated.peaks %in% cis.links$source]
  isolated.peaks.terminal = isolated.peaks[!(isolated.peaks %in% cis.links$source)]

  all.factor.genes = unique(cis.links[cis.links$factor == factor,]$target)
  shared.genes = c()

  for(gene in all.factor.genes) {
    temp = cis.links[cis.links$target == gene,]
    #for '.stringent.genes.txt' use:
    #if(any(other.factors %in% temp$factor) | any(temp$source %in% shared.peaks)) {
    if(any(other.factors %in% temp$factor)) {
      shared.genes = append(shared.genes,gene)
    }
   }
  isolated.genes = all.factor.genes[!(all.factor.genes %in% shared.genes)]
  write.table(isolated.genes,file=paste0(factor,'.isolated.genes.txt'),sep='\t',quote=F,row.names=F,col.names=F)

  print(paste0('Shared Terminal Peaks: ',length(shared.peaks.terminal)))
  print(paste0('Isolated Terminal Peaks: ',length(isolated.peaks.terminal)))
  print(paste0('Shared Leaf Peaks: ',length(shared.peaks.leaf)))
  print(paste0('Isolated Leaf Peaks: ',length(isolated.peaks.leaf)))
  print(paste0('Shared Genes: ',length(shared.genes)))
  print(paste0('Isolated Genes: ',length(isolated.genes)))

  print('terminal: ')
  temp = unique(trans.links[trans.links$target %in% shared.peaks.terminal,1:2])
  print(table(temp$source))

  print('leaf: ')
  temp = unique(trans.links[trans.links$target %in% shared.peaks.leaf,1:2])
  print(table(temp$source))
}
```

4.5 Illustrating polymerase density and calculating pause indices

First we take genes solely regulated by a single TF. Then we calculate the location of the pause peak for each gene using the merged .bigWig files. Next we plot the composite polymerase density at two different time points. We repeat the process for all pairwise comparisons. The composite and box and whisker plots are found in Figure 4D-F and Supplemental Figure S4D-H.

```
library(lattice)

plot.composites <- function(dat, fact = 'GR', comp='20.v.0',
                            summit = 'TSS', class= '', num.m = -200,
                            num.p =90, y.low =0, y.high = 0.2,
                            col.lines = c(rgb(0,0,1,1/2),
                                          rgb(1,0,0,1/2),
                                          rgb(0.1,0.5,0.05,1/2),
                                          rgb(0,0,0,1/2),
                                          rgb(1/2,0,1/2,1/2),
                                          rgb(0,1/2,1/2,1/2),
                                          rgb(1/2,1/2,0,1/2)),
                            fill.poly = c(rgb(0,0,1,1/4),
                                          rgb(1,0,0,1/4),
                                          rgb(0.1,0.5,0.05,1/4),
                                          rgb(0,0,0,1/4),
                                          rgb(1/2,0,1/2,1/4))) {
  pdf(file = paste0('composites/composite_PolII_density_around_',fact,'_genes_',comp,'.pdf'),
      width=6,
      height=6)
  print(xyplot(est ~ x, group = time, data = dat,
               type = 'l',
               scales=list(x=list(cex=1,relation = "free",font=2), y =list(cex=1, relation="free",font=2)),
               col = col.lines,
               #main=list(label=class, cex=0.6),
               auto.key = list(points=F, lines=T, cex=1.2,font=2),
               par.settings = list(strip.background=list(col="grey85"),
                                   superpose.symbol = list(pch = c(16),
                                                           col=col.lines, cex =0.5),
                                   superpose.line = list(col = col.lines, lwd=c(2),
                                                         lty = c(1))),
               cex.axis=1.0,
               par.strip.text=list(cex=0.9, font=1, col='black'),
               aspect=1.0,
               between=list(y=0.5, x=0.5),
               #                 index.cond = list(c(1,3,4,2)),
               lwd=2,
               ylab = list(label = "PolII Density", cex =1.2,font=2),
```

```
               xlab = list(label = paste("Distance from ", summit, sep=''), cex =1.2,font=2),
               xlim = c(-200,1200)
  ))
  dev.off()
}

load('final.pro.all.df.Rdata')

#factors = c('AP1','CEBP','GR','KLF','SP','TWIST')
#time.strings = c('t0','20min','40min','60min','2hr','3hr','4hr')
#times = c(0,20,40,60,120,180,240)

factor.df <- data.frame(factors = c('AP1','CEBP','GR','KLF','SP','TWIST'),
                        dir = c(rep('Activated',4),rep('Repressed',2)))
time.df <- data.frame(time.strings = c('t0','20min','40min','60min','2hr','3hr','4hr'),
                      times = c(0,20,40,60,120,180,240))

comparisons = c()
for (i in 1:(length(time.df$times)-1)) {
  for (j in (i+1):length(time.df$times)) {
    comparisons = append(comparisons,paste0(time.df$times[j],'.v.', time.df$times[i]))
  }
}

upstream = 350
downstream = 1400
roll.avg = 10
step = 5

all.factors.all.times.composites = data.frame()
for(factor in factor.df$factors) {
  print(factor)
  dir = factor.df[factor.df$factors == factor,'dir']

  for(comparison in comparisons) {
    print(comparison)
    time1 <- strsplit(comparison,'.v.')[[1]][2]
    time2 <- strsplit(comparison,'.v.')[[1]][1]

    col1 = which(colnames(final.pro.all.df) == paste0('min.',time1))
    col2 = which(colnames(final.pro.all.df) == paste0('min.',time2))

    isolated.genes = read.table(paste0(factor,'.isolated.genes.txt'),sep='\t')[,1]

    x <- final.pro.all.df[final.pro.all.df$gene %in% isolated.genes,c(col1,col2)]
    x$diff <- x[,2] - x[,1]
    x$temp <- 'Repressed'
    x[x$diff > 0,'temp'] <- 'Activated'
    #table(x$temp)
    isolated.genes <- sapply(strsplit(rownames(x[x$temp == dir,]),'_'),'[',2)
    #

    bed.input = final.pro.all.df[final.pro.all.df$gene %in% isolated.genes,c(1:5,7)]
    bed.input[,7] = bed.input[,6] + 1
    bed.input[,8] = factor
    bed.input[,9] = 0
    #bed.input[,9] = times[i]
    colnames(bed.input) = c('chr', 'start', 'end', 'gene', 'strand', 'TSS','TSS+1','factor','time')

    tss.df = bed.input[,c(1,6:7,4,8,5)]
    tss.df[,3] = tss.df[,2] + 500
    tss.df[,2] = tss.df[,2] - 500

    #sum polymerase density for each position in window
    combined.plus.bw = load.bigWig('pro_plus_merged.bigWig')
    combined.minus.bw = load.bigWig('pro_minus_merged.bigWig')
    dat.x = bed6.step.probeQuery.bigWig(combined.plus.bw,combined.minus.bw, tss.df,
                                        step = 1, as.matrix = TRUE, op = "sum", follow.strand = FALSE)
    dat.x[is.na(dat.x)] <- 0
    dat.x.win = t(apply(dat.x, 1, function(x){rollapply(x, width = 50, FUN = mean,
                                                        by = 1, by.column = FALSE,align = "left")}))
    index.max = apply(dat.x.win, 1, which.max)
    the.max = apply(dat.x.win, 1, max)

    #find highest value in window to use as pause summit
    pause.df = tss.df
    pause.df[2][pause.df$strand == '-',] = pause.df[2][pause.df$strand == '-',] + index.max[pause.df$strand == '-']
    pause.df[2][pause.df$strand == '+',] = pause.df[2][pause.df$strand == '+',] + index.max[pause.df$strand == '+']
    #pause.df[3] = pause.df[2] + 1
    pause.df[3] = pause.df[2] + 50
    pause.df[,2] = rowMeans(pause.df[,2:3])
    pause.df[,3] = pause.df[,2] + 1

    bedTSSwindow = fiveprime.bed(pause.df, upstreamWindow = upstream,
                                 downstreamWindow = downstream)
    #plot.traces(bedTSSwindow$gene,title=paste0(factor,'.traces'))
```

```
    composite.df <- data.frame()
    for(time in c(time1,time2)) {
      string <- time.df[time.df$times == time,'time.strings']

      bwPlus = load.bigWig(paste0('~/Adipogenesis/PRO_analysis_redo/bigWigs/3T3_',
                                  string,'_plus_merged_normalized.bigWig'))
      bwMinus = load.bigWig(paste0('~/Adipogenesis/PRO_analysis_redo/bigWigs/3T3_',
                                   string,'_minus_merged_normalized.bigWig'))

      tss.matrix = bed6.step.bpQuery.bigWig(bwPlus, bwMinus , bedTSSwindow,
                                            step = step, as.matrix=TRUE, follow.strand=TRUE)

      coordin.start = (-upstream - 1)  + (step * roll.avg)/2
      coordin.end = downstream - (step * roll.avg)/2

      composite.lattice = data.frame(seq(coordin.start, coordin.end, by = step),
                                     rollmean(colMeans(tss.matrix), roll.avg),
                                     factor,
                                     time = time,
                                     stringsAsFactors = FALSE)

      colnames(composite.lattice) = c('x', 'est', 'factor', 'time')
      composite.lattice$x = as.numeric(composite.lattice$x)

      composite.df <- rbind(composite.df,composite.lattice)
    }
    composite.df$comparison <- comparison
    all.factors.all.times.composites <- rbind(all.factors.all.times.composites,composite.df)
  }
}

save(all.factors.all.times.composites,file='all.factors.all.times.composites.Rdata')

for(factor in factor.df$factors) {
  for(comparison in comparisons) {
    plot.composites(all.factors.all.times.composites[all.factors.all.times.composites$factor == factor &
                                                       all.factors.all.times.composites$comparison == comparison,],
                    fact=factor,
                    comp=comparison,
                    summit='Midpoint of Pause Peak')
  }
}
```

The next analysis is to compute polymerase density within the pause and gene body regions then calculate pause index changes between time points. Decreases in pause index indicates a reduction in polymerase density within the pause region and vice versa. Put in context with overall gene activation or repression these data suggest specfic transcription steps regulated by individual TFs. For example, a TF that increases overall polymerase density while decreasing pause index (like GR) may activate genes by inducing pause release and stimulating polymerase molecules to move into the gene body.

```
library(lattice)
library(bigWig)
library(zoo)

bwplot.input.pause <- function(bed.input, time='t0') {

  #isolate TSSs and define 1000 bp window centered around TSS
  tss.df = bed.input[,c(1,6:7,4,8,5)]
  tss.df[,3] = tss.df[,2] + 500
  tss.df[,2] = tss.df[,2] - 500

  #sum polymerase density for each position in window
  combined.plus.bw = load.bigWig('~/Adipogenesis/PRO_analysis_redo/dREG/plus_dREG_merged.bigWig')
  combined.minus.bw = load.bigWig('~/Adipogenesis/PRO_analysis_redo/dREG/minus_dREG_merged.bigWig')
  dat.x = bed6.step.probeQuery.bigWig(combined.plus.bw, combined.minus.bw, tss.df, step = 1, as.matrix = TRUE, op = "sum", follow.strand = FAL
  dat.x[is.na(dat.x)] <- 0
  dat.x.win = t(apply(dat.x, 1, function(x){rollapply(x, width = 50, FUN = mean, by = 1, by.column = FALSE,align = "left")}))
  index.max = apply(dat.x.win, 1, which.max)
  the.max = apply(dat.x.win, 1, max)

  #find highest value in window to use as pause summit and take a 50 bp window around it - this is the 'pause region'
  pause.df = tss.df
  pause.df[2][pause.df$strand == '-',] = pause.df[2][pause.df$strand == '-',] + index.max[pause.df$strand == '-']
  pause.df[2][pause.df$strand == '+',] = pause.df[2][pause.df$strand == '+',] + index.max[pause.df$strand == '+']
  pause.df[3] = pause.df[2] + 50
  #pause.df[3] = pause.df[2] + 25
  #pause.df[2] = pause.df[2] - 25

  #define 'body region' as end of pause region to the end of the gene (strand specific)
  body.df = bed.input[,c(1:4,8,5)]
  body.df[body.df$strand == '+',2] = pause.df[pause.df$strand == '+',3]+1
  body.df[body.df$strand == '-',3] = pause.df[pause.df$strand == '-',2]-1
```

```
  #measure polymerase density within pause and body regions (sum for pause, average for body)
  output.df=bed.input[,c(1:5,8:9)]
  wiggle.plus = load.bigWig(paste0('PRO_',time,'_plus_merged_normalized.bigWig'))
  wiggle.minus = load.bigWig(paste0('PRO_',time,'_minus_merged_normalized.bigWig'))
  output.df[,8] = bed6.region.bpQuery.bigWig(wiggle.plus, wiggle.minus, pause.df, op = "sum")
  output.df[,9] = bed6.region.bpQuery.bigWig(wiggle.plus, wiggle.minus, body.df, op = "avg")

  colnames(output.df)[c(8,9)] = c('pause_sum','body_avg')
  return(output.df)
}

load('final.pro.all.df.Rdata')

factors = c('AP1','CEBP','GR','KLF','SP','TWIST')
time.strings = c('0m','20m','40m','60m','120m','180m','240m')
times = c(0,20,40,60,120,180,240)

pause.region.summation = data.frame()
for(factor in factors) {
  print(factor)
  for(i in 1:length(times)) {
    time = time.strings[i]
    print(time)
    isolated.genes = read.table(paste0(factor,'.isolated.genes.txt'),sep='\t')[,1]

    #make TSS data frame for isolated factor genes
    bed.input = final.pro.all.df[final.pro.all.df$gene %in% isolated.genes,c(1:5,7)]
    bed.input[,7] = bed.input[,6] + 1
    bed.input[,8] = factor
    bed.input[,9] = times[i]
    colnames(bed.input) = c('chr', 'start', 'end', 'gene', 'strand', 'TSS','TSS+1','factor','time')

    df = bwplot.input.pause(bed.input,time)

    pause.region.summation = rbind(pause.region.summation,df)

  }
}
pause.region.summation$pause_index = pause.region.summation[,'pause_sum'] / pause.region.summation[,'body_avg']
save(pause.region.summation,file='pause.region.summation.Rdata')

system('mkdir bwplots')

time.pts = c(0,20,40,60,120,180,240)

start = c()
end = c()
comparisons = c()
for (i in 1:(length(time.pts)-1)) {
  for (j in (i+1):length(time.pts)) {
    start = append(start,time.pts[i])
    end = append(end,time.pts[j])
    comparisons = append(comparisons,paste0(time.pts[j],'.v.', time.pts[i]))
  }
}
comparisons.df = data.frame(comparisons,start,end)

colors.df = data.frame(factors = c('AP1','CEBP','GR','KLF','SP','TWIST'),
                       color = c('#ffcd30','#c469aa','#6aa3ce','#5caa62','#835b24','#fbae23'),
                       dir = c(rep('Activated',4),rep('Repressed',2)))

for(factor in colors.df$factors) {
  dir <- colors.df[colors.df$factors == factor,'dir']
  for(i in 1:nrow(comparisons.df)) {
    early = comparisons.df[i,'start']
    late = comparisons.df[i,'end']

    col1 = which(colnames(final.pro.all.df) == paste0('min.',early))
    col2 = which(colnames(final.pro.all.df) == paste0('min.',late))

    color <- colors.df[colors.df$factor == factor,'color']

    genes <- read.table(paste0(factor,'.isolated.genes.txt'),sep='\t')[,1]

    x <- final.pro.all.df[final.pro.all.df$gene %in% genes,c(col1,col2)]
    x$diff <- x[,2] - x[,1]
    x$temp <- 'Repressed'
    x[x$diff > 0,'temp'] <- 'Activated'
    #table(x$temp)
    genes <- sapply(strsplit(rownames(x[x$temp == dir,]),'_'),'[',2)

    df <- pause.region.summation[pause.region.summation$gene %in% genes,]

    plot.df = data.frame(gene = df[df$time == early,'gene'],
                         ratio = df[df$time == late,'pause_index'] / df[df$time == early,'pause_index'],
                         x = 1,
```

```
                          early = early,
                          late = late)

    pdf(paste0('bwplots/',factor,'_pause_index_ratio_',late,'.v.',early,'_bwplot.pdf'), width=2.5, height=3.4)

    trellis.par.set(box.umbrella = list(lty = 1, col="#93939380", lwd=2),
                    box.rectangle = list(col = '#93939380', lwd=1.6),
                    plot.symbol = list(col='#93939380', lwd=1.6, pch ='.'))

    print(bwplot(log(ratio, base = 2) ~ x, data = plot.df,
                 between=list(y=1.0, x = 1.0),
                 scales=list(x=list(draw=FALSE),relation="free",rot = 45, alternating=c(1,1,1,1),cex=1,font=1),
                 xlab = '',
                 main = "Pause Index (PI) Ratio",
                 ylab= substitute(paste('log'[2]*'',nn), list(nn=paste0('(',late,' min PI / ',early,' min PI)'))),
                 horizontal =FALSE,  col= 'black',
                 aspect = 2,
                 par.settings=list(par.xlab.text=list(cex=1.2,font=1),
                                   par.ylab.text=list(cex=1.2,font=1),
                                   par.main.text=list(cex=1.2, font=1),
                                   plot.symbol = list(col='black', lwd=1.6, pch =19, cex = 0.25)),
                 strip = function(..., which.panel, bg) {
                   #bg.col = c("#ce228e" ,"grey60", "#2290cf","grey90")
                   strip.default(..., which.panel = which.panel,
                                 bg = rep(bg.col, length = which.panel)[which.panel])
                 },
                 panel = function(..., box.ratio, col) {
                   panel.abline(h = 0, col = 'grey45', lty = 2)
                   panel.violin(..., col = color,
                                varwidth = FALSE, box.ratio = box.ratio, outer = FALSE)
                   panel.stripplot(..., col='#54545380', do.out=FALSE, jitter.data=TRUE, amount = 0.2, pch = 16)
                   panel.bwplot(..., pch = '|', do.out = FALSE)

                 }))
    dev.off()
  }
}
```

4.6 Odds ratio analyses

We measured the likelihood of gene activation / repression based on its proximity to one or multiple increased / decreased peaks. We use odds ratios to statistically test the enrichment of activated v. non-activated proximity to factor peaks. This analysis is Figure 6C-F.

```
library(vcd)
library(ggplot2)

load('final.atac.all.df.Rdata')
load('final.pro.all.df.Rdata')

system('wget https://github.com/guertinlab/adipogenesis/raw/master/Pipeline_PRO/odds.ratio.df.Rdata')
system('wget https://github.com/guertinlab/adipogenesis/raw/master/Pipeline_PRO/odds.ratio.repressed.df.Rdata')

load('odds.ratio.df.Rdata')
load('odds.ratio.repressed.df.Rdata')

odds.ratio.df <- z

x[x$ap == TRUE,'ap'] = 'AP1 Proximal'
x[x$ap == FALSE,'ap'] = 'Not AP1 Proximal'
x[x$gr == TRUE,'gr'] = 'GR Proximal'
x[x$gr == FALSE,'gr'] = 'Not GR Proximal'
x[x$cebp == TRUE,'cebp'] = 'CEBP Proximal'
x[x$cebp == FALSE,'cebp'] = 'Not CEBP Proximal'
x[x$klf == TRUE,'klf'] = 'KLF Proximal'
x[x$klf == FALSE,'klf'] = 'Not KLF Proximal'
x[x$dynamic == TRUE,'dynamic'] = 'Activated'
x[x$dynamic == FALSE,'dynamic'] = 'Not Activated'

rownames(x) = x[,1]
x = x[,-1]

factor.df <- data.frame(vec = 1:4,
                        factors = c('AP1','CEBP','GR','KLF'),
                        colors = c('#ffcd30','#c469aa','#6aa3ce','#5caa62'))

for(i in factor.df$vec) {
  for(j in factor.df$vec[-i]) {

    factor1 <- factor.df$factors[i]
    factor2 <- factor.df$factors[j]
    color <- factor.df[factor.df$factors == factor2,'colors']
```

```
    x$label = paste(x[,i],x[,5])

    result <- as.data.frame(prop.table(table(x[,j],x[,'label']),margin = 2)*100)
    colnames(result) = c('fill','x','y')

    result$x = factor(result$x,levels = c(paste('Not',factor1,'Proximal Not Activated'),
                                          paste('Not',factor1,'Proximal Activated'),
                                          paste(factor1,'Proximal Not Activated'),
                                          paste(factor1,'Proximal Activated')))

    result$fill = factor(result$fill,levels = c(paste('Not',factor2,'Proximal'),
                                                paste(factor2,'Proximal')))

    pdf(file = paste0(factor1,'.pos.v.',factor2,'.pdf'),width=3,height=3)
    print(
      ggplot(result, aes(x, y, fill = fill)) +
        geom_bar(position='stack',stat='Identity',color='black',width=0.8) +
        labs(x = '',y = 'Percent', fill = '') +
        scale_y_continuous(expand = c(0, 0)) +
        theme_classic() +
        theme(panel.grid.minor = element_blank(),
              axis.ticks = element_blank(),
              axis.text.x = element_blank(),
              axis.title.x = element_blank(),
              #axis.text.x = element_text(angle=40,size=4,hjust=.99,vjust=1,color='black',face='bold'),
              axis.text.y = element_text(size=14,face='bold',color='black'),
              axis.title.y = element_text(size=14,face='bold'),
              legend.text = element_text(size=8,face='bold'),
              legend.position="top",
              legend.justification='left') +
        scale_fill_manual(values = c('grey',color))
    )
    dev.off()
  }
}

for(i in factor.df$vec) {
  for(j in factor.df$vec[-i]) {

    factor1 <- factor.df$factors[i]
    factor2 <- factor.df$factors[j]
    color <- factor.df[factor.df$factors == factor2,'colors']

    x$label = paste(x[,i],x[,j])

    result <- prop.table(table(x[,'dynamic'],x[,'label']),margin = 2)*100
    labels <- colnames(result)
    result <- result[-2,]

    pdf(file = paste0('linked.',factor1,'.v.',factor2,'.pdf'),width=3,height=4)
    par(las=2)
    plot <- barplot(result,
                    col = color,
                    cex.names= 1,
                    legend.text = FALSE,
                    args.legend = list(x='top',
                                       inset=c(0,-0.3),
                                       bty='n',
                                       text.font = 2),
                    font.axis=2,
                    font.lab=2,
                    cex.axis = 1.1,
                    cex.lab = 1.25,
                    ylab = 'Percent Linked',
                    xaxt = 'n')
    text(cex=1.1, x=plot+0.1, y=-0.8, labels, xpd = T, pos = 2, srt=90, font = 2)
    dev.off()

  }
}

#Calculate odds ratios
x[,5] = factor(x[,5],levels=c('Not Activated','Activated'))
x[,1] = factor(x[,1],levels=c('Not AP1 Proximal','AP1 Proximal'))
x[,3] = factor(x[,3],levels=c('Not GR Proximal','GR Proximal'))

tab = table(x[,c(1,5,3)])
lor=oddsratio(tab,3)
print(exp(confint(lor)))

tab = table(x[,c(3,5,1)])
lor=oddsratio(tab,3)
print(exp(confint(lor)))

#Do the same for repressive factors
x <- odds.ratio.repressed.df
```

```
x[x$sp == TRUE,'sp'] = 'SP Proximal'
x[x$sp == FALSE,'sp'] = 'Not SP Proximal'
x[x$twist == TRUE,'twist'] = 'TWIST Proximal'
x[x$twist == FALSE,'twist'] = 'Not TWIST Proximal'
x[x$dynamic == TRUE,'dynamic'] = 'Repressed'
x[x$dynamic == FALSE,'dynamic'] = 'Not Repressed'

rownames(x) = x[,1]
x = x[,-1]

y <- x
x[,3] = factor(x[,3],levels=c('Not Repressed','Repressed'))
x[,1] = factor(x[,1],levels=c('Not SP Proximal','SP Proximal'))
x[,2] = factor(x[,2],levels=c('Not TWIST Proximal','TWIST Proximal'))

tab = table(x[,c(1,3,2)])
lor=oddsratio(tab,3)
print(exp(confint(lor)))

tab = table(x[,c(2,3,1)])
lor=oddsratio(tab,3)
print(exp(confint(lor)))
```

4.7 Attenuated edges

We identify *cis* and *trans* edges that are attenuated - meaning the move in the opposite direction after some time. For example, a peak that initially increases in accessibility but then decreases later in the time course is an example of an attenuated *trans* edge. These numbers are reported in Supplemental Figure S8A and B.

```
library(ggplot2)

load('trans.links.Rdata')
load('cis.links.Rdata')
load('final.atac.all.df.Rdata')
load('final.pro.all.df.Rdata')

time.pts = c(0,20,40,60,120,180,240)

factor.df = data.frame(factors = c('AP1','CEBP','GR','KLF','SP','TWIST'),
                       attenuated.peak.dir = c(rep('Decreased',4),rep('Increased',2)),
                       attenuated.gene.dir = c(rep('Repressed',4),rep('Activated',2)))

attenuated.trans.links = data.frame()
attenuated.cis.links = data.frame()
for(factor in factor.df$factors) {
  print(factor)
  attenuated.factor.links = data.frame()

  factor.attenuated.trans.links = trans.links[trans.links$source == factor,]
  factor.attenuated.cis.links = cis.links[cis.links$factor == factor,]

  #define attenuated direciton (ex. decreases if factor normally drives increases)
  peak.dir = factor.df[factor.df$factors == factor,'attenuated.peak.dir']
  gene.dir = factor.df[factor.df$factors == factor,'attenuated.gene.dir']

  #don't consider significant ecdf comparisons
  #cdf = read.table(paste0('ecdf/',factor,'_ecdf_pvalues.txt'), stringsAsFactors = FALSE)
  #cdf$activated.fdr = p.adjust(cdf[,4])
  #cdf$repressed.fdr = p.adjust(cdf[,5])
  #if(peak.dir == 'Increased') {
  #  strings = strsplit(cdf[cdf$activated.fdr < 0.01,]$V2,'response.')
  #  comparisons = unlist(strings)[2*(1:length(strings)-1)]
  #} else {
  #  strings = strsplit(cdf[cdf$repressed.fdr < 0.01,]$V2,'response.')
  #  comparisons = unlist(strings)[2*(1:length(strings)-1)]
  #}

  #if(length(comparisons) == 0) {
  #  print('no sig comparisons')
  #  next
  #}

  for(peak in unique(factor.attenuated.trans.links$target)) {

    #find significant comparisons and take early time
    potential.times =c()
    times = time.pts[time.pts > time.pts[which(time.pts == max(factor.attenuated.trans.links[factor.attenuated.trans.links$target == peak,'tim
    for(time in times) {
      colnums = intersect(grep('^response',colnames(final.atac.all.df)),grep(paste0('.v.',time),colnames(final.atac.all.df)))
      if(peak.dir %in% final.atac.all.df[rownames(final.atac.all.df) == peak,colnums]) {
```

```
        potential.times = append(potential.times,time)
      }
    }

    #for each potential time point see if accessibility changes by 10% between that point and the next
    for(time in potential.times) {
      i = which(time.pts == time)
      numbers = final.atac.all.df[rownames(final.atac.all.df) == peak,4:10]
      if(peak.dir == 'Increased' & numbers[,i+1] > 1.05*numbers[,i]) {
        attenuated.factor.links = rbind(attenuated.factor.links,c(factor,peak,time,peak.dir))
      } else if(peak.dir == 'Decreased' & numbers[,i+1] < 0.95*numbers[,i]) {
        attenuated.factor.links = rbind(attenuated.factor.links,c(factor,peak,time,peak.dir))
      }
    }
  }

  if(nrow(attenuated.factor.links) == 0) {
    next
  }

  colnames(attenuated.factor.links) = c('source','target','time','dir')
  attenuated.trans.links = rbind(attenuated.factor.links,attenuated.trans.links)

  #cis links
  attenuated.factor.cis.links = data.frame()
  temp = factor.attenuated.cis.links[factor.attenuated.cis.links$factor == factor,]
  for(gene in unique(temp$target)) {
    potential.times =c()
    times = time.pts[time.pts > time.pts[which(time.pts == max(temp[temp$target == gene,'time']))]]
    for(time in times) {
      colnums = intersect(grep('^response',colnames(final.pro.all.df)),grep(paste0('.v.',time),colnames(final.pro.all.df)))
      if(gene.dir %in% final.pro.all.df[final.pro.all.df$gene == gene,colnums]) {
        potential.times = append(potential.times,time)
      }
    }

    for(time in potential.times) {
      i = which(time.pts == time)
      numbers = final.pro.all.df[final.pro.all.df$gene == gene,9:15]

      if(gene.dir == 'Activated' & numbers[,i+1] > 1.05*numbers[,i]) {
        attenuated.factor.cis.links = rbind(attenuated.factor.cis.links,c(gene,time,gene.dir,factor))
      } else if(gene.dir == 'Repressed' & numbers[,i+1] < 0.95*numbers[,i]) {
        attenuated.factor.cis.links = rbind(attenuated.factor.cis.links,c(gene,time,gene.dir,factor))
      }
    }
  }

  if(nrow(attenuated.factor.cis.links) == 0) {
    next
  }

  colnames(attenuated.factor.cis.links) = c('target','time','dir','factor')
  attenuated.cis.links = rbind(attenuated.factor.cis.links,attenuated.cis.links)
}

save(attenuated.trans.links,file='attenuated.trans.links.Rdata')
save(attenuated.cis.links,file='attenuated.cis.links.Rdata')

#plot attenuated trans edges by factor ('inflection point')
factors = unique(attenuated.trans.links$source)

plot.df = data.frame()

for(factor in factors) {
  x = attenuated.trans.links[attenuated.trans.links$source == factor,]
  df = data.frame(peaks = unique(x$target),true.time = 0)
  for(peak in df$peaks) {
    times = unique(x[x$target == peak,]$time)
    true.time = ''
    for(time in times) {
      i = which(time.pts == time)
      numbers = final.atac.all.df[rownames(final.atac.all.df) == peak,4:10]
      if(factor %in% c('SP','TWIST') & numbers[,i+1] > 1.05*numbers[,i]) {
        df[df$peaks == peak,'true.time'] = time
        break
      } else if(factor %in% c('AP1','CEBP','GR','KLF') & numbers[,i+1] < 0.95*numbers[,i]) {
        df[df$peaks == peak,'true.time'] = time
        break
      }
    }
  }
  temp = data.frame(factor = factor,times = names(table(df$true.time)),num = as.vector(table(df$true.time)))
  plot.df = rbind(plot.df,temp)
}
plot.df$times = as.numeric(plot.df$times)
```

```
plot.df$num = as.numeric(plot.df$num)

pdf('number.attenuated.trans.edges.regulated.at.times.inflection.point.pdf',width=5,height=5)
print(
  ggplot(data = plot.df, aes(x = times,y = num,group = factor)) +
    geom_line(size = 1.5, aes(color = factor)) +
    geom_point(size = 2, aes(color = factor)) +
    xlim(0,121) +
    labs(x = 'Time (min)',
         y = 'Number of Attenuated Trans Edges',
         legend = 'Factor') +
    theme_minimal() +
    theme(axis.title = element_text(size = 14,face = 'bold'),
          axis.text = element_text(size = 12,face = 'bold',color = 'black'),
          legend.title = element_text(size = 14, face = 'bold'),
          legend.text = element_text(size = 12, face = 'bold')) +
    scale_color_manual(values = c('#ffcb31','#c469aa','#397fbb','#5caa62','#835b24','#faa51c'))
)
dev.off()

#plot attenuated cis edges by factor ('greatest change')
factors = unique(attenuated.cis.links$factor)

plot.df = data.frame()

for(factor in factors) {
  x = attenuated.cis.links[attenuated.cis.links$factor == factor,]
  df = data.frame(genes = unique(x$target),true.time = 0)
  for(gene in df$genes) {
    times = unique(x[x$target == gene,]$time)
    true.time = ''
    for(time in times) {
      i = which(time.pts == time)
      numbers = final.pro.all.df[final.pro.all.df$gene == gene,9:15]
      if(factor %in% c('SP','TWIST') & numbers[,i+1] > 1.05*numbers[,i]) {
        df[df$genes == gene,'true.time'] = time
        break
      } else if(factor %in% c('AP1','CEBP','GR','KLF') & numbers[,i+1] < 0.95*numbers[,i]) {
        df[df$genes == gene,'true.time'] = time
        break
      }
    }
  }
  temp = data.frame(factor = factor,times = names(table(df$true.time)),num = as.vector(table(df$true.time)))
  plot.df = rbind(plot.df,temp)
}
plot.df$times = as.numeric(plot.df$times)
plot.df$num = as.numeric(plot.df$num)

pdf('number.attenuated.cis.edges.regulated.at.times.inflection.point.pdf',width=5,height=5)
print(
  ggplot(data = plot.df, aes(x = times,y = num,group = factor)) +
    geom_line(size = 1.5, aes(color = factor)) +
    geom_point(size = 2, aes(color = factor)) +
    xlim(0,121) +
    labs(x = 'Time (min)',
         y = 'Number of Attenuated Cis Edges',
         legend = 'Factor') +
    theme_minimal() +
    theme(axis.title = element_text(size = 14,face = 'bold'),
          axis.text = element_text(size = 12,face = 'bold',color = 'black'),
          legend.title = element_text(size = 14, face = 'bold'),
          legend.text = element_text(size = 12, face = 'bold')) +
    scale_color_manual(values = c('#ffcb31','#c469aa','#397fbb','#5caa62','#835b24','#faa51c'))
)
dev.off()

#plot proportion of edges that are attenuated
factors = c('AP1','CEBP','GR','KLF','TWIST')
colors = c('#ffca31','#c568a9','#397fba','#246c36','#ffa500')

#trans
plot.df = data.frame(Factor = factors,perc.attenuated = 0,colors = colors)
for(factor in factors) {
  x = unique(attenuated.trans.links[attenuated.trans.links$source == factor,'target'])
  y = unique(trans.links[trans.links$source == factor,'target'])
  plot.df[plot.df$Factor==factor,'perc.attenuated'] = 100*(1 - (length(setdiff(y,x)) / length(y)))
}
plot.df$Factor = factor(plot.df$Factor,levels = plot.df$Factor)

pdf('perc.attenuated.trans.edges.pdf',width=5,height=5)
print(
  ggplot(plot.df,aes(x = Factor,y = perc.attenuated,fill=Factor)) +
    geom_bar(stat='identity',color='black') +
    labs(y = '% trans Edges That Are Attenuated',x='') +
    theme_minimal() +
```

```
    theme(panel.grid.minor = element_blank(),
          axis.text.x = element_text(angle=45,size=14,color='black',face='bold'),
          axis.text.y = element_text(size=12,face='bold',color='black'),
          axis.title.y = element_text(size=14,face='bold'),
          legend.position = "none",
          axis.ticks = element_blank()) +
    scale_fill_manual(values=plot.df$colors)
)
dev.off()

#cis
plot.df = data.frame(Factor = factors,perc.attenuated = 0,colors = colors)
for(factor in factors) {
  x = unique(attenuated.cis.links[attenuated.cis.links$factor == factor,'target'])
  y = unique(cis.links[cis.links$factor == factor,'target'])
  plot.df[plot.df$Factor==factor,'perc.attenuated'] = 100*(1 - (length(setdiff(y,x)) / length(y)))
}
plot.df$Factor = factor(plot.df$Factor,levels = plot.df$Factor)

pdf('perc.attenuated.cis.edges.pdf',width=5,height=5)
print(
  ggplot(plot.df,aes(x = Factor,y = perc.attenuated,fill=Factor)) +
    geom_bar(stat='identity',color='black') +
    labs(y = '% cis Edges That Are Attenuated',x='') +
    theme_minimal() +
    theme(panel.grid.minor = element_blank(),
          axis.text.x = element_text(angle=45,size=14,color='black',face='bold'),
          axis.text.y = element_text(size=12,face='bold',color='black'),
          axis.title.y = element_text(size=14,face='bold'),
          legend.position = "none",
          axis.ticks = element_blank()) +
    scale_fill_manual(values=plot.df$colors)
)
dev.off()
```

4.8 Time networks

We count the number of peaks and genes regulated by individual factor families - these numbers form the 'wedge plots' in Figure 7E and Supplemental Figure S8D.

```
load('tf.genes.Rdata')
load('attenuated.cis.links.Rdata')
load('attenuated.trans.links.Rdata')
load('trans.links.Rdata')
load('cis.links.Rdata')

tf.genes$name = ''
tf.genes[tf.genes$fam == 1,]$name = 'AP1'
tf.genes[tf.genes$fam == 2,]$name = 'bHLH'
tf.genes[tf.genes$fam == 3,]$name = 'GR'
tf.genes[tf.genes$fam == 4,]$name = 'Bhlha15'
tf.genes[tf.genes$fam == 5,]$name = 'CEBP'
tf.genes[tf.genes$fam == 6,]$name = 'CTCF'
tf.genes[tf.genes$fam == 7,]$name = 'SP'
tf.genes[tf.genes$fam == 8,]$name = 'ELK/ETV'
tf.genes[tf.genes$fam == 9,]$name = 'ZBTB33'
tf.genes[tf.genes$fam == 10,]$name = 'Maz'
tf.genes[tf.genes$fam == 11,]$name = 'NFI'
tf.genes[tf.genes$fam == 12,]$name = 'NFY'
tf.genes[tf.genes$fam == 13,]$name = 'NRF'
#tf.genes[tf.genes$fam == 14,]$name = 'Nur77'
tf.genes[tf.genes$fam == 15,]$name = 'STAT'
tf.genes[tf.genes$fam == 16,]$name = 'AP2'
tf.genes[tf.genes$fam == 17,]$name = 'TEAD'
tf.genes[tf.genes$fam == 18,]$name = 'TWIST'
tf.genes[tf.genes$fam == 19,]$name = 'ZNF263'
tf.genes = tf.genes[-grep('^Egr',tf.genes$factors),]
tf.genes[grep('Klf',tf.genes$factors),]$name = 'KLF'
tf.genes = tf.genes[tf.genes$name %in% c('AP1','GR','CEBP','KLF','SP','TWIST'),]
#

time.pts = c(0,20,40,60,120,180,240)
factors = unique(trans.links$source)

numbers = data.frame(factor = rep(factors,each=7),
                     time = rep(time.pts,6),
                     total.num.peaks = 0,
                     peaks.carryover = 0,
                     total.num.genes = 0,
                     genes.carryover = 0)

for(factor in factors) {
```

```
  factor.trans.links = trans.links[trans.links$source == factor,]
  factor.cis.links = cis.links[cis.links$factor == factor,]
  attenuated.factor.trans.links = attenuated.trans.links[attenuated.trans.links$source == factor,]
  attenuated.factor.cis.links = attenuated.cis.links[attenuated.cis.links$source == factor,]
  factor.tf.genes <- c()

  for(i in 1:length(time.pts)) {
    time = time.pts[i]
    next.time = time.pts[i+1]
    numbers[numbers$factor == factor & numbers$time == time,'total.num.peaks'] = nrow(factor.trans.links[factor.trans.links$time == time,])
    numbers[numbers$factor == factor & numbers$time == time,'peaks.carryover'] = length(intersect(factor.trans.links[factor.trans.links$time =
    numbers[numbers$factor == factor & numbers$time == time,'total.num.genes'] = length(unique(factor.cis.links[factor.cis.links$time == time,
    numbers[numbers$factor == factor & numbers$time == time,'genes.carryover'] = length(intersect(unique(factor.cis.links[factor.cis.links$tim

    genes = unique(factor.cis.links[factor.cis.links$time == time,'target'])
    if(length(intersect(genes,tf.genes$factors)) != 0) {
      factor.tf.genes <- unique(append(factor.tf.genes,intersect(genes,tf.genes$factors)))
    }
    #if(length(intersect(genes,tf.genes$factors)) != 0) {
    #  print(factor)
    #  print(time)
    #  print(tf.genes[tf.genes$factors %in% genes,])
    #}
  }
  print(factor)
  print(table(tf.genes[tf.genes$factors %in% factor.tf.genes,'name']))
}

save(numbers,file='numbers.Rdata')
```

5 Compartment modeling

Compartment modeling is described in the vignette found at `https://github.com/guertinlab/compartment_model`. Please visit for code and an extensive breakdown of the methodology. These analyses can be found in Figure 5 and Supplemental Figure S5.

6 shTwist2 RNA-seq analysis

6.1 Download .fastq files

First we download the .fastq files onto Rivanna from GEO (GSE219051) as with the ATAC-seq and PRO-seq data. Again, we submit a .slurm script for each sample and download all the files in parallel. header.txt:

```
#!/bin/bash
#SBATCH -n 1
#SBATCH -t 12:00:00
#SBATCH -p standard
#SBATCH -A janeslab
```

script for downloading .fastq files:

```
cd /scratch/abd3x/RNAseq

wget https://github.com/guertinlab/adipogenesis/raw/master/RNAseq/misc_scripts/RNA_SRA_table.txt
wget https://github.com/guertinlab/adipogenesis/raw/master/Pipeline_ATAC/misc_scripts/header.txt

echo 'Downloading .fastq'

cat RNA_SRA_table.txt | while read line
do
sra=$(echo $line | awk '{print $1}')
treat=$(echo $line | awk '{print $2}')
time=$(echo $line | awk '{print $3}')
rep=$(echo $line | awk '{print $4}')
echo '#SBATCH -o download.'$treat'.'$time'.rep'$rep'.out' > temp.txt
echo 'module load sratoolkit/2.10.5' > temp2.txt
echo 'fasterq-dump -3 '$sra' -o RNA_'${treat}'_'${time}'_rep'$rep > temp3.txt
echo 'echo DONE' > temp4.txt
cat header.txt temp.txt temp2.txt temp3.txt temp4.txt > download.$treat.${time}.rep$rep.slurm
sbatch download.$treat.${time}.rep$rep.slurm
rm temp.txt
rm temp2.txt
rm temp3.txt
rm temp4.txt
done
```

6.2 Aligning samples to the mouse genome

As with the ATAC and PRO data we submit the samples to align to the genome as individual slurm jobs. This time we will align to the most current mouse genome assembly (mm39). There are stome steps before alignment including making the mm39 hisat2 assembly and extracting splice sites from the latest .gtf. We will also extract gene lengths from the .gtf file, which necessitates downloading some python software. These steps take a while so it is best to submit it as a job.
install_gtftools.sh

```
module purge
module load bioconda/py3.8
conda create -n myenv3 -c bioconda gtftools

python3 -m pip install --user --upgrade gtftools
cp /home/abd3x/.local/bin/gtftools /home/abd3x/bin/
```

pre_align.slurm

```
#!/bin/bash
#SBATCH -n 1
#SBATCH -t 96:00:00
#SBATCH -p standard
#SBATCH -A janeslab
#SBATCH -o prealign.out

wget https://raw.githubusercontent.com/guertinlab/adipogenesis/master/RNAseq/hisat2_extract_splice_sites.py

module load gcc/7.1.0  openmpi/3.1.4 python/2.7
wget https://ftp.ebi.ac.uk/pub/databases/gencode/Gencode_mouse/release_M30/gencode.vM30.annotation.gtf.gz
gunzip gencode.vM30.annotation.gtf.gz
python hisat2_extract_splice_sites.py gencode.vM30.annotation.gtf > gencode.vM30.splice.txt

conda activate myenv3
HSA_GTF=gencode.vM30.annotation.gtf
grep -v '#' $HSA_GTF | awk '{OFS="\t"} $1=1' >> $HSA_GTF.tmp
gtftools -l $HSA_GTF.genelength $HSA_GTF.tmp

module purge
module load gcc/9.2.0 hisat2/2.1.0

wget https://hgdownload.soe.ucsc.edu/goldenPath/mm39/bigZips/mm39.fa.gz
gunzip mm39.fa.gz
hisat2-build mm39.fa mm39
```

align_footer.txt

```
cd /scratch/abd3x/RNAseq

echo Starting Alignment
module load gcc/9.2.0 hisat2/2.1.0 samtools/1.10

hisat2 -x mm39 --known-splicesite-infile gencode.vM30.splice.txt \
-1 ${name}_1.fastq -2 ${name}_2.fastq | samtools view -b - | \
samtools sort -n - -o $name.sorted.bam

echo Sorting and Removing Junk

samtools sort $name.sorted.bam -o $name.sorted.bam
samtools index $name.sorted.bam
samtools view -bh $name.sorted.bam \
chr1 chr2 chr3 chr4 chr5 chr6 chr7 chr8 chr9 chr10 chr11 chr12 \
chr13 chr14 chr15 chr16 chr17 chr18 chr19 chrX chrY chrM > $name.noJunk.bam

echo Removing Duplicate Reads

samtools sort -n $name.noJunk.bam | samtools fixmate -m - - | \
samtools sort - | samtools markdup -rs - $name.noDups.bam

echo Starting Sorting

module purge
module load bioconda/py3.8 samtools/1.10

samtools index $name.noDups.bam
samtools view -bf 1 $name.noDups.bam | htseq-count -f bam -r pos \
--stranded=yes - gencode.vM30.annotation.gtf > $name.gene.counts.txt
samtools view -bf 1 $name.noDups.bam | htseq-count -f bam -r pos \
--stranded=reverse $name.noDups.bam gencode.vM30.annotation.gtf > $name.reverse.gene.counts.txt

echo 'DONE'
```

align_script.sh

```
for fq in *_1.fq.gz
do
name=$(echo $fq | awk -F"_1.fastq" '{print $1}')
echo $name
echo '#SBATCH -o' $name'.out' > temp.txt
echo 'name='$name > temp2.txt
cat header.txt temp.txt temp2.txt align_footer.txt > align.$name.slurm

#sbatch $name.slurm
rm temp.txt
rm temp2.txt

done
```

6.3 Create read counts table and plot PCA

We load our RNA-seq reads into R and build a raw read counts table. We also calculate and save DESeq2-normalized counts as well as transcripts per million (TPM). TPM is the best way to illustrate transcript abundance.

```
setwd('/scratch/abd3x/RNAseq')

ensembl.all = read.table('gencode.vM30.annotation.gtf', sep='\t', header =F)
ensembl.gene.names = data.frame(sapply(strsplit(
  sapply(strsplit(as.character(ensembl.all[,9]),'gene_name '), "[", 2), ";"), "[", 1))
ensembl.id.names = data.frame(sapply(strsplit(
  sapply(strsplit(as.character(ensembl.all[,9]),'gene_id '), "[", 2), ";"), "[", 1))

ensembl.code = cbind(ensembl.gene.names, ensembl.id.names)
ensembl.code = ensembl.code[!duplicated(ensembl.code[,2]),]
colnames(ensembl.code) = c('gene', 'id')
save(ensembl.code, file = "ensembl.code.Rdata")

all.counts = data.frame(row.names =
                          read.table('RNA_shControl_0h_rep1.reverse.gene.counts.txt')[,1])
for(bed in Sys.glob(file.path('*.reverse.gene.counts.txt'))) {
  name=strsplit(strsplit(bed,'.reverse.gene.counts.txt')[[1]][1],'RNA_')[[1]][2]
  print(name)
  x = read.table(bed,row.names=1,col.names=c('gene',name))
  all.counts = cbind(all.counts,x)
}
merged.counts <- all.counts[-((nrow(all.counts)-4):nrow(all.counts)),]

#replace Ensembl IDs with gene names in 'merged.counts' row names
merged.counts = merge(merged.counts, ensembl.code, by.x="row.names",by.y=2, all.x=T)
rownames(merged.counts) <- make.names(merged.counts$gene, unique=TRUE)
merged.counts <- merged.counts[,-c(1,which(colnames(merged.counts) == 'gene'))]
merged.counts <- merged.counts[-which(rowSums(merged.counts)==0),]
save(merged.counts, file="merged.counts.Rdata")


#Generate normalized counts object
unt=8
trt=16
sample.conditions = factor(c(rep("untreated",unt), rep("treated",trt)), levels=c("untreated","treated"))

deseq.counts.table = DESeqDataSetFromMatrix(merged.counts,
                                            as.data.frame(sample.conditions), ~ sample.conditions)

dds = DESeq(deseq.counts.table)

normalized.counts = counts(dds, normalized=TRUE)
save(normalized.counts,file='normalized.counts.Rdata')

#Generate TPM object
lengths <- read.table('gencode.vM30.annotation.gtf.genelength',sep='\t',header=T)
lengths <- merge(ensembl.code,lengths,by.x = 2,by.y=1)
rownames(lengths) <- make.names(lengths$gene,unique=TRUE)

merged.counts.lengths <- merge(merged.counts,
                               lengths,
                               by=0, all.x=T)

rownames(merged.counts.lengths) <- merged.counts.lengths[,1]
merged.counts.lengths <- merged.counts.lengths[,-1]
merged.counts.lengths[,1:24] <- merged.counts.lengths[,1:24] / (merged.counts.lengths$longest_isoform/1000)
scaling.factors <- colSums(merged.counts.lengths[,1:24])/1000000

for(i in 1:24) {
  merged.counts.lengths[,i] <- merged.counts.lengths[,i] / scaling.factors[i]
}
```

```
tpm.counts <- merged.counts.lengths[,1:24]
save(tpm.counts,file='tpm.counts.Rdata')
```

We plot Twist2 expression to illustrate knockdown and activation over time course. This is Figure 8A.

```
colors = c('light grey','light blue','blue')

plot.df <- data.frame(factor = rep(genes,12),
                      time = rep(c(0,1,2,4),each=length(genes)),
                      col1 = rep(seq(1,23,2),each=length(genes)),
                      col2 = rep(seq(2,24,2),each=length(genes)),
                      type = rep(c('shControl','shTwist2v1','shTwist2v2'),each=(4*length(genes))),
                      expression = 0)
for(i in 1:nrow(plot.df)) {
  plot.df[i,'expression'] <- mean(c(normalized.counts[plot.df$factor[i],plot.df$col1[i]],
                                    normalized.counts[plot.df$factor[i],plot.df$col2[i]]))
  y.label <- 'DESeq-Normalized Read Counts'
}

plot.df$type <- factor(plot.df$type,levels=c('shControl','shTwist2v1','shTwist2v2'))

pdf(file = 'Twist2.expression.figure.pdf',width=4,height=4)
print(
  ggplot(plot.df, aes(x=time, y=expression,color=type,group=type)) +
    geom_point(size=2) +
    geom_line(size=1) +
    #geom_violin(alpha=0.5) +
    #geom_jitter(shape=16, col = 'light grey', position=position_jitter(0.2)) +
    theme_minimal() +
    labs(y = y.label,
         x = 'Hours',
         title='Twist2 Expression',
         color=NULL) +
    #color = 'Near',alpha='Magnitude Total \n ATAC Change') +
    theme(
      #panel.grSp.minor = element_blank(),
      plot.title = element_text(size=12,face='bold',hjust = 0.5),
      axis.ticks = element_blank(),
      axis.text.y = element_text(size=11,color='black',face='bold'),
      axis.text.x = element_text(size=11,color='black',face='bold'),
      axis.title = element_text(size=12,color='black',face='bold'),
      legend.text = element_text(size=10,color='black',face='bold'),
      legend.title = element_text(size=11,color='black',face='bold')) +
    coord_cartesian(ylim = c(0, 1.05*max(plot.df$expression))) +
    scale_color_manual(values=colors)
)
dev.off()
```

As with the ATAC data, we perform PCA to assess sample similarity.

```
library(DESeq2)
library(lattice)
library(ggplot2)

plotPCAlattice <- function(df, file = 'PCA_lattice.pdf') {
  perVar = round(100 * attr(df, "percentVar"))
  df = data.frame(cbind(df, sapply(strsplit(as.character(df$name), '_rep'), '[', 1)))
  colnames(df) = c(colnames(df)[1:(ncol(df)-1)], 'unique_condition')
  print(df)
  #get colors and take away the hex transparency
  color.x = substring(rainbow(length(unique(df$unique_condition))), 1,7)
  df$color = NA
  df$alpha.x = NA
  df$alpha.y = NA
  df$colpal = NA
  for (i in 1:length(unique(df$unique_condition))) {
    df[df$unique_condition == unique(df$unique_condition)[[i]],]$color = color.x[i]
    #gives replicates for unique condition
    reps_col<- df[df$unique_condition == unique(df$unique_condition)[[i]],]
    #gives number of replicates in unique condition
    replicates.x = nrow(reps_col)
    alx <- rev(seq(0.2, 1, length.out = replicates.x))
    #count transparency(alx), convert alx to hex(aly), combain color and transparency(cp)
    for(rep in 1:replicates.x) {
      na <- reps_col[rep, ]$name
      df[df$name == na, ]$alpha.x = alx[rep]
      aly = as.hexmode(round(alx * 255))
      df[df$name == na, ]$alpha.y = aly[rep]
      cp = paste0(color.x[i], aly)
      df[df$name == na, ]$colpal = cp[rep]
      #print(df)
    }
  }
  colpal = df$colpal
```

```
  df$name = gsub('_', ' ', df$name)
  df$name <- factor(df$name, levels=df$name, order=TRUE)
  pdf(file, width=6, height=6, useDingbats=FALSE)
  print(xyplot(PC2 ~ PC1, groups = name, data=df,
               xlab = paste('PC1: ', perVar[1], '% variance', sep = ''),
               ylab = paste('PC2: ', perVar[2], '% variance', sep = ''),
               par.settings = list(superpose.symbol = list(pch = c(20), col=colpal)),
               pch = 20, cex = 1.7,
               auto.key = TRUE,
               col = colpal))
  dev.off()
}

setwd('/scratch/abd3x/RNAseq')

unt=8
trt=16
sample.conditions = factor(c(rep("untreated",unt), rep("treated",trt)), levels=c("untreated","treated"))
#convert counts table to a DESeq object
deseq.counts.table = DESeqDataSetFromMatrix(merged.counts, DataFrame(sample.conditions), ~ sample.conditions);
colData(deseq.counts.table)$condition<-factor(colData(deseq.counts.table)$sample.conditions, levels=c('untreated','treated'));
dds = DESeq(deseq.counts.table)
#take a log2 transformation of all the read counts
rld_HH = rlogTransformation(dds)
#calculate principal components
pca.dat = plotPCA(rld_HH, intgroup="condition", returnData=TRUE)
percentVar = round(100 * attr(pca.dat, "percentVar"))
#plot PCA
plotPCAlattice(pca.dat, file = 'PCA_shTwist2_RNAseq.pdf')
```

6.4 Identifying differentially expressed genes across different comparisons

We can perform comparisons across time (ex. shControl 2 v. 0 hours) and conditions (ex. 1 hr shTwist2v1 v. shControl).

```
meta.data <- data.frame(sample = rep(c('shControl','shTwist2v1','shTwist2v2'),each=8),
                        time = rep(c(0,1,2,4),each=2,times=3),
                        col = c(1:24))
meta.data$label <- paste0(meta.data$sample,'.',meta.data$time)

#first do comparisons of time points
times <- c(0,1,2,4)

comparisons = c()
for (i in 1:(length(times)-1)) {
  for (j in (i+1):length(times)) {
    comparisons = append(comparisons,paste0(times[j],'.v.', times[i]))
  }
}

size.factors <- estimateSizeFactorsForMatrix(merged.counts)
thresh <- 0.1
results.df <- data.frame(row.names = rownames(merged.counts))
for(sample in unique(meta.data$sample)) {
  print(sample)
  for(comparison in comparisons) {
    print(comparison)
    end <- strsplit(comparison,'.v.')[[1]][1]
    start <- strsplit(comparison,'.v.')[[1]][2]
    end.cols <- as.vector(meta.data[meta.data$sample == sample & meta.data$time == end,'col'])
    start.cols <- as.vector(meta.data[meta.data$sample == sample & meta.data$time == start,'col'])
    merged.counts.small = merged.counts[,c(start.cols,end.cols)]
    #establish the number of replicates per condition
    unt = 2
    trt = 2
    #the following commands create a DESeq object, calculate average expression and fold change between different conditions,
    #and run t-tests for each gene
    sample.conditions = factor(c(rep("untreated",unt), rep("treated",trt)), levels=c("untreated","treated"))
    mm.deseq.counts.table = DESeqDataSetFromMatrix(merged.counts.small, DataFrame(sample.conditions), ~ sample.conditions)
    mm.atac = mm.deseq.counts.table

    sizeFactors(mm.atac) = size.factors[colnames(merged.counts.small)]
    mm.atac = estimateDispersions(mm.atac)
    mm.atac = nbinomWaldTest(mm.atac)
    #resultsNames(mm.atac)
    res.mm.atac = results(mm.atac)
    #res.mm.atac = lfcShrink(mm.atac,coef="sample.conditions_treated_vs_untreated")
    results <- as.data.frame(res.mm.atac)
    results <- results[,c(1,2,6)]
    results$FoldChange <- 2^results[,2]
    results$Response <- 'Unchanged'
    if(nrow(results[results$padj < thresh & !is.na(results$padj) & results$log2FoldChange < 0,]) != 0) {
```

```
      results[results$padj < thresh & !is.na(results$padj) & results$log2FoldChange < 0,]$Response <- 'Repressed'
    }
    if(nrow(results[results$padj < thresh & !is.na(results$padj) & results$log2FoldChange > 0,]) != 0) {
      results[results$padj < thresh & !is.na(results$padj) & results$log2FoldChange > 0,]$Response <- 'Activated'
    }
    colnames(results) <- paste0(colnames(results),'.',sample,'.',comparison)
    results.df <- merge(results.df,results,by='row.names',all=T)
    rownames(results.df) <- results.df$Row.names
    results.df <- results.df[,-which(colnames(results.df) == 'Row.names')]
  }
}

#now do comparisons of conditions
comparisons <- c('shTwist2v1.v.shControl',
                 'shTwist2v2.v.shControl',
                 'shTwist2v1.v.shTwist2v2')

for(time in times) {
  for(comparison in comparisons) {
    sample <- strsplit(comparison,'.v.')[[1]][1]
    sample2 <- strsplit(comparison,'.v.')[[1]][2]
    print(comparison)
    end.cols <- as.vector(meta.data[meta.data$sample == sample & meta.data$time == time,'col'])
    start.cols <- as.vector(meta.data[meta.data$sample == sample2 & meta.data$time == time,'col'])
    merged.counts.small = merged.counts[,c(start.cols,end.cols)]
    #establish the number of replicates per condition
    unt = 2
    trt = 2
    #the following commands create a DESeq object, calculate average expression and fold change between different conditions,
    #and run t-tests for each gene
    sample.conditions = factor(c(rep("untreated",unt), rep("treated",trt)), levels=c("untreated","treated"))
    mm.deseq.counts.table = DESeqDataSetFromMatrix(merged.counts.small, DataFrame(sample.conditions), ~ sample.conditions)
    mm.atac = mm.deseq.counts.table
    #atac.size.factors = estimateSizeFactorsForMatrix(merged.counts.small)
    sizeFactors(mm.atac) = size.factors[colnames(merged.counts.small)]
    mm.atac = estimateDispersions(mm.atac)
    mm.atac = nbinomWaldTest(mm.atac)
    res.mm.atac = results(mm.atac)
    #res.mm.atac = lfcShrink(mm.atac,coef="sample.conditions_treated_vs_untreated")
    results <- as.data.frame(res.mm.atac)
    results <- results[,c(1,2,6)]
    results$FoldChange <- 2^results[,2]
    results$Response <- 'Unchanged'
    if(nrow(results[results$padj < thresh & !is.na(results$padj) & results$log2FoldChange < 0,]) != 0) {
      results[results$padj < thresh & !is.na(results$padj) & results$log2FoldChange < 0,]$Response <- 'Repressed'
    }
    if(nrow(results[results$padj < thresh & !is.na(results$padj) & results$log2FoldChange > 0,]) != 0) {
      results[results$padj < thresh & !is.na(results$padj) & results$log2FoldChange > 0,]$Response <- 'Activated'
    }

    colnames(results) <- paste0(colnames(results),'.',time,'.',sample,'.v.',sample2)
    results.df <- merge(results.df,results,by='row.names',all=T)
    rownames(results.df) <- results.df$Row.names
    results.df <- results.df[,-which(colnames(results.df) == 'Row.names')]
  }
}
save(results.df,file=paste0('results.df.',thresh,'.Rdata'))
```

6.5 Exploring effect of chronic Twist2 depletion on basal transcription of predicted target genes

We want to evaluate how Twist2 knockdown affects predicted target genes. We take two orthogonal approaches to this question. The first is to observe the transcription of target genes at baseline following Twist2 depletion. If Twist2 acts as a repressor then we expect target genes to increase in transcription in the knockdown compared to the control. This difference should be greatest before addition of the adipogenic cocktail. Thi is Figure 8B.

```
load('cis.links.Rdata')
load('results.df.0.1.Rdata')
all.twist.genes <- cis.links[cis.links$factor == 'TWIST','target']

x <- data.frame(gene = unique(all.twist.genes[all.twist.genes %in% rownames(results.df)]),
                comparison = '',
                shC.fc = 0,
                kd.fc = 0,
                class = 'Both')

query.colnames <- colnames(results.df)[grep('Response.shControl[.]',colnames(results.df))]
query.colnames <- append(query.colnames,colnames(results.df)[grep('Response.shTwist2v1[.]',colnames(results.df))])
```

```
for(i in 1:nrow(x)) {
  gene <- x$gene[i]

  if(any(results.df[gene,query.colnames] == 'Repressed')) {
    colnames <- query.colnames[which(results.df[gene,query.colnames] == 'Repressed')]

    conditions <- sapply(strsplit(colnames, '[.]'), '[', 2)
    if(!'shControl' %in% conditions) {
      x$class[i] <- 'Just shTwist2'
    } else if (!'sh84' %in% conditions) {
      x$class[i] <- 'Just shControl'
    }

    if(length(colnames) > 1) {
      comparisons = sapply(strsplit(colnames, 'Response[.]'), '[', 2)
      temp <- comparisons[which.min(results.df[rownames(results.df) == gene,paste0('log2FoldChange.',comparisons)])]
      comparison <- paste0(sapply(strsplit(temp, '[.]'), '[', 2),
                           '.v.',
                           sapply(strsplit(temp, '[.]'), '[', 4))
    } else {
      comparison <- paste0(sapply(strsplit(colnames, '[.]'), '[', 3),
                           '.v.',
                           sapply(strsplit(colnames, '[.]'), '[', 5))
    }
    x$comparison[i] <- comparison

    x$shC.fc[i] <- results.df[rownames(results.df) == gene,paste0('FoldChange.shControl.',comparison)]
    x$kd.fc[i] <- results.df[rownames(results.df) == gene,paste0('FoldChange.shTwist2v1,.',comparison)]

  } else {
    x$comparison[i] <- 'REMOVE'
  }
}
x <- x[x$comparison != 'REMOVE',]

x$class <- factor(x$class,levels = c('Just shControl','Just shTwist2','Both'))

times <- c(0,1,2,4)

df <- data.frame(time = rep(c(0,1,2,4),3),
                 repressed = c(rep(TRUE,4),rep(FALSE,8)),
                 twist.linked = c(rep(TRUE,8),rep(FALSE,4)),
                 num.genes.inc = 0,
                 num.genes.dec = 0)

for(time in times) {

  #all repressed genes in Twist links

  plot.df <- results.df[rownames(results.df) %in% x$gene,grep(paste0(time,'.shTwist2v1.v.shControl'),colnames(results.df))]
  colnames(plot.df) <- c('baseMean','log2FC','padj','FC','Response')
  plot.df <- rbind(plot.df[plot.df$Response == 'Unchanged',],plot.df[plot.df$Response != 'Unchanged',])

  df[df$time == time & df$repressed == TRUE &
       df$twist.linked == TRUE,'num.genes.inc'] <- as.numeric(table(plot.df$log2FC > 0)[2])
  df[df$time == time & df$repressed == TRUE &
       df$twist.linked == TRUE,'num.genes.dec'] <- as.numeric(table(plot.df$log2FC > 0)[1])

  pdf(file=paste0('ma.plot.',time,'.shTwist2v1.v.shControl.twist.linked.all.repressed.pdf'),width=4,height=3)

  print(
    ggplot(plot.df, aes(x=log(baseMean,10), y=log2FC)) +
      geom_point(size=1,alpha=1,color='light grey') +
      #geom_abline(intercept = 0, slope = 1,color='red') +
      geom_hline(yintercept=0, color = "red") +
      #geom_violin(alpha=0.5) +
      #geom_jitter(shape=16, col = 'light grey', position=position_jitter(0.2)) +
      theme_minimal() +
      labs(y = 'log2FC',
           x = 'log10baseMean',
           title='All Repressed Genes in TWIST cis.links ',time,' shTwist2v1.v.shC') +
      #color = 'Near',alpha='Magnitude Total \n ATAC Change') +
      theme(
        #panel.grid.minor = element_blank(),
        plot.title = element_text(size=12,face='bold',hjust = 0.5),
        axis.ticks = element_blank(),
        axis.text.y = element_text(size=11,color='black',face='bold'),
        axis.text.x = element_text(size=11,color='black',face='bold'),
        axis.title = element_text(size=12,color='black',face='bold'),
        legend.text = element_text(size=10,color='black',face='bold'),
        legend.title = element_text(size=11,color='black',face='bold'))
  )

  dev.off()

}
```

The other approach is to compare the change in gene expression between the control and factor knockdown samples. Because the relative activation of Twist2 is greater in the knockdown samples we expect a relatively greater repression of target genes in the knockdown. To test this, we plot the fold change in expression of genes in the Twist2 knockdown versus the control knockdown. Genes that conform to our hypothesis fall below the 1:1 line. This is Figure 8C.

```
load('cis.links.Rdata')
load('results.df.0.1.Rdata')
all.twist.genes <- cis.links[cis.links$factor == 'TWIST','target']
print('Repressed in Twist links')
x <- data.frame(gene = unique(all.twist.genes[all.twist.genes %in% rownames(results.df)]),
                comparison = '',
                shC.fc = 0,
                kd.fc = 0,
                class = 'Both')

query.colnames <- colnames(results.df)[grep('Response.shControl[.]',colnames(results.df))]
query.colnames <- append(query.colnames,colnames(results.df)[grep('Response.shTwist2v1[.]',colnames(results.df))])

for(i in 1:nrow(x)) {
  gene <- x$gene[i]

  if(any(results.df[gene,query.colnames] == 'Repressed')) {
    colnames <- query.colnames[which(results.df[gene,query.colnames] == 'Repressed')]

    conditions <- sapply(strsplit(colnames, '[.]'), '[', 2)
    if(!'shC' %in% conditions) {
      x$class[i] <- 'Only shTwist2'
    } else if (!'sh84' %in% conditions) {
      x$class[i] <- 'Only shControl'
    }

    if(length(colnames) > 1) {
      comparisons = sapply(strsplit(colnames, 'Response[.]'), '[', 2)
      temp <- comparisons[which.min(results.df[rownames(results.df) == gene,paste0('log2FoldChange.',comparisons)])]
      comparison <- paste0(sapply(strsplit(temp, '[.]'), '[', 2),
                           '.v.',
                           sapply(strsplit(temp, '[.]'), '[', 4))
    } else {
      comparison <- paste0(sapply(strsplit(colnames, '[.]'), '[', 3),
                           '.v.',
                           sapply(strsplit(colnames, '[.]'), '[', 5))
    }
    x$comparison[i] <- comparison

    x$shC.fc[i] <- results.df[rownames(results.df) == gene,paste0('FoldChange.shC.',comparison)]
    x$kd.fc[i] <- results.df[rownames(results.df) == gene,paste0('FoldChange.',sample,'.',comparison)]

  } else {
    x$comparison[i] <- 'REMOVE'
  }
}
x <- x[x$comparison != 'REMOVE',]

x$class <- factor(x$class,levels = c('Only shControl','Only shTwist2v1','Both'))

pdf(file = paste0(sample,'.FC.v.shControl.FC.linked.repressed.pdf'),width=4,height=4)
print(
  ggplot(x, aes(y=kd.fc , x=shC.fc,color=class)) +
    geom_point(size=2,alpha=0.5,stroke=0) +
    geom_abline(intercept = 0, slope = 1,color='black',size=1) +
    geom_abline(intercept = 0.118, slope = 1,color='black',alpha=0.5,size=1) +
    geom_abline(intercept = -0.118, slope = 1,color='black',alpha=0.5,size=1) +
    #geom_hline(yintercept=1, color = "red") +
    #geom_violin(alpha=0.5) +
    #geom_jitter(shape=16, col = 'light grey', position=position_jitter(0.2)) +
    theme_minimal() +
    labs(y = 'shTwist2v1 FC',
         x = 'shControl FC',
         title='All Repressed Genes in TWIST cis.links',
         color='Repressed in:') +
    #color = 'Near',alpha='Magnitude Total \n ATAC Change') +
    theme(
      #panel.grid.minor = element_blank(),
      #legend.position = 'none',
      plot.title = element_text(size=12,face='bold',hjust = 0.5),
      axis.ticks = element_blank(),
      axis.text.y = element_text(size=11,color='black',face='bold'),
      axis.text.x = element_text(size=11,color='black',face='bold'),
      axis.title = element_text(size=12,color='black',face='bold'),
      legend.text = element_text(size=10,color='black',face='bold'),
      legend.title = element_text(size=11,color='black',face='bold')) +
    scale_color_manual(values = c('lightblue','blue','dark blue')) +
    xlim(0, 1.21) +
    ylim(0, 1.21)
```

```
  #coord_cartesian(xlim = c(0, 1.21),ylim=c(0,1.21))
)
dev.off()
```
