# Supplementary material for: Kinetic networks identify TWIST2 as a key regulatory node in adipogenesis
Source: Genome Res. 2023 Mar;33(3):314–31. doi: 10.1101/gr.277559.122 (PMC10078291; doi:10.1101/gr.277559.122)
Supplement: Supplemental Material [file supp_gr.277559.122_Supplemental_Figures_Methods_.pdf]

Supplemental Methods

Genome browser visualization.

Genome browser (Kent et al. 2002) images were taken from the following track hub: http://guertinlab.cam.uchc.edu/adipo_hub/hub.txt. An identical track is reproduced on a public server: https://data.cyverse.org/dav-anon/iplant/home/guertin/adipo_hub/hub.txt

DNA binding domain alignments.

DNA binding domains were extracted from the TFClass database (Wingender et al. 2018) and TF paralogs that were absent from the database were extracted from the NCBI protein database. DNA binding domains were aligned using `FASTA` (Pearson and Lipman 1988) and the following command: `ssearch36 -s MD40 -m 8CBl`. Although there are six DNA motifs, the TWIST and ZNF families of DNA binding domains recognize the same motif, despite their lack of evolutionary conservation.

Compartment modeling.

Detailed analysis and raw code is available at https://github.com/guertinlab/modeling_PRO_composites. We calculated pause region signal by summing the PRO-seq signal over a 50 base pair window centered on the summit of the pause peak. The gene body RNA polymerase density was determined by averaging the PRO-seq signal over the region from the end of the pause window to the transcription termination site determined by `primaryTranscriptAnnotation`. We described RNA polymerase density dynamics in each compartment using differential equations that incorporate rates of transcription initiation, premature termination, pause release, and elongation. We determined how different rate constants would affect hypothetical pause region and gene body densities and the pause index using the `pksensi` package (Hsieh et al. 2020). We varied initiation, premature termination, and pause release rate constants from 0.01 to 1 molecules per second and varied elongation rate from 600-6000 bp/minute and calculated compartment density with each parameter set. We excluded any parameter estimates that result in more than 1 RNA polymerase molecule in the pause region at any given time. We selected all sets of parameters that resulted in the observed median composite pause index for all time points from Fig. 4. Next, we sought to determine if changing the rate constants could explain observed changes in compartment density at different gene sets for the indicated time point comparisons. We varied rate constants from the early time point values to model RNA polymerase density ratio changes between time points. We varied the initiation and pause release constants from their initial values over a 5-fold range in each direction and determined which sets of constants produced the observed changes in pause density ratio for each gene. We allowed the target ratio to vary by 5% compared to the observed. We plotted model RNA polymerase density curves by choosing the set of parameters with the elongation rate closest to a consensus rate of 2500 bases / min (Ardehali and Lis 2009; Jonkers and Lis 2015), while still accurately reproducing the composite profiles densities within 5% of the original. In order to reproduce composite plots, we spread the pause density over a 50 base region and fit the density profiles to a waveform as previously described (Sathyan et al. 2019). For the model, our only assumption is that elongation rate of a particular gene does not change between time points. In fact, if elongation rate was increasing in activated genes then we would expect a decrease in gene body PRO-seq signal. This finding has not been observed in other datasets. Of all the rate constants in the model, it is most reasonable to assume that the elongation rate stays constant while the others are affected by different regulatory mechanisms.

Twist2 Overexpression Construct Cloning.

Doxycycline-regulated expression of Twist2 was cloned by PCR into pEN_TT_miRc2 3xFLAG (Addgene #83274), then was verified by sequencing and LR recombined with pSLIK neo (Addgene #25735) for lentiviral packaging. pSLIK 3xFLAG-LacZ neo (Addgene #83105) was used as a control. The plasmids were deposited with Addgene (#197935 and #197936).

shRNA-Mediated Knockdown.

We purchased lentiviral shRNA-expressing constructs targeting Twist2 (Millipore Sigma clone IDs TRCN0000086084, TRCN0000086085, TRCN0000086086) and a non-mammalian control (Millipore Sigma SHC202). HEK-293T cells were transfected with shRNA constructs and lentiviral packaging constructs pMD2.G (Addgene #12259) and psPAX2 (Addgene #12260). We isolated and filtered supernatant after 24 and 48 hours. We transduced 3T3-L1s with virus in 8 μg/mL polybrene. Cells were switched into puromycin-containing selection media after 48 hours. After another 48 hours surviving cells were plated for further experiments.

Oil Red O staining.

For Oil Red O assays 3T3-L1s were cultured and differentiated as described above. During differentiation media was changed every 2 days. At day 6 of differentiation we stained cells with Oil Red O as previously described (Kraus et al. 2016). Briefly, cells were washed with PBS, fixed with 4% formaldehyde for 15 minutes, and stained with a 0.2% Oil Red O and 40% 2-propanol solution. After incubating for 30 minutes adipocytes were washed five times with distilled water and the dye was eluted with 2-propanol. Absorbance was measured at 540 nm using 2-propanol as a blank.

RNA extraction and RNA-seq.

3T3-L1s were cultured and differentiated as described above. At the indicated time points we harvested cells and extracted RNA using the Direct-zol-96 RNA kit (Zymo #11-331H). Samples were then sent to Novogene for bulk RNA-seq.

Immunoblotting.

3T3-L1s were cultured and differentiated as described above. Immunoblotting was performed on 7.5 µg RIPA lysate as previously described (Janes 2015). Samples were electrophoresed through 1.5-mm thick 12% polyacrylamide in tris-glycine running buffer (25 mM tris base, 250 mM glycine, and 0.1% SDS) at 130 V for 90 minutes. Proteins were transferred to a PVDF membrane (Millipore; Immobilon-FL, 0.45 mm pore size) in transfer buffer (25 mM tris, 192 mM glycine, 0.0375% SDS, and 20% methanol) at 100 V for 1 hour on ice. Membranes were blocked with 0.5X Odyssey blocking buffer in TBS. Primary antibodies were diluted in 0.5X Odyssey blocking buffer + 0.1% Tween-20. The following primary antibodies were used: Flag (Millipore Sigma F1804, 1:2000), HSP90 (Santa Cruz Biotechnology sc-13119, 1:2000), Tubulin (Cell Signaling Technology 2148S, 1:2000). Membranes were washed with TBS-T and exposed to fluorophore-conjugated secondary antibody diluted in 0.5X Odyssey blocking buffer + 0.1% Tween-20 + 0.01% SDS. Following another round of washing, membranes were scanned on an Odyssey infrared scanner (LI-COR).

Mouse experiments.

All mouse experiments were performed in accordance with the relevant guidelines and regulations of the University of Virginia and approved by the University of Virginia Animal Care and Use Committee. Mice were housed in specific pathogen-free conditions under standard 12-h-light/dark cycle conditions in rooms equipped with control for temperature (21±1.5°C) and humidity (50±10%). Twist2 knockout mice were purchased from Jackson Laboratories (Strain 008712). Preadipocytes were isolated from inguinal WAT as previously described (Galmozzi et al. 2021) from P3 mice. Preadipocyte differentiation and staining was carried out as with 3T3-L1s. Interscapular skin and BAT samples were isolated from P3 or P14 mice respectively. Tissue samples were fixed with 4% formaldehyde. Fixed tissues were paraffin-embedded and H&E stained by the Research Histology Core at the University of Virginia.

Supplemental References

Ardehali MB and Lis JT. 2009. Tracking rates of transcription and splicing in vivo. *Nature structural & molecular biology* **16**: 1123–1124.

Galmozzi A, Kok BP, and Saez E. 2021. Isolation and differentiation of primary white and brown preadipocytes from newborn mice. *JoVE (Journal of Visualized Experiments)* p. e62005.

Hsieh NH, Reisfeld B, and Chiu WA. 2020. pksensi: An r package to apply global sensitivity analysis in physiologically based kinetic modeling. *SoftwareX* **12**: 100609.

Janes KA. 2015. An analysis of critical factors for quantitative immunoblotting. *Science signaling* **8**: rs2–rs2.

Jonkers I and Lis JT. 2015. Getting up to speed with transcription elongation by rna polymerase ii. *Nature reviews Molecular cell biology* **16**: 167–177.

Kent WJ, Sugnet CW, Furey TS, Roskin KM, Pringle TH, Zahler AM, and Haussler D. 2002. The human genome browser at ucsc. *Genome research* **12**: 996–1006.

Kraus NA, Ehebauer F, Zapp B, Rudolphi B, Kraus BJ, and Kraus D. 2016. Quantitative assessment of adipocyte differentiation in cell culture. *Adipocyte* **5**: 351–358.

Pearson WR and Lipman DJ. 1988. Improved tools for biological sequence comparison. *Proceedings of the National Academy of Sciences* **85**: 2444–2448.

Sathyan KM, McKenna BD, Anderson WD, Duarte FM, Core L, and Guertin MJ. 2019. An improved auxin-inducible degron system preserves native protein levels and enables rapid and specific protein depletion. *Genes & development* **33**: 1441–1455.

Wingender E, Schoeps T, Haubrock M, Krull M, and Dönitz J. 2018. Tfclass: expanding the classification of human transcription factors to their mammalian orthologs. *Nucleic acids research* **46**: D343–D347.

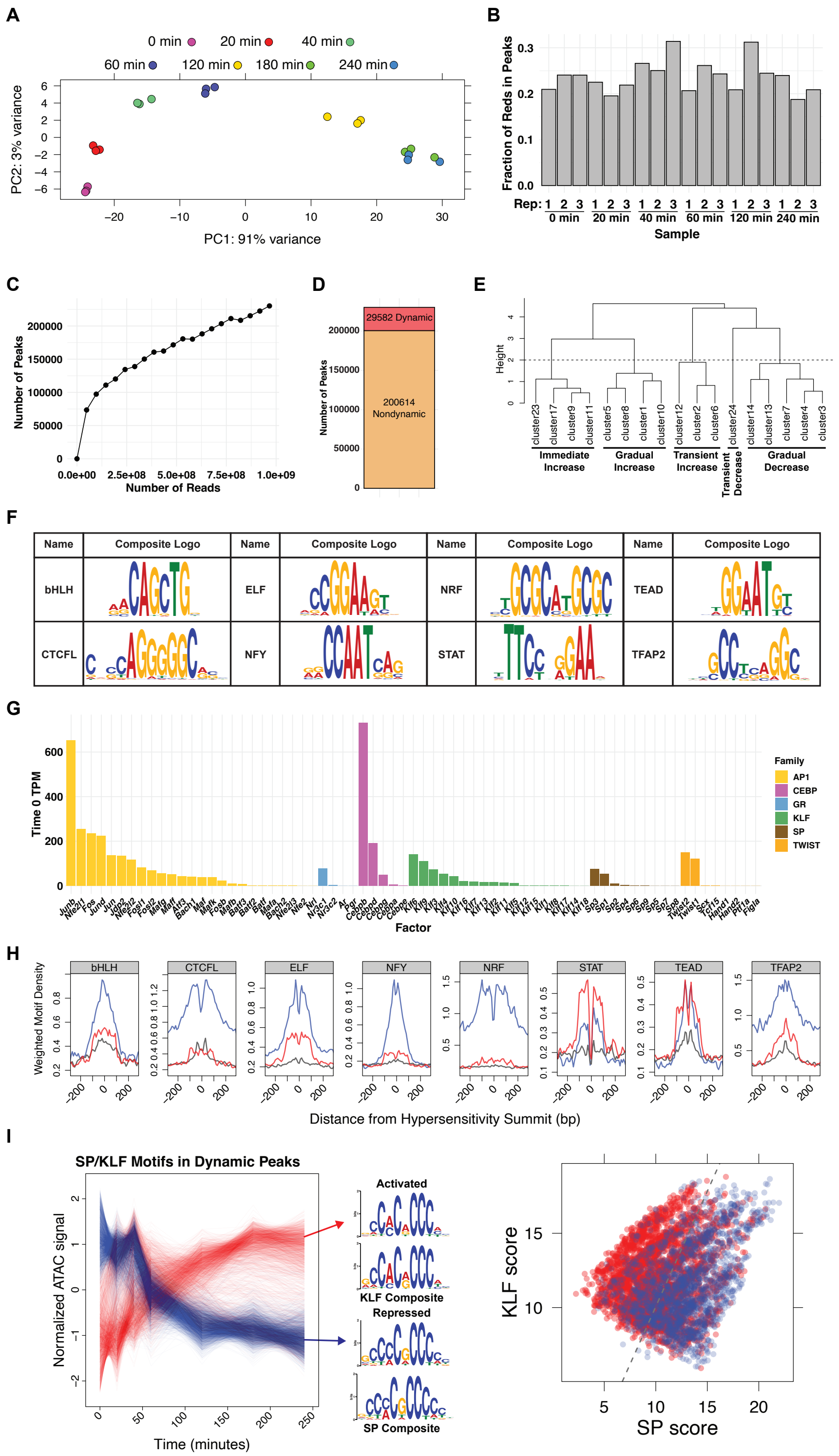

Supplemental Fig S1. Detailed analyses of dynamic ATAC-seq peaks reveals potential regulatory transcription factors A) Principal component analysis of ATAC-seq samples shows clear separation of samples by time. B) Fraction of reads within peaks does not vary significantly across samples. C) Calling peaks on progressively larger subsets of all reads leads shows the sequencing libraries are approaching saturation. D) We used a likelihood ratio test to define ATAC-seq peaks as dynamic or nondynamic using an adjusted p-value cutoff of 1×10^{-8}. E) After clustering ATAC-seq peaks based on accessibility dynamics, we combined clusters into more inclusive response classes by cutting the dendrogram at the indicated height of 2. F) In addition to the motifs discussed in Fig. 1, de novo motif analysis identified 8 factor families as potential regulators of ATAC-seq peak dynamics. These motifs are less enriched than those in Fig. 1. G) Expression of all members of potential regulatory TF families as measured by RNA-seq of 3T3-L1 cells before addition of differentiation media. H) We plotted enrichment of each motif family around peak summits as in Fig. 1E. I) Composite motif extraction on all peaks with either SP or KLF motifs returns a KLF-like motif in the increased peaks and an SP-like motif in the decreased peaks, suggesting that KLF and SP factor families are associated with increased and decreased accessibility respectively. We scored each SP/KLF motif in a dynamic peak against an SP composite and a KLF composite and plotted the two scores against one another. Each data point is colored based on the dynamics of the peak. The dividing line separates points such that all motifs on the left are labeled as KLF motifs and all motifs on the right are labeled SP motifs. The line is drawn in such a way as to maximize the number of increased KLF peaks and decreased SP peaks.

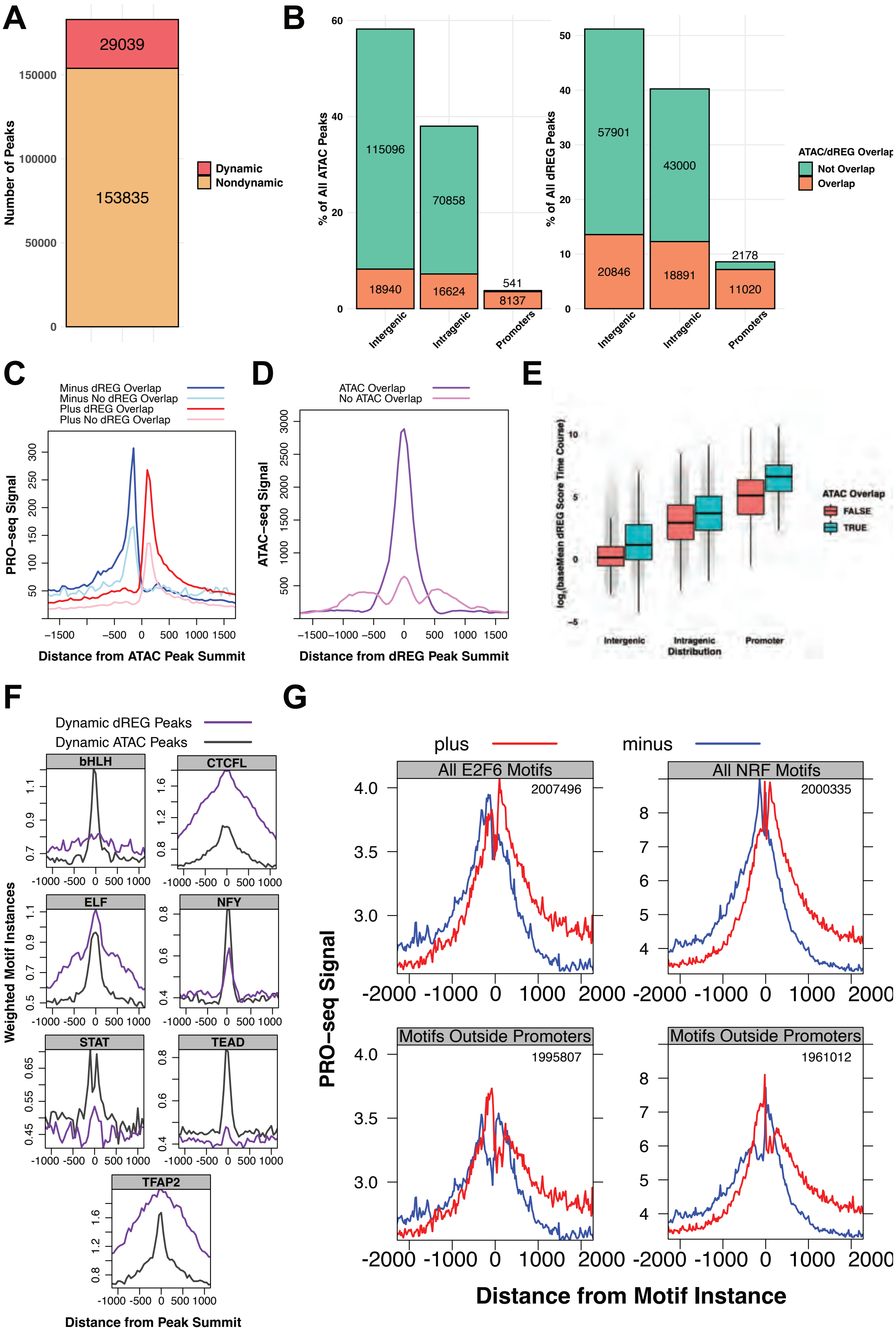

Supplemental Fig S2. dREG peak analysis reveals potential factors driving bidirectional transcription A) We used a likelihood ratio test to define dREG peaks as dynamic or nondynamic using an adjusted p-value cutoff of 1×10^{-5}. B) ATAC-seq and dREG-defined REs - including dynamic and nondynamic peaks - largely overlap in promoter regions. Genome regions are defined as described in Fig. 2 legend. Overlapping categories are not equal between ATAC and dREG peaks because multiple ATAC peaks may overlap a single dREG peak and vice versa. C) PRO-seq signal plotted around ATAC peaks separated by whether they overlap with dREG peaks. D) ATAC-seq signal plotted around dREG peaks separated by whether they overlap with ATAC peaks. E) dREG score for peaks sets stratified by location and ATAC overlap status. F) Motif density plots identify CTCF, ELF, and TFAP2 as enriched in dREG peak summits over ATAC-seq peak summits. Since these factors were not found in the de novo analysis, we did not consider them dREG-enriched factors. G) Plotting PRO-seq signal around non-SP dREG factor motifs shows the bidirectional transcription phenotype but not as clearly as with the SP motifs.

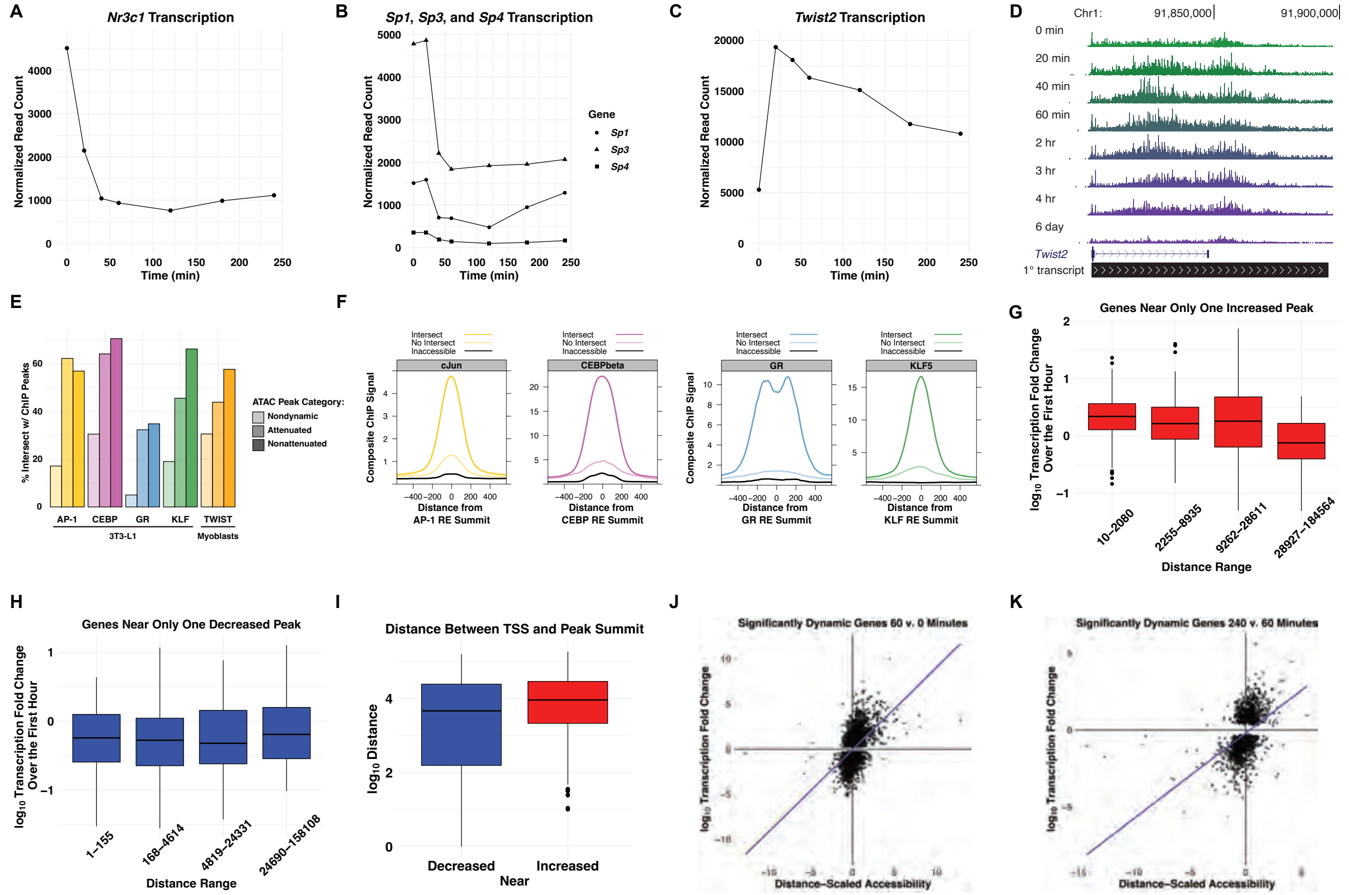

Supplemental Fig S3. Gene transcription positively correlates with local accessibility dynamics A-C) Normalized PRO-seq signal for A) the *Nr3c1* gene, B) the *Sp1*, *Sp3*, and *Sp4* genes, and C) the *Twist2* gene. *Nr3c1*, *Sp1*, *Sp3*, and *Sp4* all exhibit rapid early repression, while *Twist2* is immediately and transiently activated. D) The UCSC Genome Browser shot shows that early activation of *Twist2* gene has subsided by the time cells reach full maturity at 6 days. E) We compared our ATAC-seq derived REs with ChIP-seq peaks for AP-1 (C-JUN and JUNB), KLF (KLF4 and KLF5), CEBPβ, and GR in 3T3-L1 as well as TWIST2 in myoblasts. ATAC-seq peaks are divided into nondynamic peaks that are not inferred binding events, attenuated peaks that increase early in the time course but decrease toward the end, and nonattenuated peaks that do not decrease in accessibility at any point. F) Composite ChIP-seq signal is plotted at indicated genomic regions. Regions are classified into dynamic REs that intersect with ChIP-seq peaks, dynamic REs that do not intersect with ChIP-seq peaks, and TF motifs that are not found in ATAC-seq peaks and are therefore inaccessible. G-H) Box and whisker plots representing genes within 10 kb of either (G) a single increased peak or (H) a single decreased peak. Genes were split into four quartiles based on the distance between the peak summit and the TSS of the gene. Box and whisker plots on the left contain the smallest distances, while those on the right represent the largest. The y-value is the transcription of the genes over the first hour of the time course. We find that for genes near increased peaks, the closer the peak is to the gene the more likely gene transcription and peak accessibility dynamics will covary. We do not find a similar relationship for genes near decreased peaks. I) Box and whisker plots representing genes within 10 kb of either a single increased peak (red plot) or a single decreased peak (blue plot). The y-value represents the distance between the peak summit and the TSS of the gene. Genes near decreased peaks are generally closer to the peak summit than the increased peak-gene pairs. J-K) Scatter plot of genes dynamically transcribed between J) 60 and 0 minutes or K) 240 and 60 minutes. Each data point represents a gene. The x-value is total accessibility change over the time period scaled by the distance between the TSS and each peak. Positive x-values indicate increases in local accessibility. The y-value is change in transcription over the same time period. A gene with positive x and y-values or negative x and y-values exhibits positive covariation between local accessibility and transcription dynamics. The majority of genes for both time point comparisons exhibit positive covariation, indicating a correlative relationship between accessibility and transcription. Chi-squared p-values are (J) 1.41×10^{-98} and (K) 4.35×10^{-54}.

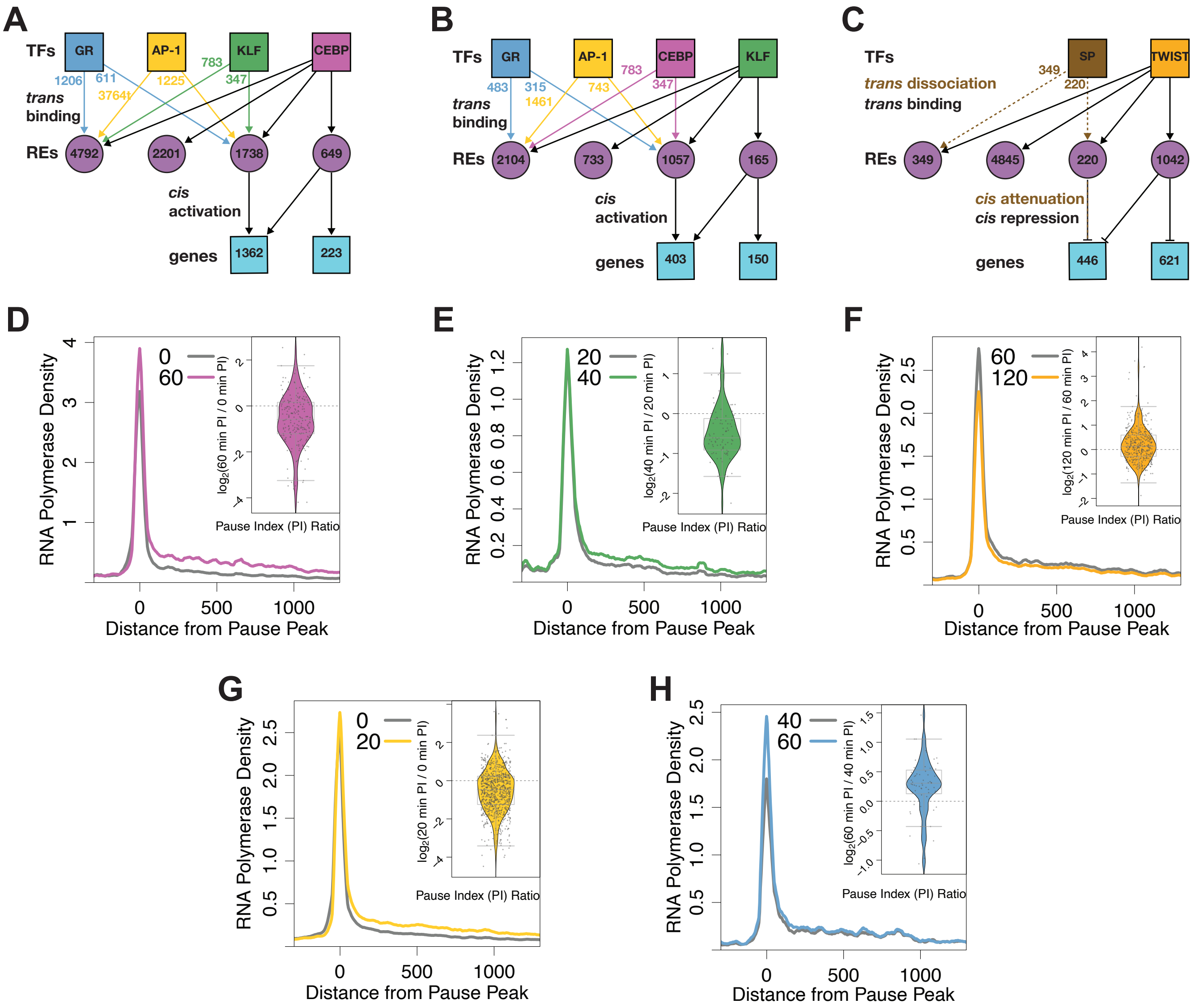

Supplemental Fig S4. Modular networks downstream of CEBP, KLF, and TWIST identify genes and transcriptional steps regulated by individual factors Modular networks downstream of (A) CEBP, (B) KLF, and (C) TWIST. Networks constructed as described in Fig. 4. Composite PRO-seq signal plotted around pause peak summits of (D) 223 genes solely regulated by CEBP, (E) 150 genes solely regulated by KLF, (F) 621 genes solely regulated by TWIST, (G) 1224 genes solely regulated by AP-1, and (H) 174 genes solely regulated by GR at the indicated time points illustrate average changes in RNA polymerase density within the pause region and gene bodies. Inset violin plots indicate pause indices as described in Fig. 4.

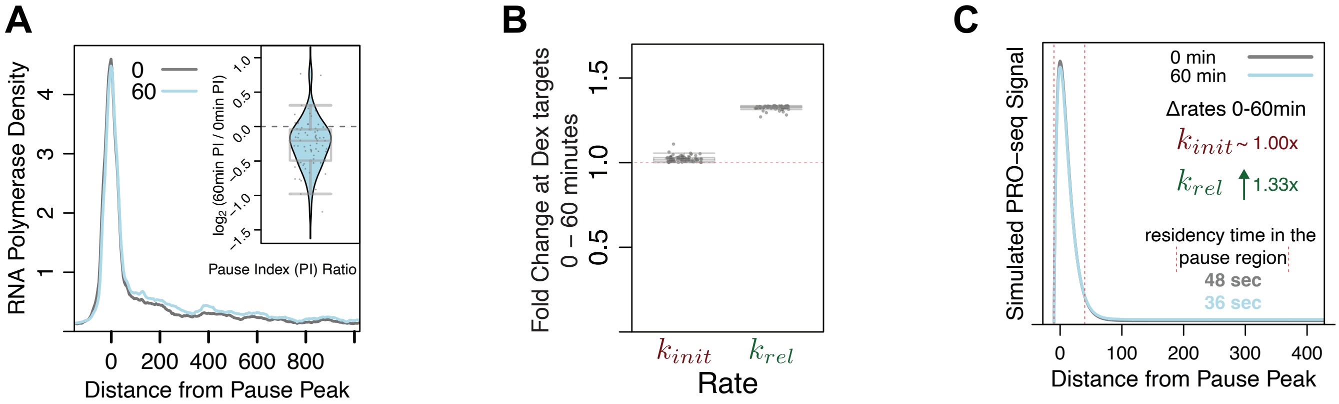

Supplemental Fig S5. Dexamethasone-activated genes in C7 cells recapitulate findings at predicted GR target genes in 3T3-L1 differentiation A) Composite polymerase density at 70 genes activated by dexamethasone treatment in C7 cells at indicated times. B) Compartment modeling of the activated genes holding k_{pre} and k_{rel} constant. We find an approximate 1.33-fold increase in pause release rate explains the observed changes in polymerase density. C) A simulated composite derived from the parameters estimated in (B).

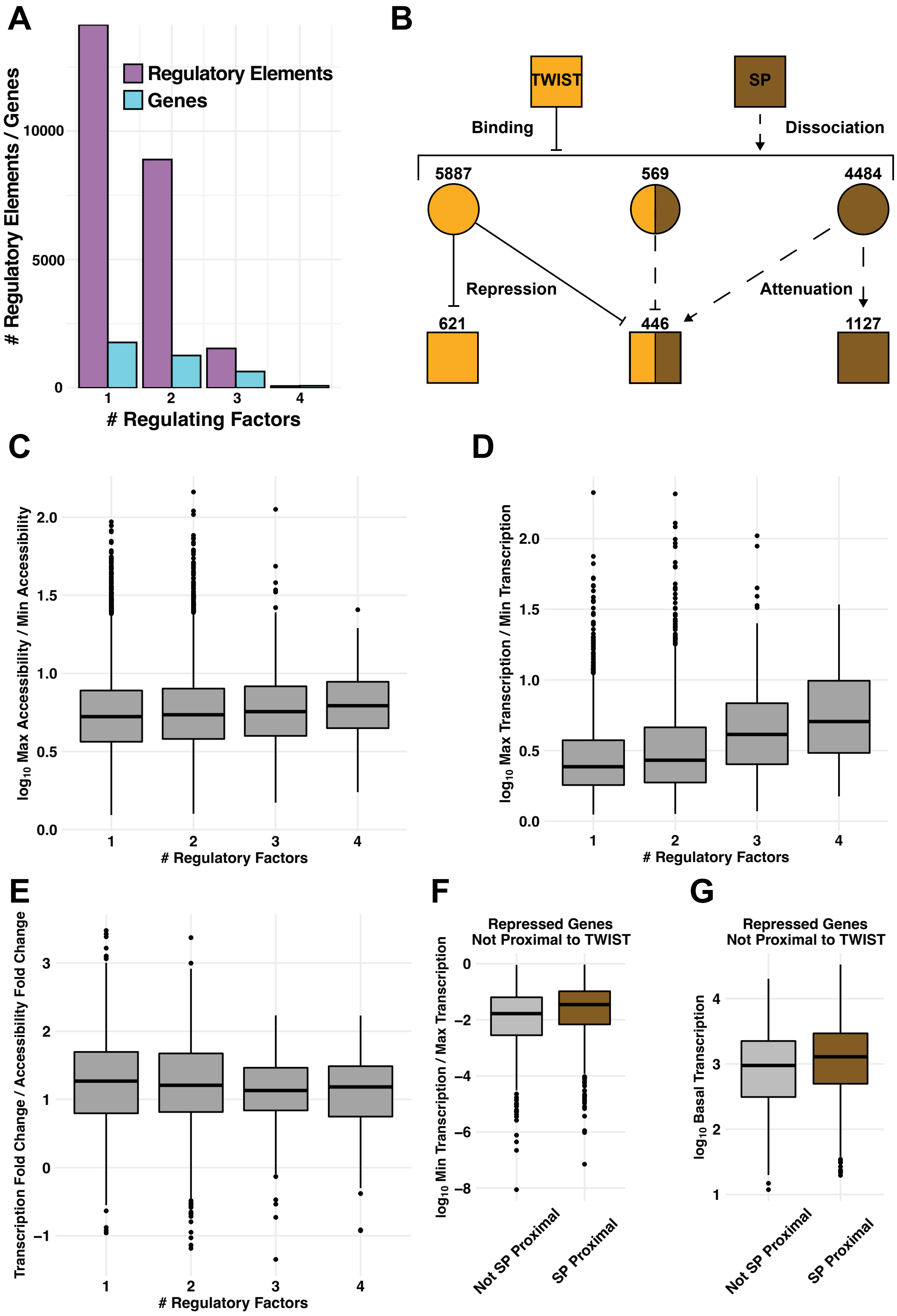

Supplemental Fig S6. Total local accessibility influences magnitude of gene transcription changes A) The bar plot quantifies the number of REs and genes that are activated by the indicated number of activating factors: GR, AP-1, CEBP, or KLF. B) This network depicts REs and genes downstream of TWIST binding and repression and/or SP dissociation and attenuation. C) Data points represent all REs bound by AP-1, CEBP, GR, and KLF. REs are stratified based on number of binding factors. Y-values are the fold change in accessibility. D-F) Data points represent all genes activated by AP-1, CEBP, GR, and TWIST. D) Genes are stratified based on number of upstream regulatory factors. Y-values are the fold change in nascent transcription. There is a positive correlation between transcriptional change and number of regulatory factors. E) Genes are stratified as in (D), but y-values represent fold change in transcription divided by fold change in local accessibility. There is no correlation between normalized transcription and number of regulatory factors when controlling for changes in accessibility. F) Repressed genes proximal to SP motifs tend to exhibit a lower magnitude of transcriptional change. G) Repressed genes proximal to SP motifs tend to exhibit higher basal transcription.

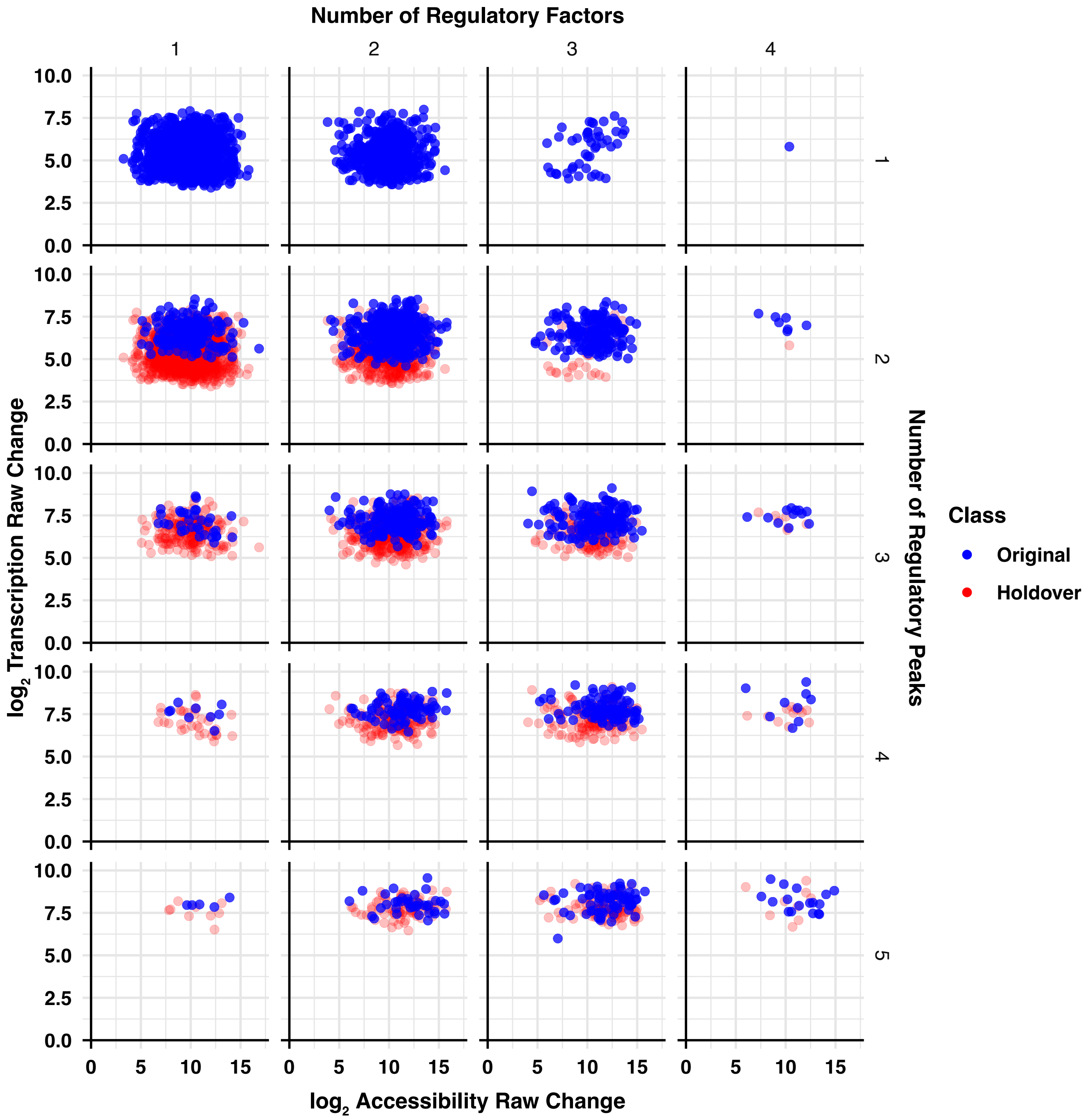

Supplemental Fig S7. Number of regulatory factors does not influence magnitude of gene transcription changes All predicted activated target genes are stratified by number of regulatory peaks (rows) and number of regulatory factors (columns). The y-axis indicates the log fold change in transcription for each gene for the time range over which the gene exhibits its greatest activation. Transcription change is plotted against total local accessibility change over the same comparison. Blue points represent the indicated number of regulatory factors and regulatory peaks. Red points represent the genes from the same number of regulatory factors but one fewer regulatory peaks in order to illustrate the effects of an increasing number of peaks. Number of regulatory peaks, and by extension total local accessibility, influences gene transcription. Conversely, the number of regulatory factors does not affect transcription.

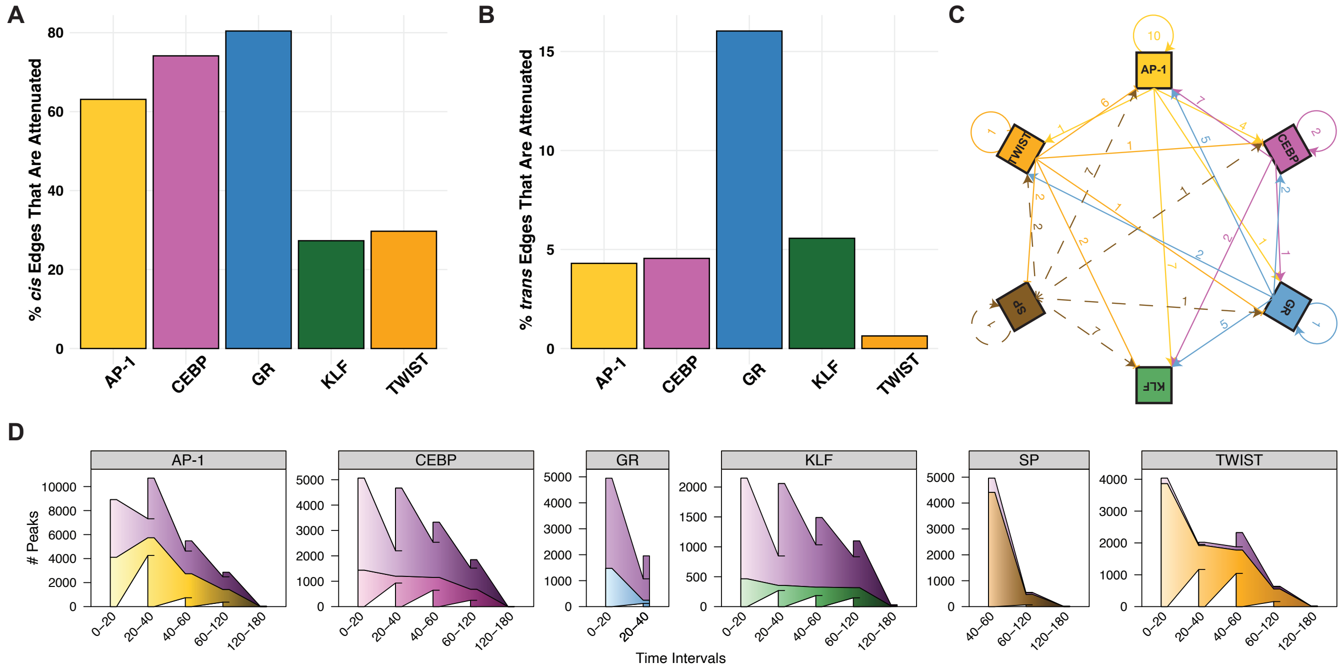

Supplemental Fig S8. TF families are transiently regulated by other factors, interconnected with one another, and exhibit distinct binding and dissociation kinetics A) The percentage of each factor's attenuated *cis*-edges highlights the transient nature of gene expression changes in adipogenesis. Early activators show the highest proportion of attenuated edges, meaning early activation is followed by a return to baseline expression. There are no attenuated SP *cis*-edges, meaning genes with decreased expression downstream of SP dissociation do not return to baseline within the time course. B) The percentage of each factor's attenuated *trans*-edges indicates that binding is more stable than gene expression changes. GR *trans*-edges are most likely to be attenuated. There are no attenuated SP *trans*-edges. C) TF genes are highly interconnected; arrows represent direct regulatory relationships between factors. Arrows emanate from regulatory factor family and point to target factor family. Solid arrows represent gene activation, blunt-ended arrows represent repression, and dashed arrows represent attenuation. The numbers indicate how many TF family members exhibit the indicated regulatory relationship. For example, the arrow from AP-1 to KLF represents the 7 KLF family genes activated by AP-1 factors. D) Wedged bar plots quantify the regulatory kinetics across the time course for indicated factors. The x-axis intervals represent the time range in which the specified factor regulates changes in accessibility of the indicated number of peaks (y-axis). Wedges between bars indicate carryover peaks from previous time interval and the outer "wings" represent peaks that are not included in the previous time interval. The top shaded purple wedges represent peaks regulated by multiple factors; bottom wedges represent peaks that are solely regulated by the indicated factor.

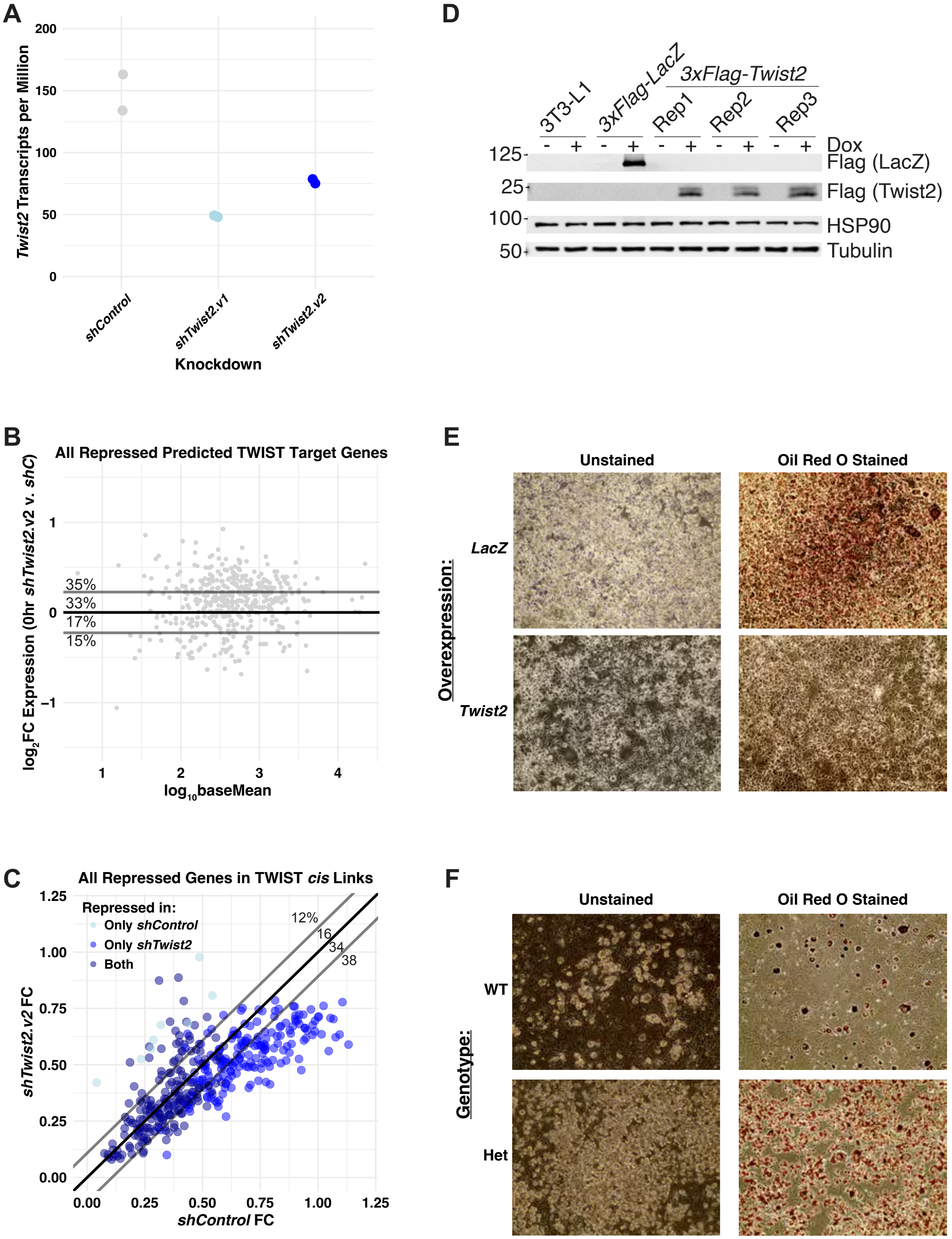

Supplemental Fig S9. ***Twist2*** **depletion derepresses target genes** A) Baseline expression of predicted TWIST2 target genes repressed in the RNA-seq time course plotted as in Fig. 8A. B) Fold change of predicted TWIST2 target genes repressed in the RNA-seq time course plotted as in Fig. 8A. C) Similar results with a moderate *Twist2* knockdown further support our hypothesis that TWIST2 acts as a repressor of gene expression and negative regulator preadipocyte differentiation. D) Immunoblotting indicating our tetracycline-inducible 3xFlag-tagged *Twist2* / *LacZ* constructs work appropriately. HSP90 and Tubulin are used as loading controls. E & F) Images taken at 10× magnification of either (E) 3T3-L1 overexpressing the indicated protein or (F) primary preadipocytes harvested from mice with the indicated genotype. Adipocytes are either unstained (left) or stained with Oil Red O (right).
